# Combinatorial assembly and optimisation of designer cellulosomes: a galactomannan case study

**DOI:** 10.1186/s13068-022-02158-2

**Published:** 2022-05-30

**Authors:** Julie Vanderstraeten, Maria João Maurício da Fonseca, Philippe De Groote, Dennis Grimon, Hans Gerstmans, Amaranta Kahn, Sarah Moraïs, Edward A. Bayer, Yves Briers

**Affiliations:** 1 Laboratory of Applied Biotechnology, Department of Biotechnology, Ghent University, Valentin Vaerwyckweg 1, 9000 Ghent, Belgium; 2 Department of Biomolecular Sciences, The Weizmann Institute of Science, 7610001 Rehovot, Israel; 3 Department of Life Sciences and the National Institute for Biotechnology in the Negev, Ben-Gurion University of the Negev, 8499000 Beer-Sheva, Israel; 4 Present Address: Laboratory for Biomolecular Discovery and Engineering, Department of Biology, VIB-KU Leuven Center for Microbiology, Kasteelpark Arenberg 31, 3001 Louvain, Belgium; 5 Present Address: Interdisciplinary Centre of Marine and Environmental Research (CIIMAR/CIMAR), University of Porto, Avenida General Norton de Matos, s/n, 4450-208 Matosinhos, Portugal

**Keywords:** VersaTile, DNA assembly, Designer cellulosome, Multi-enzyme complex, Scaffoldin, Hemicellulase, Galactomannan, Mannanase, Mannosidase, Galactosidase

## Abstract

**Background:**

Designer cellulosomes are self-assembled chimeric enzyme complexes that can be used to improve lignocellulosic biomass degradation. They are composed of a synthetic multimodular backbone protein, termed the scaffoldin, and a range of different chimeric docking enzymes that degrade polysaccharides. Over the years, several functional designer cellulosomes have been constructed. Since many parameters influence the efficiency of these multi-enzyme complexes, there is a need to optimise designer cellulosome architecture by testing combinatorial arrangements of docking enzyme and scaffoldin variants. However, the modular cloning procedures are tedious and cumbersome.

**Results:**

VersaTile is a combinatorial DNA assembly method, allowing the rapid construction and thus comparison of a range of modular proteins. Here, we present the extension of the VersaTile platform to facilitate the construction of designer cellulosomes. We have constructed a tile repository, composed of dockerins, cohesins, linkers, tags and enzymatically active modules. The developed toolbox allows us to efficiently create and optimise designer cellulosomes at an unprecedented speed. As a proof of concept, a trivalent designer cellulosome able to degrade the specific hemicellulose substrate, galactomannan, was constructed and optimised. The main factors influencing cellulosome efficiency were found to be the selected dockerins and linkers and the docking enzyme ratio on the scaffoldin. The optimised designer cellulosome was able to hydrolyse the galactomannan polysaccharide and release mannose and galactose monomers.

**Conclusion:**

We have eliminated one of the main technical hurdles in the designer cellulosome field and anticipate the VersaTile platform to be a starting point in the development of more elaborate multi-enzyme complexes.

**Supplementary Information:**

The online version contains supplementary material available at 10.1186/s13068-022-02158-2.

## Background

Lignocellulose is a major component of plant cell walls and is abundant in nature. Lignocellulosic biomass is composed of cellulose, hemicellulose and lignin. Both cellulose and hemicellulose are composed of sugar monomers that can be converted to biofuels, bio-based materials and/or interesting chemicals [1]. However, lignocellulosic biomass is a recalcitrant substrate. Hemicelluloses interact with cellulose via hydrogen bonds and attach to the surface of cellulose microfibrils. Additionally, hemicelluloses can be cross-linked to lignin, which in turn interacts with cellulose, creating a highly compact matrix. In addition, cellulose is highly crystalline and the high heterogeneity of the lignocellulose substrate requires several synergistically acting enzymes for its breakdown [2]. Because of its high recalcitrance and complexity compared to starch-derived sugars, saccharification of lignocellulose into fermentable sugars has long since represented an important technical and economical challenge.

In nature, selected specialist micro-organisms use plant cell walls as an energy source and are able to enzymatically break down lignocellulosic biomass with the help of multi-enzyme complexes, termed cellulosomes. These ‘nanomachines’ consist of two complementary structural modules. A large backbone molecule, termed the scaffoldin, comprises a carbohydrate binding module (CBM) and several different cohesin modules. Docking enzymes consist of a dockerin module and a catalytic enzyme [3]. A species-specific interaction between cohesin modules of the scaffoldin and dockerin modules of the enzymes ensures the arrangement of different catalytic activities on a single backbone. As such, all catalytic modules with complementary functions are positioned close to each other, thus enhancing their synergism [4]. Because cellulosomes are highly efficient in degrading lignocellulosic biomass, these enzyme complexes have attracted the interest of researchers to adapt and use them in the lignocellulosic conversion process. The goal is to use the knowledge about naturally occurring enzyme complexes to create engineered designer cellulosomes with the potential to degrade all lignocellulosic components and to convert these degradation products into valuable end products. By combining cohesin-dockerin pairs from different species, the protein engineer is able to control the composition, arrangement and copy number of the selected enzymes.

Over the years, research in the designer cellulosome field has gradually increased in complexity. Initially, docking enzymes were selectively incorporated into defined functional complexes [5]. Later, complete free enzyme systems were transformed to the cellulosomal mode [6]. In recently published studies, multiple variants of designed docking enzymes and scaffoldins were analysed [7, 8]. As such, it became clear that many different parameters affect the activity of these multi-enzyme complexes. Recently, we reviewed the different parameters influencing the activity of designer cellulosome complexes and concluded that there are currently no conserved design rules and that the optimal parameters depend on the selected enzymes [9]. Designer cellulosome activity has been shown to depend on the composition and order of the docking enzymes [7], the thermostability of the enzymes [10] and post-translational modification of the incorporated proteins [11]. This implies that a customised approach needs to be followed for the development of each individual designer cellulosome, which is a particularly tedious procedure, due to the high modularity and extensive DNA work required to prepare all components. Hence, the optimisation of a designer cellulosome remains an empirical process. A practically infinite number of modular combinations, in terms of selected enzymes, selected cohesin/dockerin pairs, intervening linkers, specific order, etc., can be envisioned. In an extensive study by Vazana et al. (2013), a synthetic biology approach was followed to systematically investigate the spatial organisation of the scaffoldin subunit [12]. For this purpose, 72 scaffoldin variants were designed. Of these, 16 scaffoldin variants could not be constructed owing to difficulties in cloning. Indeed, standard cloning techniques do not allow fast and efficient construction of many different modular protein variants [13], demonstrating that there is a need for a technique to construct multiple docking enzymes and scaffoldin variants in a more efficient manner. In addition, the relatively large size of both scaffoldins and some of the docking enzymes renders the use of commercial gene synthesis prohibitively expensive to explore a large combinatorial design space.

Our research group has developed a combinatorial DNA assembly method, termed VersaTile, dedicated to the efficient and convenient construction of the coding sequences of modular proteins at a high rate [14]. Since cellulosomes are an exponent of modularity, the VersaTile technique could be highly appropriate to eliminate the current technical constraints in designer cellulosome research. Here, we illustrate the extension of the VersaTile platform to facilitate the construction of designer cellulosomes. We describe the design of two distinct assembly systems (one specifically for docking enzymes and another specifically for scaffoldins), the development of a tile repository and the construction of several destination vectors. As a proof of concept, we then illustrate the multiparametric optimisation of a cellulosome complex specifically designed for galactomannan (GM) degradation. GM is an important hemicellulosic polysaccharide found in softwoods and many other plant sources. The degradation of GM is therefore crucial for the efficient saccharification of lignocellulose of softwoods and accordingly for the production of second-generation biofuels [15]. Mannans are also stored in endosperm walls and vacuoles of seeds and vegetative tissues (e.g., ivory nut, coconut, coffee bean) [16]. As these plants are widely used in the food industry, large volumes of mannan-rich materials are discarded as waste in the food supply chain, underpinning the usefulness of mannan-degrading enzymes for a bio-based economy [17]. In the present work, we followed a two-tiered approach: first, the different docking enzymes (mannanase, mannosidase, galactosidase) were constructed, evaluated and optimised. Subsequently, multiple trivalent designer cellulosomes were constructed by varying the scaffoldin protein. The optimised designer cellulosome was able to degrade the GM substrate and release galactose and mannose monomers in an efficient manner.

## Results

### VersaTile—a method for rapid docking enzyme and scaffoldin construction

VersaTile is a DNA assembly method that was developed to eliminate the current technical constraints to construct multimodular proteins in a combinatorial way. It is a Lego-like assembly method specifically for modular proteins that do not share DNA homology [14]. VersaTile follows a two-step approach (Additional file 1: Fig. S1). First, a repository of all tiles is constructed. Second, any assembly with a selection of these tiles can be created in a one-step reaction with short hands-on time and high efficiency. A tile is defined as a coding sequence for a specific module that is made compatible with the VersaTile technique. The coding sequence is therefore flanked by six-nucleotide long position tags and BsaI recognition sites, and cloned in a dedicated entry vector, pVTE. BsaI cleavage within the position tags generates position-specific, single-stranded overhangs that are joined in a dedicated destination vector, pVTD. Hands-on time is limited to pipetting each tile along with an appropriate destination vector, BsaI and T4 DNA ligase, followed by a cyclic temperature protocol and a transformation step.

Designer cellulosomes are composed of two types of modular proteins: (1) The scaffoldin, which serves as the backbone molecule, is composed of multiple cohesins and in many cases a CBM. (2) Multiple docking enzymes, composed of a dockerin module and one or two enzymatically active modules, are selectively incorporated into the scaffoldin by virtue of the cohesin-dockerin interaction. For both multimodular proteins, a separate VersaTile assembly system was developed.

First, we designed a three-way system allowing the construction of docking enzymes composed of three modules (Additional file 1: Fig. S2). Additional file 1: Table S1 shows the position tags employed in the three-way system. The position tags encode two amino acids that will act as linkers between the selected modules. Second, we developed a five-way system allowing the construction of scaffoldins composed of five modules (Additional file 1: Fig. S3). Additional file 1: Table S2 shows the position tags employed in the five-way system. We note that BsaI cleavage within the first and last position tags generates single-stranded overhangs that are the same for both systems, therefore indicating that the same collection of destination vectors can be used for both docking enzyme and scaffoldin assembly systems. The docking enzyme assembly system allows the construction of docking enzymes composed of three modules. These could for example be an enzyme, a linker and a dockerin, or two enzymes and a dockerin. However, in some cases, it would be interesting to construct a docking enzyme composed of an enzyme, a dockerin and no linker. To compare the activity of constructed docking enzymes with the natural enzyme, the system should preferably also allow for the construction of proteins composed of a single enzymatic module without dockerin. For this purpose, we designed His-tag tiles flanked by different combinations of position tags. Use of these tiles allows the conversion of the three-way system to a two-way system (Additional file 1: Fig. S4). The same approach was followed to convert the five-way scaffoldin system into a four-, three- or two-way system (Additional file 1: Fig. S5). In addition to the twelve His-tag tiles, we also prepared tiles encoding a Glutathione S-transferase (GST) and StrepII tag. These tiles were constructed to be fitted at the first position of the scaffoldin assembly system.

Upon the development of the two VersaTile construction systems, two separate tile repositories were generated. The first tile repository contains building blocks needed for the construction of docking enzymes (dockerins, linkers and enzymatically active modules). The second tile repository contains the building blocks needed for the construction of scaffoldins (CBMs, cohesins and tags). First, we filled the tile repositories with the non-catalytic cellulosomal building blocks. Seven cohesins and eight dockerins were selected from naturally occurring cellulosome-producing organisms (Additional file 1: Table S3). Next, a series of tiles encoding enzymes active on the different constituents of lignocellulosic biomass was added to the repository. Additionally, the *Clostridium thermocellum* CipA CBM3a (*Ct*-CBM3) (Additional file 1: Table S4) was added. Finally, nine linker tiles were added to the docking enzyme tile repository (Additional file 1: Table S5). These tiles were designed specifically for the second position in docking enzyme constructs.

Cloning in the standard destination vector (pVTD2) results in the expression of a protein consisting solely of the selected tiles. Our repository contains multiple variants of this destination vector, allowing the N- or C-terminal addition of the CBM3a module, His-, StrepII- and/or GST-tag (Additional file 1: Fig. S6). Enhancing the variety of available destination vectors further improves the flexibility of the VersaTile platform. Whereas assembly in pVTD2 allows the construction of scaffoldins composed of five modules, this number is increased to seven when assembling in more complex destination vectors such as pVTD17. Since this vector already integrates a GST and CBM module in its backbone, five cohesin tiles can be selected for assembly. In some cases, the additional tags are cleavable.

### VersaTile allows the production and optimisation of designer cellulosomes: galactomannan case study

Based on the selected target lignocellulose component, a range of tiles encoding specific lignocellulosic enzymes can be selected from the repository, and a designer cellulosome composed of these catalytic modules can be optimised. Here, we selected galactomannan (GM), a specific hemicellulose primarily found in softwoods. GM consists of a backbone composed of β-1,4-linked d-mannose residues and α-1,6-linked d-galactose side chains. β-Mannanase catalyses the random hydrolysis of the β-1,4-d-mannosidic linkages in the GM backbone. β-Mannosidase catalyses the hydrolysis of the glycosidic bond of the terminal, non-reducing β-d-mannose residues in β-d-manno-oligosaccharides (MOS). The enzyme α-galactosidase catalyses the hydrolysis of the α-1,6-linked galactose side chains.

Five GM-degrading enzymes were present in the docking enzyme tile repository. In previous research, many *Thermobifida fusca* enzymes have been successfully converted to the cellulosomal mode [6, 18, 19]. We therefore selected the *T. fusca* β-mannanase (GH5) and β-mannosidase (GH2) as the two backbone-degrading enzymes. Both enzymes have been characterised before [20–22]. Whereas the natural mannanase comprises a GH5 enzymatic module and a family 2 CBM, the mannosidase does not carry additional modules. Our preliminary activity analysis revealed that the preferred substrate of the mannanase is GM, followed by mannan and MOS. The mannosidase prefers p-nitrophenyl β-d-mannopyranoside (pNP-β-mannose), followed by MOS, mannan and GM. To date, no *T. fusca* α-galactosidase has been characterised. Three α-galactosidases from different origins were thus selected as putative candidates to work synergistically with the selected backbone-acting enzymes. *Clostridium cellulolyticum* α-galactosidase is a multimodular enzyme composed of an N-terminal GH27 enzymatic module, a family 6 CBM and a C-terminal dockerin. *Bifidobacterium adolescentis* α-galactosidase is a GH36 enzyme. *Cellvibrio japonicus* α-galactosidase is a GH27 enzyme. Each tile was amplified without its native signal peptide. An overview of the tiles encoding GM-degrading enzymes can be found in Additional file 1: Table S6. We note that the selected enzymes originate from both mesophilic (*C. cellulolyticum*, *C. japonicus* and *B. adolescentis*) and thermophilic bacteria (*T. fusca*). Based on the optimal temperature (range) of each of these enzymes, 50 °C was selected as the reaction temperature for all activity assays. Although we expect all enzymes to show activity at this temperature, it is only the exact optimum for one of the selected enzymes (*Cj*-Aga). Whereas the optimal temperature of *Tf*-Manna, *Tf*-Manno and *Ba*-Aga is above the selected reaction temperature, the optimal temperature of *Cc*-Aga is slightly below 50 °C. This might influence the ‘optimal’ composition of the designer cellulosome and the final complex is expected to include the enzymes most active specifically at this selected intermediate temperature. Since the *T. fusca* β-mannanase and *C. cellulolyticum* α-galactosidase are multimodular in nature, we constructed multiple tile variants for these enzymes. Whereas the shortest tile variant encodes the enzymatically active module, the longer variants encode (part of) the linker or the linker and the CBM module. Finally, we also added the *Cj*-CBM35 tile which originates from the *Cellvibrio japonicus* Man5C mannanase enzyme and has been described as mannan-specific [23, 24] (Additional file 1: Table S4).

Tables 1 and 2 show the number of tiles that were prepared at each position in the docking enzyme and scaffoldin assembly systems, respectively. These numbers allow us to calculate the theoretical number of docking enzymes and scaffoldins that can be constructed. For example, the tile repository allows the construction of 630 (= 10 × 9 × 7) monovalent docking enzymes composed of an N-terminal enzymatic module, a C-terminal dockerin and a linker separating these two modules. A total of 720 (= 8 × 10 × 9) bicatalytic docking enzymes composed of an N-terminal dockerin followed by two enzymatically active modules can be constructed. Regarding the scaffoldins, a total of two CBM tiles and seven cohesin tiles allows us to construct 28 (= (2 × 7) × 2) monovalent (one CBM and one cohesin), 294 (= (2 × 7 × 7) × 3) bivalent (one CBM and two cohesins), 2744 (= (2 × 7 × 7 × 7) × 4) trivalent (one CBM and three cohesins) and 24,010 (= (2 × 7 × 7 × 7 × 7) × 5) tetravalent scaffoldins (one CBM and four cohesins). Note that these numbers are calculated with a fixed number of one CBM per scaffoldin allowing all possible orders and assuming the use of the standard destination vector (pVTD2) that adds no additional modules. However, our repository also contains a range of different destination vectors (Additional file 1: Fig. S6) that integrate the *Ct*-CBM3a module in their backbone (pVTD14, pVTD15, pVTD16 and pVTD17), allowing the selection of five cohesin tiles for assembly. Use of one of these vectors allows us to construct 16,807 (= 7 × 7 × 7 × 7 × 7) pentavalent scaffoldins (one fixed N-terminal CBM module originating from *C. thermocellum* in the vector backbone and five assembled cohesins).

**Table 1:** Number of dockerins, enzymes and linkers present in the docking enzyme tile repository

	Icon	Position 1	Position 2	Position 3	Total
Dockerins		8	8	7	23
Enzymes		10	10	9	29
Linkers		/	9	/	9
Total		18	27	16	61

The first column shows the tile type. The second column shows the icons that are used to indicate the different tile types in this work. The third, fourth and fifth columns indicate the number of tiles that are present at positions 1, 2 and 3, respectively. The sixth column and last line give the total amount of tiles of this type that is present in the repository. See Additional file 1: Tables S3, S5 and S6 for description of the icons

**Table 2:** Number of cohesins and CBMs present in the scaffoldin tile repository

	Icon	Position 1	Position 2	Position 3	Position 4	Position 5	Total
Cohesins		7	7	7	7	7	35
CBMs		2	2	2	2	2	10
Total		9	9	9	9	9	45

The first column shows the tile type. The second column shows the icons that are used to indicate the different tile types in this work. The third, fourth, fifth, sixth and seventh columns indicate the number of tiles that are present at positions 1, 2, 3, 4 and 5, respectively. The eighth column and last line give the total amount of tiles of this type that is present in the repository. See Additional file 1: Tables S3 and S4 for description of the icons

**Fig. 1:**
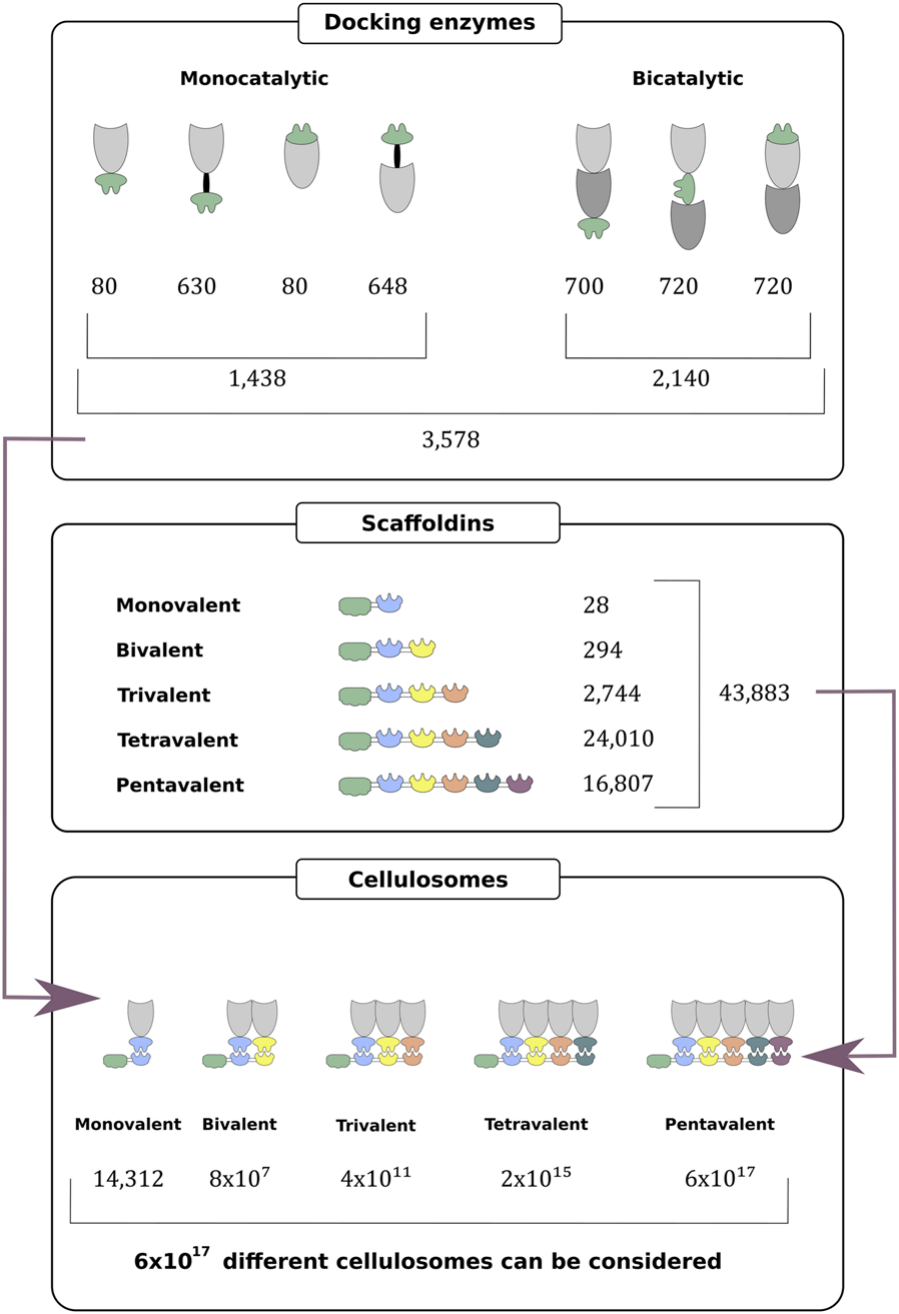
Combinatorial power of VersaTile. The docking enzyme assembly system allows the construction of monocatalytic and bicatalytic docking enzymes. The position of the dockerin and enzymes can be chosen freely. Monocatalytic docking enzymes can be constructed with or without a linker separating the dockerin and enzymatically active module. The docking enzyme tile repository allows the construction of 3578 GM-degrading docking enzymes (top panel). The scaffoldin assembly system allows the construction of monovalent, bivalent, trivalent, tetravalent and pentavalent scaffoldins. The scaffoldin tile repository allows the construction of 43,833 scaffoldins (middle panel). The combination of both assembly systems allows us to consider 6 × 10^17^ different GM-degrading designer cellulosomes (bottom panel). The number of proteins that can be constructed was calculated using the number of tiles present in the tile repository and shown in Tables 1 and 2

In Fig. 1, the full combinatorial power of VersaTile is displayed. With this repository, 3578 different docking enzymes and 43,883 scaffoldins can be constructed. With a total of 3578 different docking enzymes and 2 CBMs that can be located at 5 different positions, a stunning number of 6 × 10^17^ (= (3578^4^ × 5 × 2) + (3578^5^)) multivalent designer cellulosomes with one CBM can be considered.

A total of 151 modular proteins (79 mono- and bicatalytic docking enzymes and 72 scaffoldins) were constructed in this study. To facilitate the interpretation of the results, we have numbered these constructs and given each of them a specific icon. A full list of the constructed docking enzymes and scaffoldins can be found in Additional file 1: Tables S7 and S8, respectively. We have constructed monocatalytic docking enzymes with C- and N-terminal dockerins. For some of these constructs, the enzymatically active module was directly fused to the dockerin. In other cases, these two modules were separated by a selected linker sequence. The bicatalytic docking enzymes, composed of two enzymatically active modules and one dockerin, carry the latter module C- or N-terminally or in between the two selected enzyme modules. To allow immobilised metal affinity chromatography (IMAC)-purification, an N- or C-terminal His-tag was appended to all docking enzymes. When the tag was added as a tile, the assembly was performed in destination vector pVTD2. When all positions were occupied by dockerin, linker and/or enzyme tiles, the assembly was performed in destination vectors pVTD1 or pVTD3, in which the His-tag is encoded on the vector backbone. While the former allows the fusion of an N-terminal His-tag, the latter ensures the fusion of a C-terminal His-tag (Additional file 1: Fig. S6). The scaffoldins all contained one to five cohesins and one CBM. Scaffoldins were assembled in pVTD2, pVTD3, pVTD5, pVTD13 or pVTD17. When the His-tag was added as a tile, pVTD2 was used for assembly. pVTD5 and pVTD10 allowed the fusion of an N-terminal StrepII-tag and an N-terminal His- and GST-tag, respectively. Use of pVTD13 ensured the fusion of an N-terminal GST-tag and a C-terminal His-tag and assembly in pVTD17 resulted in an N-terminal GST-tag and CBM and a C-terminal His-tag (Additional file 1: Fig. S6). The addition of the CBM, GST- or StrepII-tag allows us to use these tags for purification purposes.

### Parameters influencing expression level, stability and activity of docking enzymes

Based on initial exploratory analyses of randomly designed docking enzymes, a total of 79 docking enzymes were constructed (Additional file 1: Table S7). Docking enzymes were expressed and purified by IMAC. Subsequently, their activities were tested based on the release of reducing ends using the 3,5-dinitrosalicylic acid (DNS), and/or a para-nitrophenol (pNP) assay. This setup allowed us to perform systematic pairwise comparisons to navigate through the multiparametric landscape that determines the eventual outcome. Below, we discuss the main parameters influencing docking enzyme expression level, stability and enzymatic activity, supported by a selection of data obtained throughout this work.

### Delineation of tiles

Carbohydrate active enzymes (CAZymes) are often multimodular in nature, comprising enzymatically active modules, CBMs and intervening linkers. Usually, only the enzymatically active module is incorporated in the synthetic docking enzyme. Therefore, careful delineation of the tile is crucial to allow efficient conversion to the cellulosomal mode. Delineation is often complicated due to the absence of an available crystal structure. In addition, it cannot be excluded that a difference of a single amino acid affects the outcome either at the expression, folding or activity level. Furthermore, one of the key choices is to include the natural linker in a tile or not. The most optimal delineation is thus an empirical process, but the throughput of the VersaTile technique allows to explore this aspect more extensively. Generally, we have delineated tiles based on domain (HMMER [25]) and secondary structure (Phyre2 [26]) analyses, taking into account that predicted secondary structure elements should not be interrupted.

**Fig. 2:**
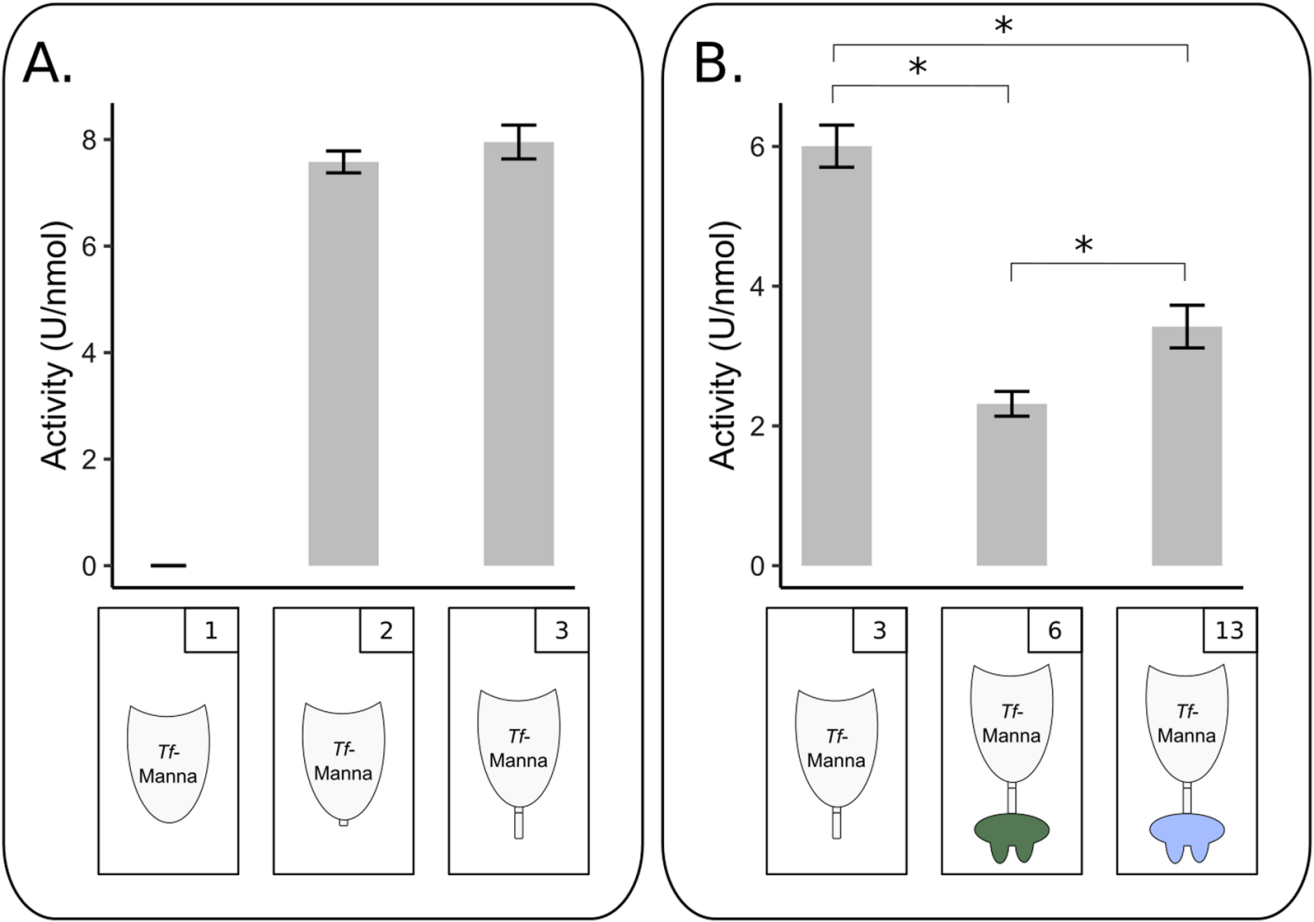
Influence of tile delineation and dockerin/enzyme combination on the activity of docking enzymes. **A.** Influence of tile delineation on the activity of *T. fusca* mannanase enzyme. The activity of *Tf*-Manna-S (construct nr. 1), *Tf*-Manna (construct nr. 2) and *Tf*-Manna-Li (construct nr. 3) is shown. Construct nr. 1 was inactive. Construct nr. 2 and 3 had the same activity. **B.** Influence of dockerin addition and dockerin type on the activity of *T. fusca* mannanase docking enzymes. The activity of *Tf*-Manna-Li (construct nr. 3), *Tf*-Manna-Li_Doc-*Ct*II (construct nr. 6) and *Tf*-Manna-Li_Doc-*Ac* (construct nr. 13) are shown. Reaction mixtures were composed of 1 mL 0.5% GM and 20 pmol enzyme. Enzymatic activity was determined quantitatively by measuring the released reducing sugars with the DNS method. Slopes were converted to enzymatic activity (U/nmol). Each bar represents the mean ± SD of three replicates. *p < 0.05, Student’s *t*-test

This empirical optimisation process was performed for the natural *T. fusca* mannanase enzyme. An unstructured protein sequence intervenes the N-terminal GH5 enzymatic module and the C-terminal family 2 CBM. The C-terminal part of this unstructured region is a proline-rich sequence, which is typically associated with a linker function. Four different tile variants of this enzyme were prepared, increasingly truncating the C-terminal CBM and linker (Additional file 1: Table S6). First, CBM truncation was done. In addition, we performed a stepwise truncation of the linker for the proline-rich region and the remaining linker moiety, respectively. We evaluated the effect of truncating the *T. fusca* mannanase before fusing it to a dockerin module. Since a negative influence of multiple CBMs in a designer cellulosome has been reported [27, 28], we started with testing the tile variants that excluded the CBM sequence. The shortest variant (construct nr. 1) was inactive. There was no significant difference in activity between the second (construct nr. 2) and third variant (construct nr. 3) (Fig. 2A), indicating that the added linker does not disturb the activity. This pairwise comparison demonstrates the major impact of the delineation parameter.

### Dockerin—enzyme combination

During this work, seven different dockerins were used to construct docking enzymes. Although there are sequence similarities between dockerins from the same type, switching the dockerin module will result in the construction of a substantially different docking enzyme with difficult-to-predict characteristics. In some cases, enzymes can only be expressed when fused to a specific dockerin. In other cases, the selected dockerin drastically influences the enzymatic activity of the docking enzymes.

For example, four different dockerins were C-terminally fused to *Cj*-Aga (Doc-*Ct*I: construct nr. 56, Doc-*Ct*II: construct nr. 57, Doc-*Cc*: construct nr. 58, Doc-*Rf*: construct nr. 59). Only constructs nr. 56 and 58 (incorporating Doc-*Ct*I or Doc-*Cc*) could be expressed. In the case of *Tf*-Manna-Li, being the *T. fusca* mannanase including its natural linker, we evaluated several direct C-terminal fusions with different dockerins. The fusion protein composed of *Tf*-Manna-Li and Doc-*Cc* (construct nr. 7) could not be expressed. A fusion of the same enzymatically active module to Doc-*Ct*II (construct nr. 6) resulted in an activity loss of 61% compared to the natural enzyme without a dockerin. A fusion to Doc-*Ac* (construct nr. 13) resulted in an activity loss of 43% (Fig. 2B).

### Docking enzyme architecture

Dockerins can be fused to the selected enzymes N- or C-terminally and this arrangement can greatly influence the overall efficiency of the constructed docking enzymes. When a selected enzyme is multimodular in nature, the natural CBM module is often replaced with the dockerin, as such preserving the natural modular order of the enzyme. However, several enzymes are composed of a single module. In these cases, it is difficult to predict the optimal position of the dockerin and this needs to be tested empirically. Although dockerins are usually positioned C-terminally in their native proteins, they have been found N-terminally [29] and internally [30] between two enzymatically active modules as well. Moreover, dockerins that are positioned C-terminally in their native protein have also been shown to be active when positioned N-terminally in a designer cellulosome [18, 31], leaving the protein engineer with a multitude of design choices when constructing a novel docking enzyme.

**Fig. 3:**
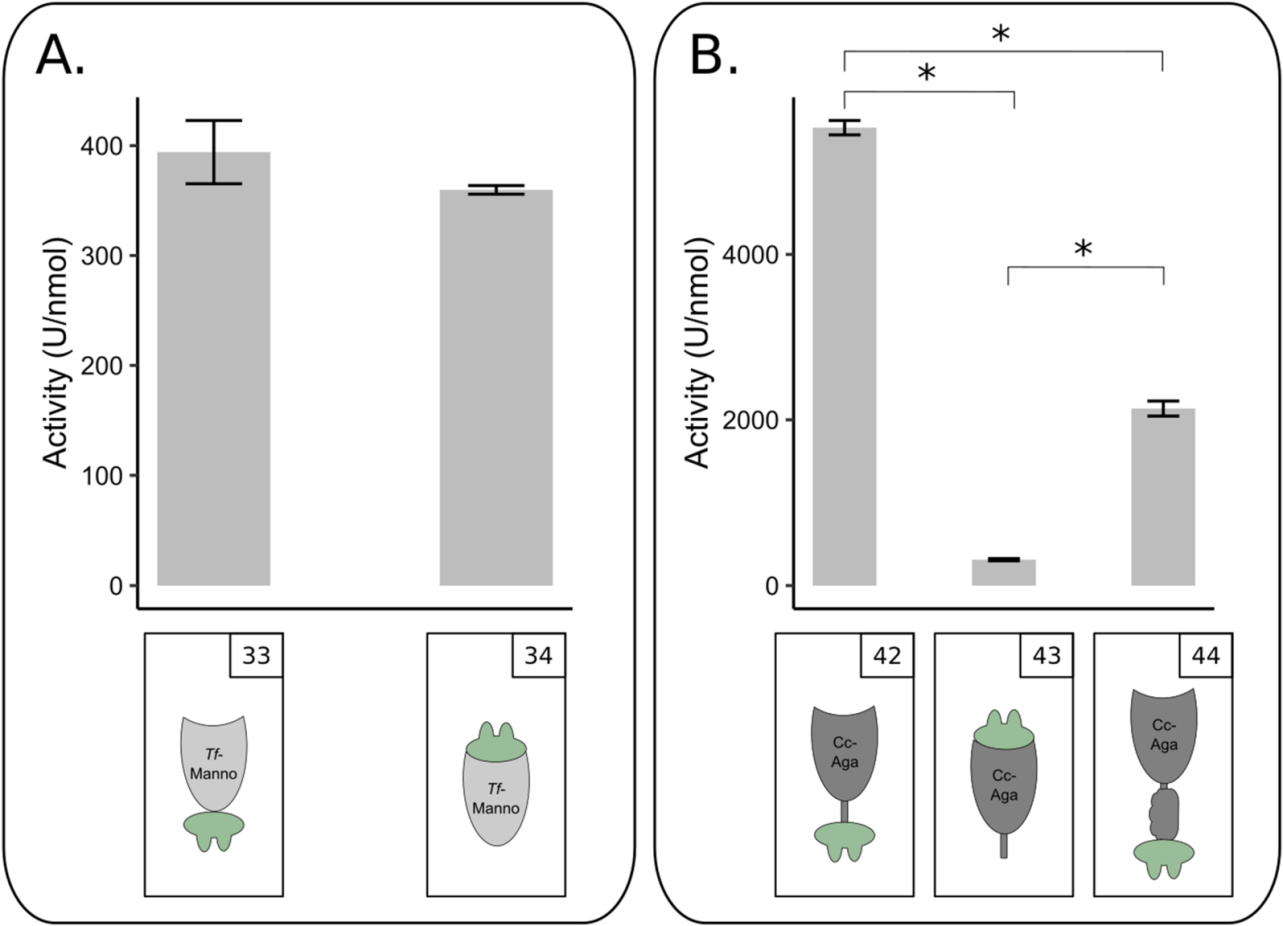
Influence of docking enzyme architecture on the activity of docking enzymes. **A.** Influence of dockerin position on the activity of *T. fusca* mannosidase docking enzymes. The activity of *Tf*-Manno_Doc-*Ct*I (construct nr. 33) and Doc-*Ct*I_*Tf*-Manno (construct nr. 34) are shown. Reaction mixtures were composed of 1 mL 2.5 mM pNP-β-mannose and 3 pmol enzyme. **B.** Influence of dockerin position and modular arrangement on the activity of the *C. cellulolyticum* galactosidase docking enzymes. The activity of *Cc*-Aga-Li_Doc-*Ct*I (construct nr. 42), Doc-*Ct*I_*Cc*-Aga-Li (construct nr. 43) and *Cc*-Aga-CBM_Doc-*Ct*I (construct nr. 44) are shown. Reaction mixtures were composed of 1 mL 2.5 mM pNP-α-galactose and 3 pmol enzyme. Enzymatic activity was determined quantitatively by measuring the released pNP. Slopes were converted to enzymatic activity (U/nmol). Each bar represents the mean ± SD of three replicates. *p < 0.05, Student’s *t*-test

The influence of dockerin position on the activity of docking enzymes was analysed for *Tf*-Manna-Li, *Tf*-Manno and *Cc*-Aga. The *C. thermocellum* type I dockerin was used for this analysis. The purification yield of the mannanase docking enzyme with N-terminal dockerin (construct nr. 5) was drastically lower than the purification yield of the docking enzyme with C-terminal dockerin (construct nr. 4) (Additional file 1: Fig. S7). Additionally, the N-terminal variant was inactive. This indicates that the C-terminal CBM of the *T. fusca* mannase is best substituted by a C-terminal dockerin, thereby maintaining the natural linker function. The natural mannosidase does not contain a CBM or other modules. In contrast to *Tf*-Manna, purification yields were higher with the dockerin positioned at the N-terminus of *Tf*-Manno (constructs nr. 33 and 34) (Additional file 1: Fig. S8). The activity of the N-terminal and C-terminal variants on pNP-β-mannose was similar (Fig. 3A). This confirms that dockerins that are in their native protein positioned at the C-terminus can also be positioned at the N-terminal side of enzymes without diminishing the enzymatic activity [18, 31]. In this particular case, the N-terminal dockerin even appears to enhance the docking enzyme expression level.

*Cc*-Aga was converted to the cellulosomal mode in three different ways. The natural enzyme including its C-terminal linker was fused to the *C. thermocellum* dockerin either C- (construct nr. 43) or N-terminally (construct nr. 42). A C-terminal dockerin was also fused to the full enzyme, including its naturally occurring C-terminal CBM (construct nr. 44). When the dockerin was placed N-terminally, a less stable galactosidase, prone to degradation (visible as additional smaller bands on SDS-PAGE), was observed after purification (Additional file 1: Fig. S9). Activity analysis on p-nitrophenyl-α-D-galactopyranoside (pNP-α-galactose) showed that the construct with a C-terminal dockerin, but without CBM had the highest activity (Fig. 3B). The activity of the N-terminal docking enzyme variant had a significantly lower activity than the variant with C-terminal dockerin, indicating once again the importance of preserving the natural modularity of an enzyme when converting it to the cellulosomal mode. In addition, this example also demonstrated again the effect of tile delineation.

### Monocatalytic vs bicatalytic docking enzymes

Some native cellulosomal enzymes are multicatalytic, bearing multiple copies of catalytic modules and a single dockerin in a single polypeptide chain [32]. Some studies on designer cellulosomes have indeed shown promising results using bicatalytic docking enzymes [33, 34]. Their results have indicated that next to the intermolecular synergy achieved by colocalising multiple docking enzymes, intramolecular synergy by designing multicatalytic docking enzymes can also be pursued. Using VersaTile, we quickly constructed a range of sixteen bicatalytic docking enzyme variants (constructs nr. 64-79). Several of the bicatalytic docking enzymes had high purification yields. However, they proved to be unstable as visualised on SDS-PAGE and monocatalytic docking enzymes were therefore selected for incorporation in the final complex.

### Linker inserted in between the enzymatically active module and dockerin

The linker that merges the dockerin with the enzymatically active module determines the distance between the two modules and consequently the overall architecture of the docking enzyme. The selected linker type and length can have a significant effect on the activity of the docking enzyme and as such of the whole complex. In general, little research has been performed on the role of docking enzyme linkers. However, in a recent study, Kahn and colleagues (2019) illustrated their importance [8]. With VersaTile, we are now able to quickly construct docking enzyme variants comprising the same enzymatically active module and dockerin module, but a different linker sequence.

**Fig. 4:**
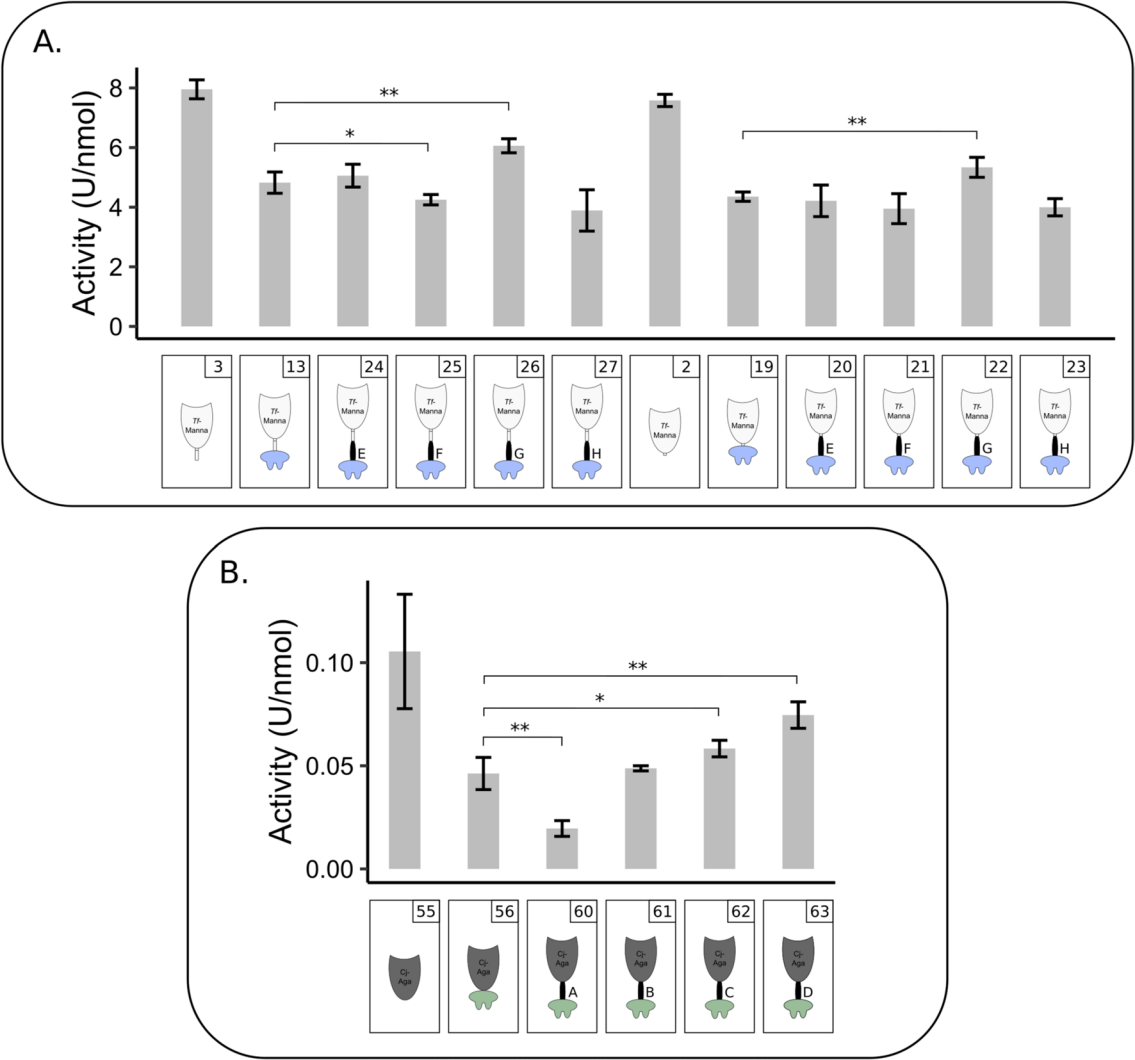
Effect of linker inserted between enzyme and dockerin module. **A.** Effect of different linkers inserted between *T. fusca* mannanase and *A. cellulolyticus* dockerin on the activity of mannanase docking enzymes. The activity of *Tf*-Manna-Li (construct nr. 3), *Tf*-Manna-Li_Doc-*Ac* (construct nr. 13), variants of *Tf*-Manna-Li and Doc-*Ac* with linkers E–H (constructs nr. 24, 25, 26 and 27), *Tf*-Manna (construct nr. 2), *Tf*-Manna_Doc-*Ac* (construct nr. 19) and variants of *Tf*-Manna and Doc-*Ac* with linkers E–H (constructs nr. 20, 21, 22 and 23) are shown. Reaction mixtures were composed of 1 mL 0.5% GM and 10 pmol enzyme. **B.** Effect of different linkers inserted between *C. japonicus* galactosidase and *C. thermocellum* type I dockerin on the activity of galactosidase docking enzymes. The activity of *Cj*-Aga (construct nr. 55), *Cj*-Aga_Doc-*Ct*I (construct nr. 56), variants of *Cj*-Aga and Doc-*Ct*I with linkers A-D (constructs nr. 60, 61, 62 and 63) are shown. Reaction mixtures were composed of 1 mL 0.5% GM and 48 pmol enzyme. Enzymatic activity was determined quantitatively by measuring the released reducing sugars with the DNS method. All slopes were converted to enzymatic activity (U/nmol). Each bar represents the mean ± SD of three replicates. Docking enzymes with linker were compared to the docking enzyme without a linker. *p < 0.1, Student’s *t*-test; **p < 0.05, Student’s *t*-test

The effect of different linkers inserted between the enzyme and the dockerin was analysed for *Tf*-Manna (Fig. 4A) and *Cj*-Aga (Fig. 4B). For the mannanase, the best performing dockerin (Doc-*Ac*) (Fig. 2B) was combined with either *Tf*-Manna-Li or *Tf*-Manna using four different linkers (Li-E: constructs nr. 20 and 24, Li-F: constructs nr. 21 and 25, Li-G: constructs nr. 22 and 26, or Li-H: constructs nr. 23 and 27). When *Tf*-Manna and *Tf*-Manna-Li were directly fused to the dockerin (constructs nr. 19 and nr. 13, respectively), an activity drop of approximately 40% was detected compared to the unfused proteins. In the case of Li-G (constructs nr. 22 and nr. 26), the loss of enzymatic activity due to the dockerin fusion could be partially compensated, limiting the activity loss to 30% for *Tf*-Manna and 24% for *Tf*-Manna-Li, indicating that for this specific enzyme-dockerin combination, a long, rigid linker is the optimal choice. *Cj*-Aga_Doc-*Ct*I (construct nr. 56) had low expression levels. The introduction of different linkers (Li-A: construct nr. 60, Li-B: construct nr. 61, Li-C: construct nr. 62, Li-D: construct nr. 63) improved purification yields. Again, activity analysis revealed that a direct dockerin fusion (construct nr. 56) significantly decreased the enzyme activity (56%). This was compensated to some extent by the introduction of an appropriate linker. For this specific dockerin-enzyme combination, a short proline-rich linker (Li-D: construct nr. 63) proved to be the optimal choice, resulting in a limited activity loss of 30%. Use of another linker, such as linker A (construct nr. 60), further decreased enzymatic activity to approximately 80% loss compared to the natural variant.

### Selected substrate

**Fig. 5:**
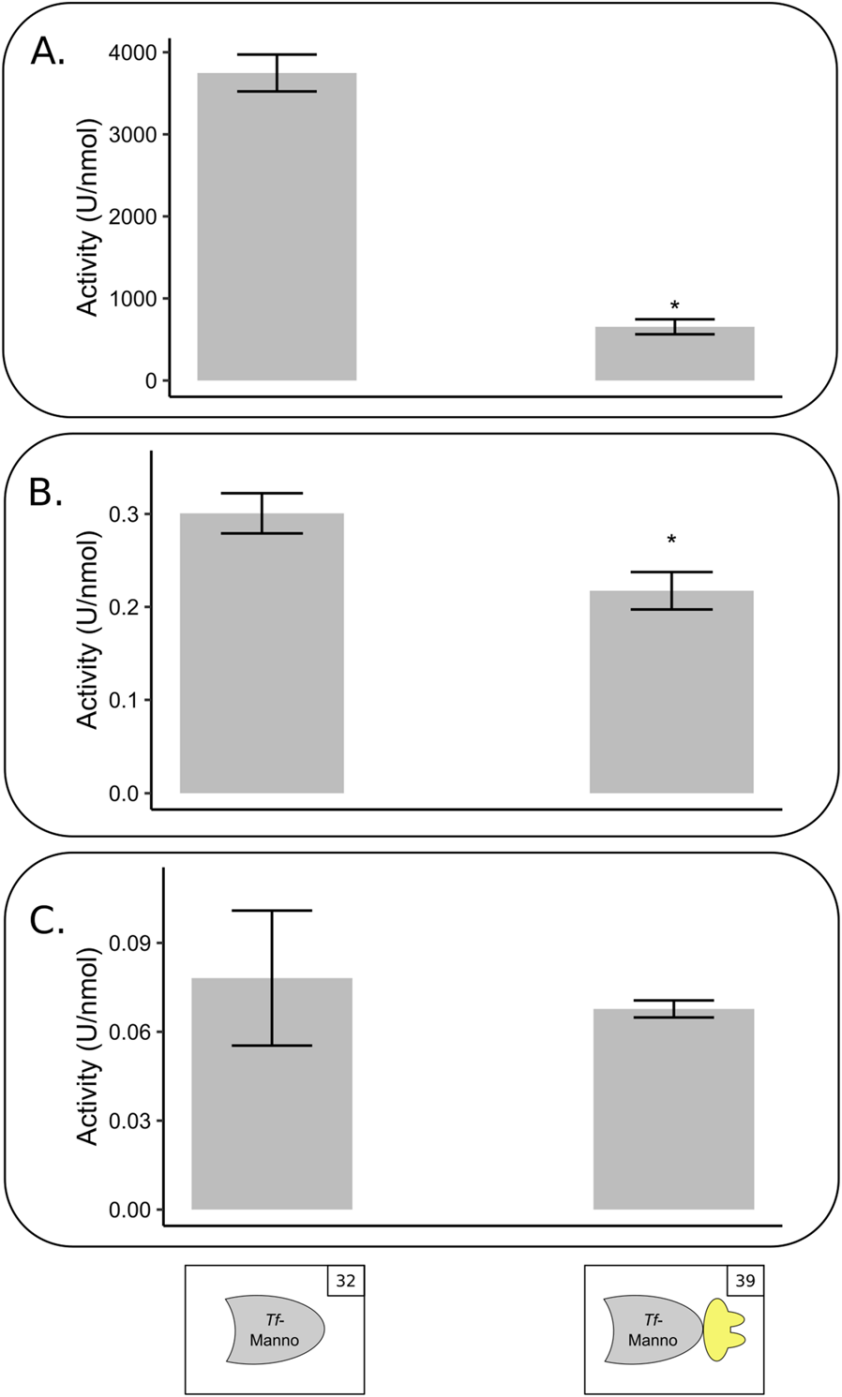
Effect of dockerin addition on the activity of *T. fusca* mannosidase on different substrates. The activity levels of *Tf*-Manno (construct nr. 32) and *Tf*-Manno_Doc-*Bc* (construct nr. 39) are shown. **A**. Activity analysis on pNP-β-mannose. Reaction mixtures were composed of 1 mL 2.5 mM pNP-β-mannose and 2 pmol enzyme. Enzymatic activity was determined quantitatively by measuring the released pNP. **B.** Activity analysis on mannan. Reaction mixtures were composed of 1 mL 0.1% mannan and 48 pmol enzyme. Enzymatic activity was determined quantitatively by measuring the released reducing sugars with the DNS method. **C.** Activity analysis on GM. Reaction mixtures were composed of 1 mL 0.5% GM and 96 pmol enzyme. Enzymatic activity was determined quantitatively by measuring the released reducing sugars with the DNS method. All slopes were converted to enzymatic activity (U/nmol). Each bar represents the mean ± SD of three replicates. *p < 0.05, Student’s *t* test

Notably, fusion of a dockerin module had a different effect on the enzyme activity depending on the substrate used. The negative effect of the dockerin fusion appears to become less prominent when more complex substrates are used. When comparing the activity of the natural mannosidase enzyme (construct nr. 32) to the docking enzyme (construct nr. 39) on the simple substrate, pNP-β-mannose, it is clear that dockerin fusion caused a severe loss of activity (82%). However, when using the more complex substrate, mannan, the activity loss is limited to 28%. For the most complex substrate, GM, no significant loss in activity was observed (Fig. 5).

### Construction, purification and analysis of a trivalent designer cellulosome

To set up a trivalent designer cellulosome able to degrade GM, the multiparametric landscape had to be reduced to three complementary docking enzymes. The major and essential criterion was to select three enzymes, each fused to a different dockerin. This renders the optimisation process of the different docking enzymes interdependent, since the selection of one docking enzyme excludes the use of its dockerin for the other docking enzymes.

Out of all docking enzyme types, the construction of an active α-galactosidase docking enzyme proved to be the most challenging. We were unable to convert the *B. adolescentis* α-galactosidase to the cellulosomal mode. The designed constructs were either enzymatically inactive or could not be expressed. Several of the *C. cellulolyticum* α-galactosidase docking enzymes were active on pNP-α-galactose but had minimal activity on the more complex substrate, GM. Even when both backbone-acting enzymes were added to the reaction mixture, galactosidase activity remained minimal. We note that this might be due to the selected reaction temperature of 50 °C, which is suboptimal for this specific enzyme. Whereas the enzyme was briefly incubated at this temperature during the activity assay using pNP-gal as the substrate, a much longer incubation time was used when galactomannan served as the substrate. This could have resulted in (partial) denaturation of the enzyme, resulting in a lack of detected activity. This left the *C. japonicus* α-galactosidase as the only suitable enzyme to be fitted in the envisioned designer cellulosome. With only the *Ct*I- and *Cc*-dockerin fusions of this enzyme expressing well, *Cj-*Aga was particularly restrictive in the selection of its dockerin. Since the *Cj*-Aga_Doc-*Ct*I fusion protein showed low expression levels, this fusion was further engineered by linker optimisation, resulting in *Cj*-Aga_Li-D_Doc-*Ct*I (construct nr. 63) (Fig. 4B) as the first selected docking enzyme. *Tf*-Manno_Doc-*Bc* (construct nr. 39), which bears Doc-*Bc* could easily be purified, was stable with no degradation or multimerisation and showed clear activity (Fig. 5). Therefore, *Tf*-Manno_Doc-*Bc* was not further optimised. The delineation experiment indicated *Tf*-Manna-Li as the best delineation. Pairwise comparisons and linker optimisation finally yielded *Tf*-Manna-Li_Li-G_Doc-*Ac* (construct nr. 26) (Fig. 4A).

**Fig. 6:**
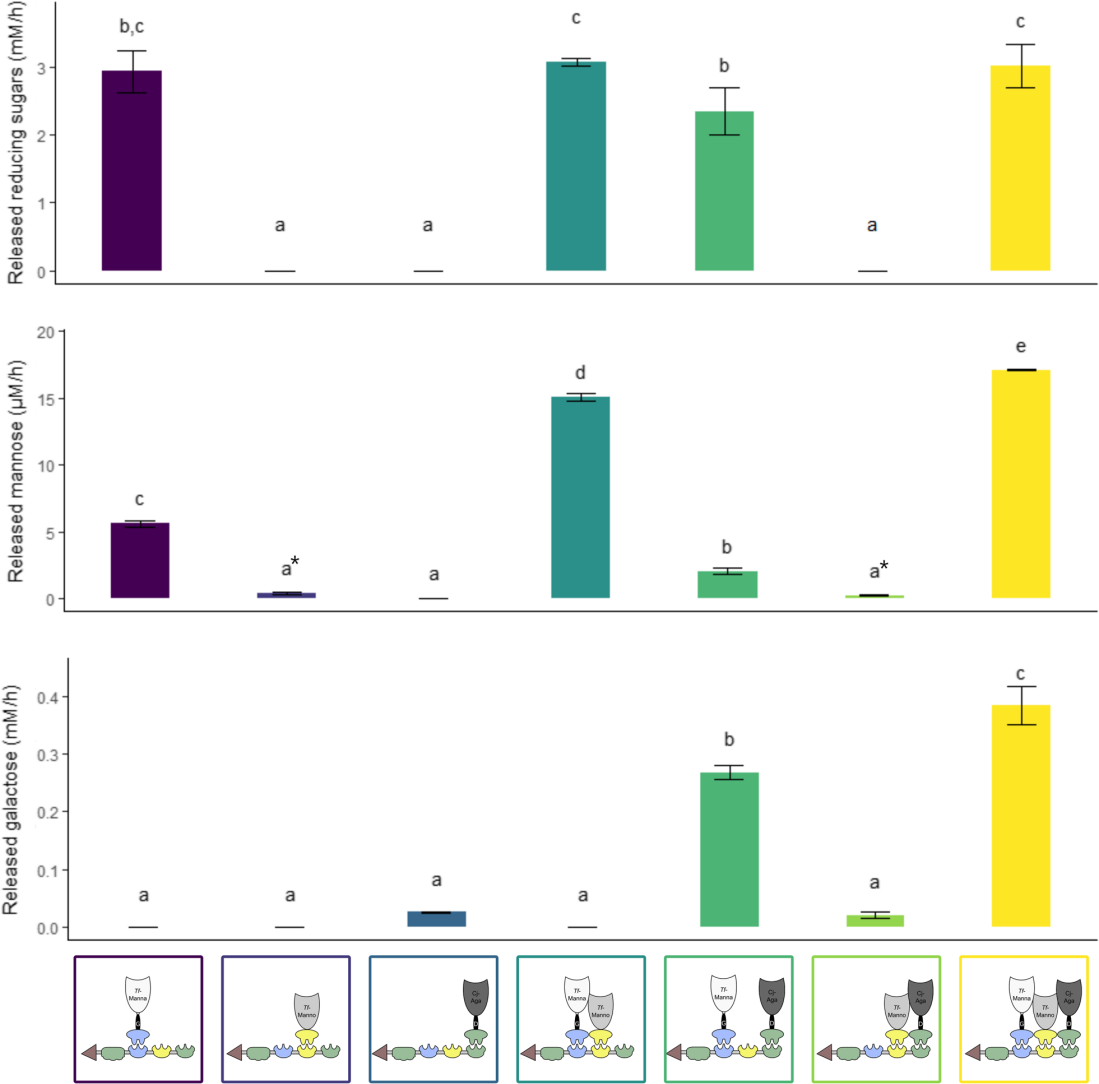
Released reducing sugars, mannose and galactose when different docking enzymes are incorporated in the complex. The activity of complexes composed of *Ct*-CBM3_Coh-*Ac*_Coh-*Bc*_Coh-*Ct*I (construct nr. 135), *Tf*-Manna_Li-G_Doc-*Ac* (construct nr. 26, white), *Tf*-Manno_Doc-*Bc* (construct nr. 39, light grey) and/or *Cj*-Aga_Li-D_Doc-*Ct*I (construct nr. 63, dark grey) was tested. Reaction mixtures were composed of 1 mL 0.5% GM and 10 pmol designer cellulosome. Enzymatic activity was determined quantitatively by measuring the released reducing sugars, mannose and galactose over time. An ANOVA followed by a post hoc Tukey test revealed a significant variation among the tested complexes. *Detected below limit of detection

A scaffoldin composed of cohesins Coh-*Ac*, Coh-*Bc* and Coh-*Ct*I was constructed (construct nr. 135). This scaffoldin was also equipped with the His-tag, to allow IMAC-purification, the *C. thermocellum* CBM3a, to allow binding to cellulose, and a GST-tag to allow pull-down of the multi-enzyme complex. The designer cellulosome was produced by co-incubation of the scaffoldin with the respective docking enzymes. Subsequently, a GST pull-down was performed. SDS-PAGE analysis of the eluted fractions revealed that each docking enzyme could interact with the scaffoldin (Additional file 1: Fig. S10 and S11). Additionally, the pull-down assay allows the isolation of pure complexes that can be subjected to activity analysis. Test reactions consisted of a 0.5% GM solution, supplemented with 10 pmol of designer cellulosome. During the first hour of the assay, the release of reducing sugars was measured. After 2, 4, 6 and 8 h, samples were taken to determine the release of the specific sugar monomers mannose and galactose. Figure 6 shows the results of the activity assay when the selected mannanase, mannosidase and/or galactosidase docking enzymes were incorporated in the complex.

The results indicate that the GM backbone is only degraded to MOS when mannanase is incorporated in the complex. When mannosidase and/or galactosidase are also included, we do not observe a significant increase in released reducing ends. In this case, mannose and/or galactose are present in the reaction mixture at the micromolar range. They are thus not detected when measuring the released reducing sugars. This signal is overwhelmed by the release of MOS and is therefore only linked to mannanase activity. When only mannanase is included in the complex, mannose is released at a rate of 5.6 µM/h. A complex only including mannosidase released no mannose. Combining these two enzymes results in a mannose release of 15.1 µM/h. Upon addition of galactosidase, a maximum rate of mannose release of 17.1 µM/h is achieved. The rate of mannose release is notably lower than the rate of reducing sugar release (0.57%), indicating that the majority of degradation products are MOS.

When only galactosidase is incorporated in the complex, galactose monomers are released at a rate of 26 µM/h. Upon addition of mannanase, galactose is released ten times faster (267 µM/h). Galactosidase benefits from mannanase addition because mannanase releases MOS, which are the preferred substrate for galactosidase. Upon incorporation of mannosidase, galactose release is enhanced an additional 1.4-fold, reaching a maximum galactose release rate of 383 µM/h. This suggests an additional synergistic action between the two exo-acting enzymes. Overall, this threefold activity analysis confirms that each of the incorporated enzymes remains active upon incorporation in the complex.

### Optimising scaffoldin architecture results in an increase of released galactose monomers

With the VersaTile approach at our disposal, it is fairly straightforward to construct multiple scaffoldin variants. As such, we were able to quickly analyse different parameters and further optimise the complex.

**Fig. 7:**
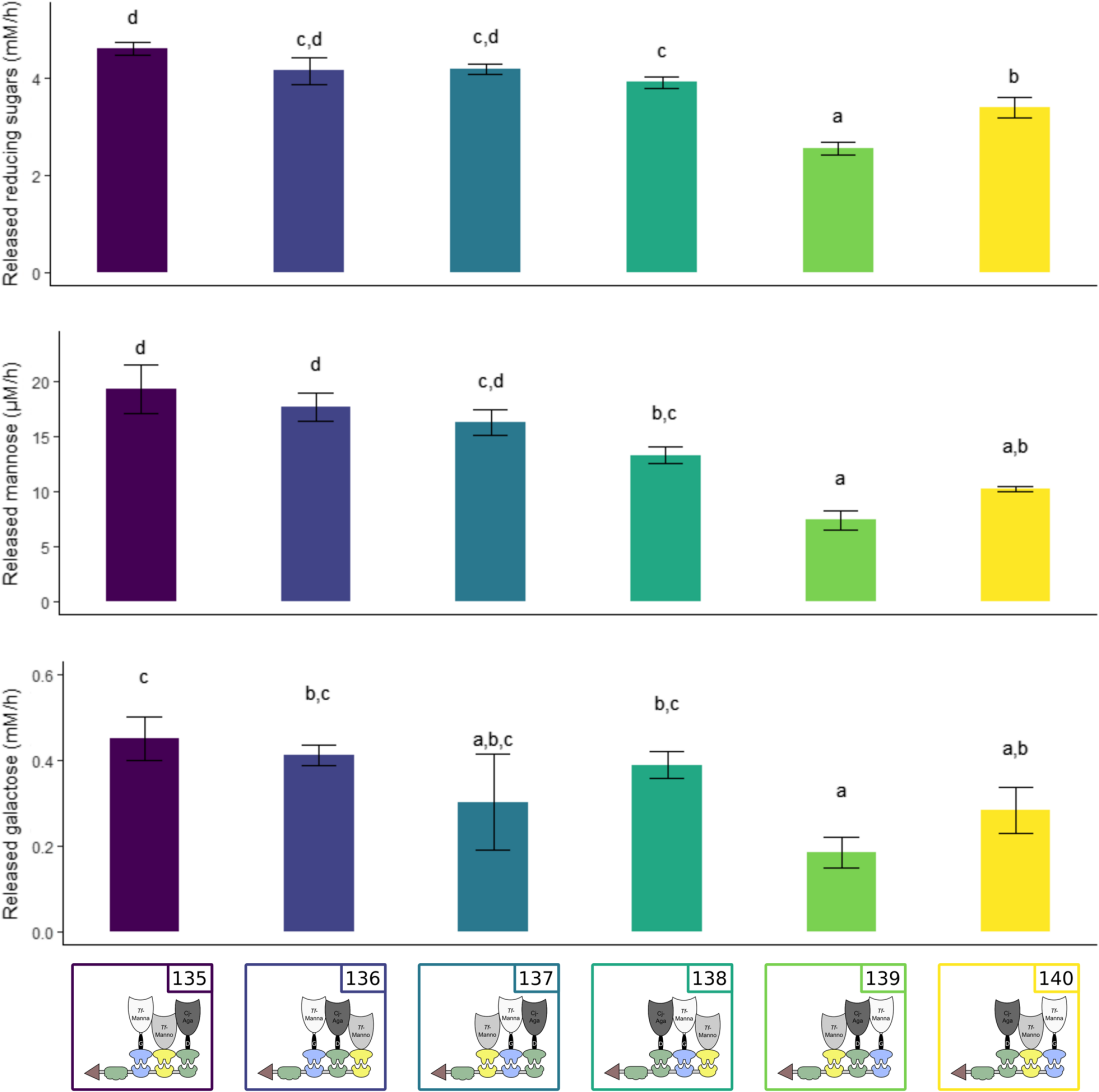
Release of reducing sugars, mannose and galactose monomers for six cellulosomal variants. By employing different scaffoldin variants, enzymes were located at different positions in the complex. All tested complexes contained *Tf*-Manna_Li-G_Doc-*Ac* (construct nr. 26, white), *Tf*-Manno_Doc-*Bc* (construct nr. 39, light grey) and *Cj*-Aga_Li-D_Doc-*Ct*I (construct nr. 63, dark grey) and one of the following scaffoldins: *Ct*-CBM3_Coh-*Ac*_Coh-*Bc*_Coh-*Ct*I (construct nr. 135), *Ct*-CBM3_Coh-*Ac*_Coh-*Ct*I_Coh-*Bc* (construct nr. 136), *Ct*-CBM3_Coh-*Bc*_Coh-*Ac*_Coh-*Ct*I (construct nr. 137), *Ct*-CBM3_Coh-*Ct*I_Coh-*Ac*_Coh-*Bc* (construct nr. 138), *Ct*-CBM3_Coh-*Bc*_Coh-*Ct*I_Coh-*Ac* (construct nr. 139) or *Ct*-CBM3_Coh-*Ct*I_Coh-*Bc*_Coh-*Ac* (construct nr. 140). Reaction mixtures were composed of 1 mL 0.5% GM and 10 pmol designer cellulosome. Enzymatic activity was determined quantitatively by measuring the released reducing sugars, mannose and galactose over time. An ANOVA followed by a post hoc Tukey test revealed a significant variation among the tested complexes

To investigate if the location of the docking enzymes on the scaffoldin influences the activity of the enzymes, six scaffoldin variants were constructed. In each scaffoldin, *Ct*-CBM3 was fixed at the first position and Coh-*Ct*, Coh-*Bc* and Coh-*Ac* were shuffled for positions 2, 3 and 4 (construct nrs. 135–140). Each scaffoldin was combined with the optimised docking enzymes, and complexes were purified by GST pull-down (Additional file 1: Fig. S12 and S13). Test reactions consisted of a 0.5% GM solution, supplemented with 10 pmol of GST-isolated designer cellulosome (Fig. 7).

The results show that each enzyme remained active at every position of the scaffoldin. Release of reducing sugars, galactose and mannose is detected for each of the constructed multi-enzyme complexes. However, not all complexes degrade the GM substrate at the same rate, and a preferred modular arrangement of the scaffoldin, and therefore of incorporated docking enzymes (Mannanase—Mannosidase—Galactosidase) can be detected. Degradation of the GM-backbone to MOS is significantly slower (*p* < 0.05) when mannanase is located at the third position of the scaffoldin (construct nr. 139 and construct nr. 140). The minimal amount of reducing sugars is released when mannosidase is located at the first position and galactosidase at the second (construct nr. 139). We observe the same pattern when analysing the release of mannose and galactose monomers. Once again, this indicates the synergistic action between the backbone-acting mannanase and the other two exo-acting enzymes. When mannanase activity is at its maximum, more easily degradable MOS are present to act as a substrate for the other two enzymes taking part in the complex.

**Fig. 8:**
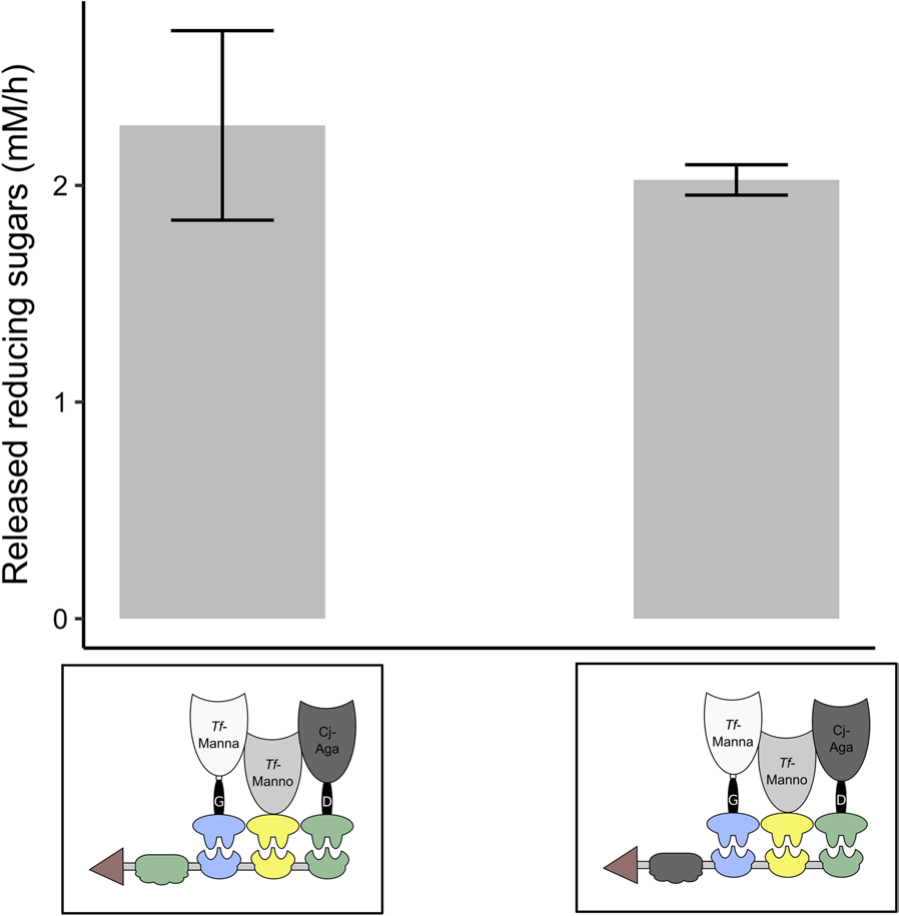
Influence of type of CBM module in the scaffoldin on the activity of the cellulosome complex. The activity of the cellulosome complex composed of *Ct*-CBM3_Coh-*Ac*_Coh-*Bc*_Coh-*Ct*I (construct nr. 135), *Tf*-Manna_Li-G_Doc-*Ac* (construct nr. 26), *Tf*-Manno_Doc-*Bc* (construct nr. 39) and *Cj*-Aga_Li-D_Doc-*Ct*I (construct nr. 63) and the complex composed of *Cj*-CBM35_Coh-*Ac*_Coh-*Bc*_Coh-*Ct*I (construct nr. 134), *Tf*-Manna_Li-G_Doc-*Ac* (construct nr. 26), *Tf*-Manno_Doc-*Bc* (construct nr. 39) and *Cj*-Aga_Li-D_Doc-*Ct*I (construct nr. 63) was tested. Reaction mixtures were composed of 1 mL 0.5% GM and 10 pmol designer cellulosome. Enzymatic activity was determined quantitatively by measuring the released reducing sugars with the DNS method. Each bar represents the mean ± SD of three independent experiments. The Student’s *t*-test revealed that there was no significant difference in activity between the two types of designer cellulosomes (*p* = 0.381)

In all previously described scaffoldins, we incorporated the cellulose-specific *C. thermocellum* CipA CBM3 module. When using cellulosomes to degrade complex lignocellulosic material, this CBM may interact with cellulose while allowing the attached enzymes to degrade surrounding GM, as such releasing the valuable cellulose for further degradation. However, one might also be interested in directing the complex straight to the (galacto)mannan substrate. In this case, replacing the CBM with a mannan-binding module (MBM) can prove useful. We thus constructed additional scaffoldins in which *Ct*-CBM3 was replaced with a mannan-specific CBM originating from *C. japonicus, Cj*-CBM35 (constructs nr. 141–146). The cellulosome incorporating *Cj*-CBM35 essentially released the same amount of reducing sugars from pure GM (Fig. 8, *p* = 0.381) as the one including *Ct*-CBM3.

**Fig. 9:**
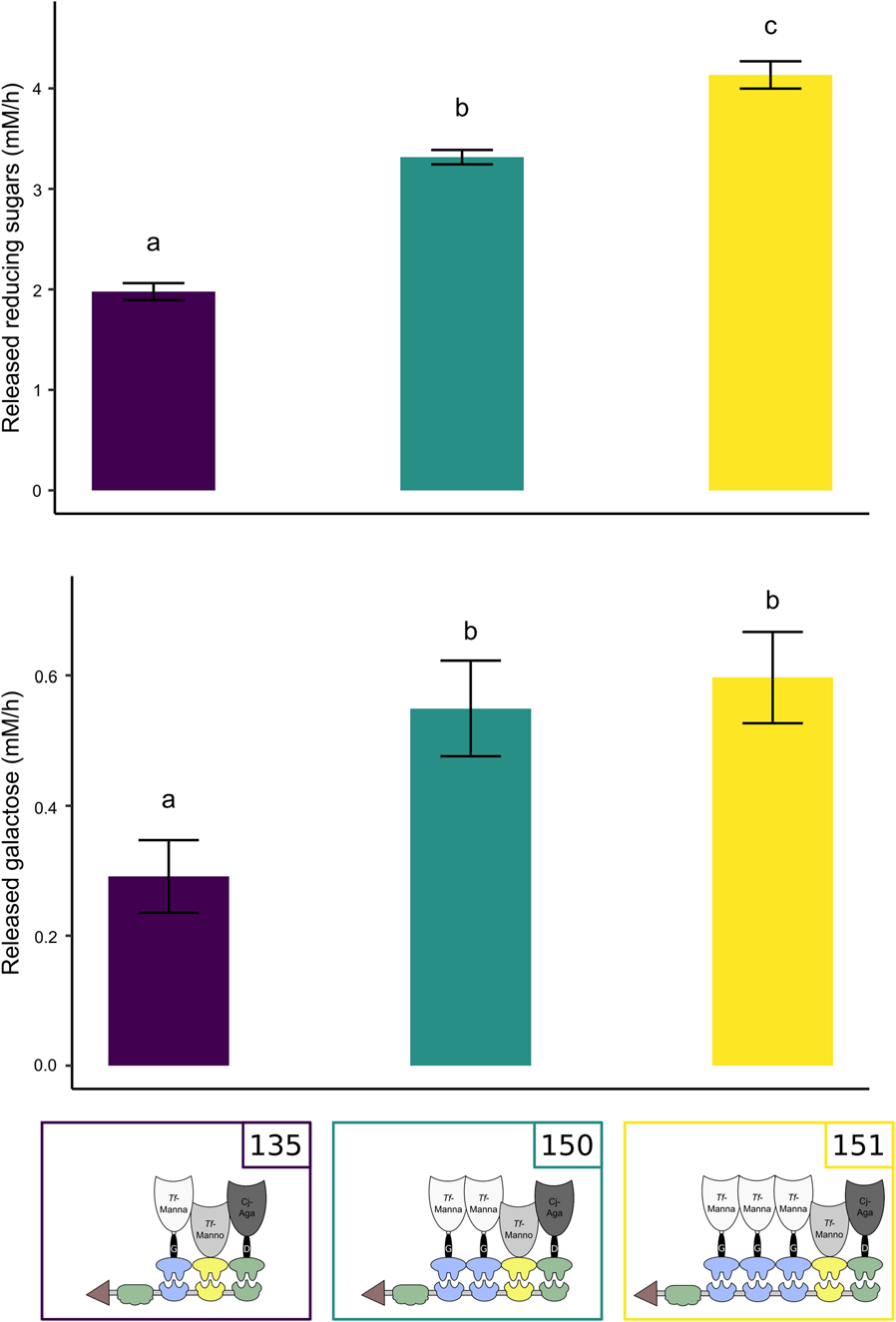
Released reducing ends and galactose monomers upon addition of a complex with multiple mannanase copies. All tested complexes contained *Tf*-Manna_Li-G_Doc-*Ac* (construct nr. 26), *Tf*-Manno_Doc-*Bc* (construct nr. 39) and *Cj*-Aga_Li-D_Doc-*Ct*I (construct nr. 63) and one of the following scaffoldins: *Ct*-CBM3_Coh-*Ac*_Coh-*Bc*_Coh-*Ct*I (construct nr. 135), *Ct*-CBM3_Coh-*Ac*_Coh-*Ac*_Coh-*Bc*_Coh-*Ct*I (construct nr. 150), *Ct*-CBM3_Coh-*Ac*_Coh-*Ac*_Coh-*Ac*_Coh-*Bc*_Coh-*Ct*I (construct nr. 151). Reaction mixtures were composed of 1 mL 0.5% GM and 10 pmol designer cellulosome. Enzymatic activity was determined quantitatively by measuring the released reducing sugars and galactose over time. An ANOVA followed by a post hoc Tukey test revealed a significant variation among the tested complexes

During GM degradation, the backbone-acting mannanase releases MOS. Subsequently, galactose side chains can be removed from MOS by galactosidase. In an attempt to accelerate galactose release, multiple copies of the galactosidase docking enzyme were incorporated in the complex (scaffoldin constructs nr. 147 and 149). However, no additional release of galactose was observed using this approach (data not shown). Conversely, the release of galactose monomers (and reducing ends from MOS) could be significantly enhanced by the incorporation of an additional copy of the backbone-acting mannanase docking enzyme (construct nr. 150) (GST pull-down: Additional file 1: Fig. S14) (Fig. 9).

These results suggest that when using the original complex (incorporating one copy of each enzyme), galactose release is limited by the lack of available substrate. The addition of multiple mannanase copies allows the acceleration of the release of MOS which subsequently serve as a more accessible substrate for galactosidase. The incorporation of three mannanases further accelerated the release of reducing sugars, but did not have a significant impact on the release of galactose monomers. This indicates that two mannanase copies are capable of releasing an excess of MOS, as such fully saturating the present galactosidases and allowing this enzyme to release galactose at its maximal rate.

## Discussion

The goal of this work was to expand the VersaTile platform to allow the fast and efficient construction of designer cellulosomes. We have developed a three-way assembly system to construct docking enzymes and a five-way assembly system to construct scaffoldins. The former allows the construction of (docking) enzymes composed of (1) an enzymatic module alone (control), (2) an enzymatic module and a dockerin, (3) an enzymatic module, a linker and a dockerin or (4) two enzymatic modules and a dockerin. The latter allows the construction of scaffoldins composed of a CBM and/or one, two, three, four or five cohesin modules. We have constructed a tile repository, currently containing 92 tiles encoding non-catalytic cellulosomal building blocks (dockerins, cohesins, CBMs, linkers and tags). The possibilities of the platform were substantially extended by the construction of a range of destination vectors. Upon the addition of a selection of tiles encoding enzymatically active modules, this platform allows the construction of a practically infinite amount of designer cellulosomes. Here, we selected GM to serve as the substrate. With the selected 29 tiles encoding GM-degrading enzymes, a stunning amount of 6 × 10^17^ different designer cellulosome complexes could be envisioned, again underscoring the combinatorial power of VersaTile.

**Fig. 10:**
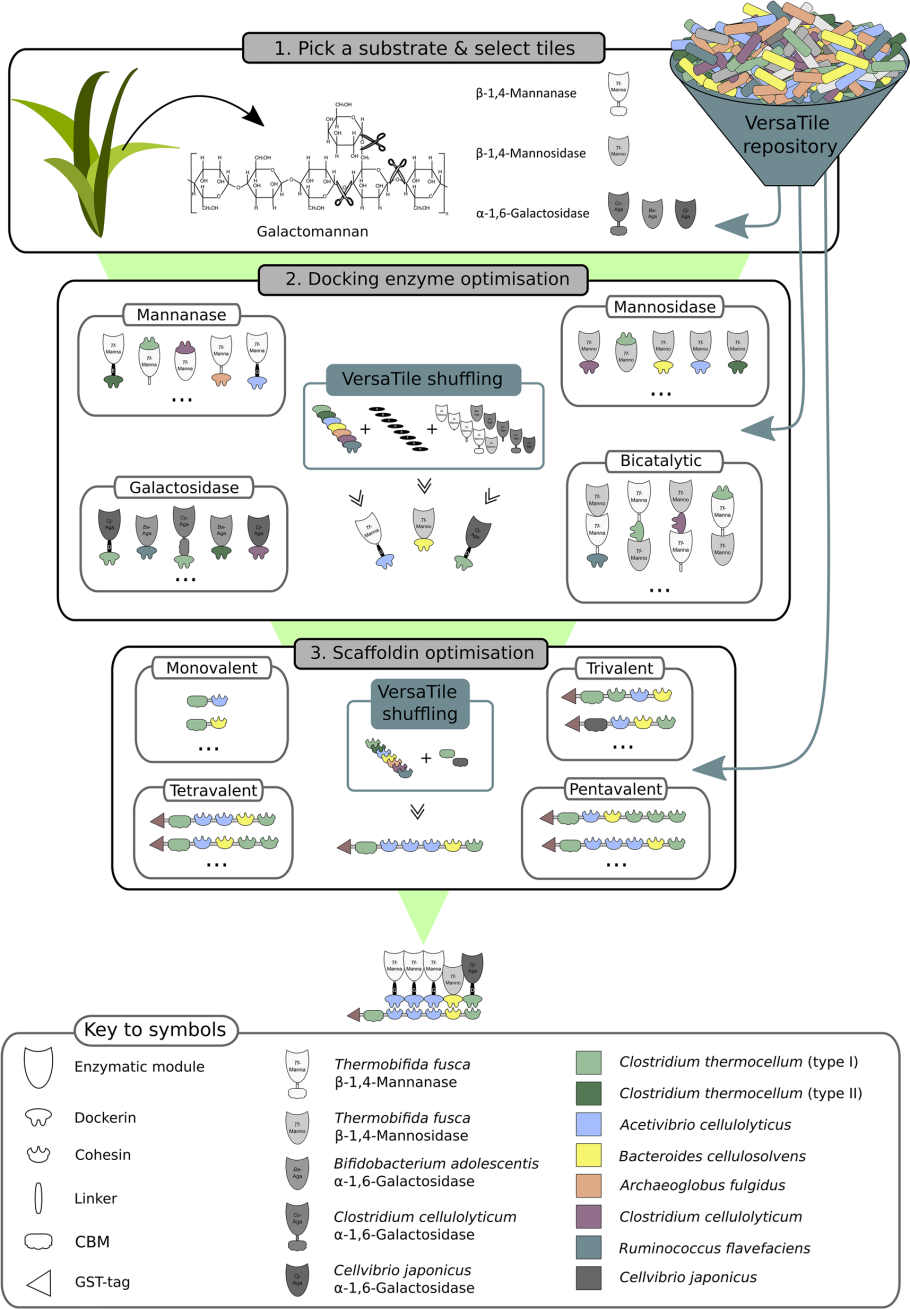
VersaTile platform for the optimisation of designer cellulosomes. Step 1: pick a substrate and select catalytically active tiles from the docking enzyme tile repository. Here, GM was chosen and five GM-degrading enzymes were selected. Step 2: docking enzyme optimisation: VersaTile allows the quick and easy construction of docking enzymes composed of enzyme, dockerin and/or linker tiles. Here, 79 different docking enzymes were designed. Activity assays allowed us to select three optimised docking enzyme variants to incorporate in the final complex. Step 3: scaffoldin optimisation: VersaTile allows the quick and easy construction of scaffoldins composed of five cohesins and/or CBM tiles. Here, 72 different scaffoldins were designed. Activity analysis of complexes composed of these scaffoldins and the selected docking enzymes allowed us to construct an optimised designer cellulosome

As a proof of concept, we aimed to design an optimised designer cellulosome variant able to efficiently degrade the selected GM substrate. Parameters influencing the activity of designer cellulosomes can be either linked to docking enzyme or scaffoldin composition. We therefore used the presented platform to quickly construct a range of different variants for each of these modular proteins, as such allowing the targeted study of specific parameters. First, stepwise optimisations of docking enzymes were facilitated by the technique. Three optimised docking enzyme variants were selected to take part in the designer cellulosome. The number of possible trivalent configurations is huge. Therefore, we again made use of the VersaTile technique to quickly construct multiple scaffoldins, thus facilitating the multiparametric optimisation process. Finally, the platform allowed us to construct an optimised designer cellulosome (Fig. 10).

We have shown that each of the selected docking enzymes was able to perform its function upon incorporation in the complex by quantifying the release of the individual sugars. This is in contrast with many other studies where the performance of the designer cellulosome was monitored through quantification of the release of reducing sugars by the concurrent action of all incorporated enzymes [35].

Our data reveal that the major determinants of docking enzyme efficiency are the module delineation, the selected dockerin, dockerin position and the linker used to fuse the enzymatically active module to the dockerin module. We used HHMMER and Phyre2 to guide us through the delineation parameter. Recently, AlphaFold has been released for computational prediction of three-dimensional protein structures [36]. The implementation of AlphaFold or similar algorithms may drastically facilitate the delineation process. For mannanase, the insertion of a 15 amino acid synthetic linker (linker G: (EAAAK)_3_) between the enzymatic module and the dockerin yielded the optimal docking enzyme. For the galactosidase, a short (6 amino acids) proline-rich linker proved to be the optimal choice. The effect of docking enzyme linker (length) has been analysed before. In the case of Caspi et al. (2009) varying the linker length did not affect enzyme activity [31]. Kahn et al. (2009) showed that for one of their selected enzymes, a long linker variant had an enhanced activity compared to the short linker variant. For another enzyme, no substantial difference in activity was measured [8]. The optimal linker type and length is therefore clearly enzyme-dependent.

Upon analysing the constructed designer cellulosome, we observed a clear synergy between the mannanase and mannosidase docking enzyme and between the mannanase and galactosidase docking enzyme. When mannanase was included in the complex, short MOS are released, which are the preferred substrates for mannosidase and galactosidase. The synergistic action between the endo-acting mannanase and the two exo-acting enzymes has been reported before and was thoroughly reviewed by Malgas et al. in 2015 [37]. Additionally, we detected a cooperative action between the two exo-acting enzymes. It is possible that galactosidase has a preference for oligosaccharides containing galactose-side chains linked to terminal mannose units over oligosaccharides containing galactose substitutions on central mannose units. Studies focusing on synergy between these two enzymes have been limited, but similar observations were made before [38, 39].

Designer cellulosomes are multimodular protein complexes with difficult to predict three-dimensional structures [40–42]. Some parameters influencing the activity of the complexes are clearly linked to the scaffoldin architecture. Stern et al. (2015) constructed a trivalent designer cellulosome incorporating three recombinant *T. fusca* enzymes: processive endoglucanase Cel9A (79 kDa), endoglucanase Cel5A (42 kDa) and exoglucanase Cel48A (83 kDa). Here, the authors observed a preferred modular arrangement of the scaffoldin. The overall activity was enhanced the most when one of the larger enzymes, the processive endoglucanase Cel9A, was not positioned in between the two other enzymatic partners. However, the smallest enzyme, endoglucanase Cel5A (51 kDa), also disfavoured this position. Here, the authors hypothesised that the proximity between Cel9A and Cel5A caused an unproductive competition between the two functionally similar enzymes [7]. Since three functionally distinct enzymes were incorporated in our complex, we presumed that competition by proximity would not be an issue. However, whereas the mannanase and galactosidase used in our study are rather small and similar in size (47 and 54 kDa, respectively), the mannosidase is very large (104 kDa). Nevertheless, positioning the large mannosidase in between the two smaller enzymes did not result in a decreased release of mannose monomers, indicating that docking enzyme size does not necessarily determine the optimal positioning on a scaffoldin. Vazana et al. (2013) obtained similar results. When they constructed designer cellulosomes composed of three recombinant *C. thermocellum* enzymes, exoglucanase Cel48S (82 kDa), endoglucanase Cel8A (52 kDa) and endoglucanase Cel9K (99 kDa), a preferred modular arrangement did not arise [12]. We assume that the inter-cohesin linkers ensure a specific distance between the cohesins. The linkers that connect the cohesins and CBM are also expected to induce important conformational flexibility of the scaffoldin [43]. As such, the constructed scaffoldins likely form three-dimensional structures allowing the interaction of three distinct docking enzymes that do not inhibit each other. In our set-up, each cohesin tile consists of the cohesin sequence, followed by a linker (41, 5 and 6 amino acids for Coh-*Ct*I, Coh-*Bc* and Coh-*Ac*, respectively). The position tags in between the tiles provide an additional linker sequence of 2 amino acids. However, in their 2015 study, Stern and colleagues used long intermodular linkers ranging from 27 to 35 amino acids [7], indicating that proper distancing of the selected cohesins does not always suffice to ensure independent action of the docking enzymes. We hypothesise that enzyme preference for a specific position within the scaffoldin is not necessarily linked to its size but more to its mechanism of action. While not investigated in this study, the flexibility of the VersaTile technique allows to integrate more cohesin variants including different linker lengths. The mere addition of a few tiles suffices to further boost the design space and to elaborate on this parameter as well.

In the field of enzyme cascade colocalisation on scaffolds, multiple copies of bottleneck enzymes are often incorporated in the complex. As such, product accumulation can be circumvented and cascades are optimised [44–46]. In designer cellulosomes, selected enzymes do not act sequentially so this approach has been less explored [47, 48]. However, hemicellulose degradation includes the cooperative action of endo-acting enzymes able to hydrolyse the polysaccharide backbone and exo-acting enzymes able to remove side chains and terminal residues. Since exo-acting enzymes usually have a higher activity on short oligosaccharides, the endo-acting enzyme is responsible for providing their preferred substrates. Optimising docking enzyme ratio can thus also be of interest in the designer cellulosome field. Indeed, in the example discussed in this work, we were able to double the rate of galactose side-chain release by incorporating additional copies of the backbone-hydrolysing docking enzyme. However, integration of additional copies requires longer scaffoldins. We note that the longest scaffoldin used in this study was prone to degradation and rather unstable. As an alternative, adaptor scaffoldins could be used to create stable complexes incorporating multiple enzyme copies [49]. Tsai et al. (2013) displayed a cellulosome complex on the yeast cell surface [50]. Here, the adaptor scaffoldin strategy was used to amplify the number of enzymatic subunits and two copies of two enzymes were incorporated. The same approach was followed by Tian et al. (2019) who incorporated two copies of three different enzymes [51].

Although VersaTile allows high-throughput DNA assembly of designer cellulosomes, the expression and purification of the large number of proteins was a huge effort. The in vitro cellulosomal production and optimisation process thus remains labour intensive. As a consequence, in our current approach we did not exploit the full combinatorial power of VersaTile, which allows the random assembly of millions of modular protein variants. To make designer cellulosomes industrially competitive, future research should focus on finding a way to quickly produce, test and optimise large numbers of these multi-enzyme complexes.

## Conclusion

VersaTile is a DNA-assembly method allowing the fast and efficient construction of designer cellulosomes. With this approach, both docking enzymes and scaffoldins can be produced rapidly. In this work, we have employed VersaTile to produce and optimise the first GM-degrading designer cellulosome. Multiple parameters, linked to both docking enzyme and scaffoldin architecture, influencing the designer cellulosome efficiency were analysed. Enhancing the number of main chain-cleaving docking enzymes incorporated in the scaffoldin allowed us to double the rate of side-chain release. Optimising the docking enzyme ratio may thus become a general practice when optimising future designer cellulosome complexes.

## Materials and methods

### Bacterial strains and growth media

*E. coli* TOP10 cells were used for plasmid storage and *E. coli* BL21(DE3)-RIL cells were used for protein expression. These strains (Agilent Technologies, Belgium) were routinely grown at 37 °C in Lysogeny broth (LB) (1% tryptone, 0.5% yeast extract, 1% NaCl) with shaking (180 rpm) or on LB supplemented with 1.5% of agar. For proper selection of the *E. coli* clones, LB was supplemented with 100 µg/ml of ampicillin, 50 µg/ml kanamycin, 25 µg/ml chloramphenicol (in case of *E. coli* BL21(DE3)-RIL) and/or 5% (w/v) sucrose.

### VersaTile cloning: preparation of tiles

The DNA encoding enzymatically active modules, dockerins, cohesins, CBMs, linkers or tags were converted to tiles using three different approaches. Short tiles (< 100 nucleotides) were constructed by primer cassette hybridisation. Longer tiles (> 100 nt) were either amplified from genomic DNA or ordered by chemical synthesis.

Each primer (Integrated DNA Technologies, Leuven, Belgium) contained the following necessary parts (listed starting from the coding sequence to outwards): a position tag, a BsaI recognition site and another type IIs (SapI, BpiI, BseRI, BsmbI, BtgZI or BfuAI) restriction and recognition site. Primers used to amplify dockerin, cohesin, CBM, linker, tag- and enzyme tiles are listed in Additional file 1: Tables S9–S15. All enzymes were from Thermo Fisher Scientific (Belgium). For cassette hybridisation, the primers were mixed in equal ratios (5 μM) and incubated at 95 °C (5 min), followed by a gradual cool down to 20 °C. Next, the single-stranded overhangs were filled in with Pfu DNA polymerase (2.5 U, 72 °C, 10 min) and 0.2 mM dNTPs. Sequences were amplified with Pfu DNA polymerase (2.5 U) or Phusion DNA polymerase (1 U) following the manufacturer’s instructions with genomic DNA as a template. The size of the amplified DNA fragments was verified through gel electrophoresis, after which the fragments with the correct size were extracted from the gel using the GeneJET Gel Extraction Kit (Thermo Fisher Scientific) following the manufacturer’s guidelines.

Next, the DNA was cloned in a VersaTile entry vector (pVTE) using the following protocol: 100 ng of pVTE, 50 ng of the amplicon/primer cassette/synthetic sequence, 2 μL 10 × T4 DNA ligase buffer, 15 U T4 DNA ligase, 10 U type IIs restriction enzyme (SapI, BpiI, BseRI, BsmbI, BtgZI or BfuAI) in a total volume of 20 µL. Chemical competent *E. coli* TOP10 cells were transformed with the ligation mixture and plated on LB 1.5% agar supplemented with 100 µg/ml ampicillin and 5% (w/v) sucrose. If the coding sequence of a tile contained a BsaI recognition site, it was removed by performing an inverse PCR (iPCR). New tiles were confirmed by Sanger sequencing (LGC genomics, Germany).

### VersaTile shuffling

A VersaTile shuffling reaction was set up using the following protocol: 1 μL of 100 ng/μL pVTD, 1 µL of each tile (50 ng/µL), 1 μL of BsaI (10 U/μL), 3 μL of T4 DNA ligase (1 U/μL) and 2 μL of 10 × T4 DNA ligation buffer in a total reaction volume of 20 μL. The mixture was incubated in the thermocycler using the following program: (1) 2 min at 37 °C, (2) 3 min at 16 °C; steps 1–2 are repeated 50 times, followed by 5 min at 50 °C and finally 5 min at 80 °C. Chemical competent *E. coli* BL21(DE3)-RIL cells (Agilent Technologies, Belgium) were transformed with 5 µL of the ligation mixture and plated on LB 1.5% agar, supplemented with 50 µg/mL kanamycin, 25 µg/mL chloramphenicol and 5% (w/v) sucrose.

### Protein expression and purification

To express and purify the recombinant proteins, *E. coli* BL21(DE3) RIL cells, containing the correct vector, were grown at 37 °C in LB supplemented with 2 mM CaCl_2_. When OD_600_ reached 0.6, isopropyl thio-β-d-galactoside (IPTG) was added to a final concentration of 0.5 mM. After 18 h of incubation at 16 °C and 180 rpm, cells were harvested by centrifugation (5 min, 20,000*g*) and resuspended in lysis buffer (1 × Tris-buffered saline (TBS) (25 mM Tris–HCl; 137 mM NaCl; 3 mM KCl, pH 7,4) supplemented with 2 mM CaCl_2_ and 20–50 mM imidazole). Cells were then disrupted by adding 1 mg/mL lysozyme, performing three freeze–thaw cycles and sonication (Q125, Qsonica). Lysates were cleared (16,000*g*; 20 min) and filtered (Polyvinylidene difluoride (PVDF) membrane; 0.45 μm pore size). His-tagged soluble proteins were purified by IMAC with His GraviTrap columns (GE Healthcare, Belgium) according to the manufacturer’s instructions. Samples were analysed by 12% sodium dodecyl sulphate–polyacrylamide gel electrophoresis (SDS-PAGE), and protein concentrations were determined with the Abs280nm app of the DeNovix DS-11 series spectrophotometer. Extinction coefficients were calculated with the ProtParam tool (ExPASy).

### GST pull-down

Five hundred picomol of IMAC-purified scaffoldin were combined with 600 pmol of each of the selected IMAC-purified docking enzymes. 1 × TBS supplemented with 2 mM CaCl_2_ was added to a final volume of 500 µL. The mixture was incubated at 4 °C for 18 h. Two hundred microliters Glutathione Sepharose® 4B slurry (Sigma Aldrich) were sedimented and washed with 1 × TBS and 2 mM CaCl_2_. Subsequently, the mixture containing the complex was added to the beads and incubated for 30 min at 22 °C in an overhead shaker. The beads were then washed three times with 1 mL 1 × TBS supplemented with 2 mM CaCl_2_. The complex was then eluted using 3 × 100 µL Tris–HCl supplemented with 20 mM reduced glutathione (pH 8). Eluted fractions were analysed on 12% SDS-PAGE, and protein concentrations were determined with the Abs280nm app of the DeNovix DS-11 series spectrophotometer.

### Enzymatic activity assays

To analyse the activity of docking enzymes, three separate reaction mixtures containing the substrate solution and a specific amount of docking enzyme originating from the same IMAC-purification were prepared. Some enzymes were tested immediately after being purified. Others were stored at 4 °C before performing the activity assay. To avoid ambiguous data analysis, all pairwise activity comparisons were performed with proteins purified on the same day.

For mannanase, an assay mixture consisted of 1 ml 0.1% (w/v) mannan or 0.5% (w/v) GM in reaction buffer (0.2 M Sørensen’s phosphate buffer (pH 7.4) or 1 × TBS buffer (pH 7.4)) and 10 or 20 pmol of docking enzyme or cellulosome complex. The reaction was carried out at 50 °C and 400 rpm. When 10 pmol of enzyme was added, samples were taken every 10 min for a total of 90 min. When 20 pmol of enzyme was added, samples were taken every 5 min for the first 20 min and every 10 min for the last 50 min. The 50 µl samples were mixed with 50 µl DNS reagent (1% DNS, 40% Rochelle salt, 0.2% phenol and 0.5% sodium sulphite, all dissolved in 1.5% NaOH and mixed in a 1:1:1:1 ratio) and incubated at 100 °C for 10 min. After cooling down to room temperature, samples were transferred to 96-well microtiter plates. The degree of enzymatic hydrolysis was determined spectrophotometrically by measuring the absorbance at 540 nm. The released reducing sugars were determined against a 0.5–8 mM mannose standard curve.

For mannosidase, the para-nitrophenol (pNP) assay was used to determine the activity on pNP-mannose. An assay mixture consisted of 1 mL 2.5 mM pNP-β-mannose in reaction buffer and 2 or 3 pmol of docking enzyme or cellulosome complex. The reaction was carried out at 50 °C and 300 rpm. When adding 2 pmol of enzyme, samples were taken every 2 min for a total of 20 min. When adding 3 pmol of enzyme, samples were taken every minute for a total of 10 min. Samples of 50 µl were mixed with 50 μL 10% (w/v) Na_2_CO_3_ in 96-well microtiter plates. The released pNPs were determined by measuring the absorbance at 405 nm. A 0–200 µM pNP standard curve was used. To test the activity of the mannosidase on mannan, reaction mixtures composed of 1 mL 0.1% mannan and 48 pmol enzyme were prepared. Samples were taken every 15 min for a total of 150 min. To test the activity on GM, reaction mixtures composed of 1 mL 0.5% GM and 96 pmol enzyme were prepared. Samples were taken every hour for a total of 7 h. Enzymatic activity was determined quantitatively by measuring the released reducing sugars with the DNS method.

For galactosidase, the para-nitrophenol (pNP) assay was used to determine the activity on pNP-galactose. An assay mixture consisted of 1 mL 2.5 mM pNP-α-galactose in reaction buffer and 3 pmol of docking enzyme or cellulosome complex. The reaction was carried out at 50 °C and 300 rpm. Samples were taken every minute for a total of 11 min. Samples of 50 µl were mixed with 50 μL 10% (w/v) Na_2_CO_3_ in 96-well microtiter plates. The released pNPs were determined by measuring the absorbance at 405 nm. A 0–200 µM pNP standard curve was used. To test the activity of galactosidases on GM, reaction mixtures composed of 1 mL 0.5% GM and 48 pmol enzyme were prepared. Samples were taken every 45 min for a total of 5 h. Enzymatic activity was determined quantitatively by measuring the released reducing sugars with the DNS method.

To facilitate the comparison of different docking enzymes, obtained slopes of the regressions were converted to enzymatic activity (U/nmol). For all docking enzymes, 1 U corresponds to the amount of enzyme that releases one µmol of product per minute in the defined reaction conditions.

When constructing complexes, selected proteins were combined and purified by a single GST pull-down. To test the activity of the GST-purified complex, three separate reaction mixtures were prepared by combining 1 mL of 0.5% (w/v) GM in reaction buffer (1 × TBS buffer + 2 mM CaCl_2_ (pH 7.4)) with 10 pmol of complex originating from the same GST pull-down. The reaction was carried out at 50 °C and 400 rpm.

To compare the activity of different complexes, samples were taken every 15 min for 2 h. Mannanase activity was determined quantitatively by measuring the amount of reducing sugars in these samples with the DNS method. After 2, 4, 6 and 8 h, the amount of released galactose was determined with the Megazyme l-Arabinose/d-Galactose assay kit. After 6 and 8 h, the amount of released mannose was determined with the d-Mannose/d-Fructose/d-Glucose assay kit. In all cases, samples were analysed in 96-well microtiter plates.

The data obtained from all activity assays were expressed as the mean ± standard deviation (SD) of the three technical replicates. The student *t*-test or Analysis of variance (ANOVA) was used to compare the differences at a level of significance *p* < 0.05 (SPSS-PC + 11.0 software, Chicago, IL, USA).

## Supplementary Information

**Additional file 1: Figure S1**: VersaTile follows a two-step approach. **Figure S2.** VersaTile shuffling – Three-way system to construct docking enzymes. **Table S1.** Position tags used for the construction of docking enzymes. **Figure S3.** VersaTile shuffling – Five-way system to construct scaffoldins. **Table S2.** Position tags used for the construction of scaffoldins. **Figure S4.** Adaptation of the three-way system into a two-way docking enzyme assembly system.**Figure S5.** Adaptation of the five-way system into a four-, three- or two-way scaffoldin assembly system. **Table S3.** Cohesin-dockerin pairs present in the tile repository. **Table S4.** CBM tiles present in the tile repository. **Table S5.** Linker tiles present in the tile repository. **Figure S6.** Overview of constructed destination vectors. **Table S6. **GM-degrading enzymes. **Table S7.** (Docking) enzyme variants constructed in this study. **Table S8.** Scaffoldin variants constructed in this study. **Figure S7.** Influence of dockerin position on the expression and purification yield of mannanase docking enzymes. **Figure S8.** Influence of dockerin position on the expression and purification yield of mannosidase docking enzymes. **Figure S9.** Influence of dockerin position on the expression and purification of galactosidase docking enzymes. **Figure S10.** SDS-PAGE analysis of fractions obtained after GST pull-down (1). **Figure S11.** SDS-PAGE analysis of fractions obtained after GST pull-down (2). **Figure S12.** SDS-PAGE analysis of fractions obtained after GST pull-down (3). **Figure S13.** SDS-PAGE analysis of fractions obtained after GST pull-down (4). **Figure S14.** SDS-PAGE of fractions obtained after GST pull-down (5). **Table S9.** Overview of primers used to amplify dockerin tiles. **Table S10.** Overview of primers used to amplify cohesin tiles. **Table S11.** Overview of primers used to amplify CBM tiles. **Table S12.** Overview of primers used to amplify linker tiles. **Table S13.** Overview of primers used to amplify tag tiles. **Table S14.** Overview of primers used to amplify tiles encoding GM-degrading enzymes. **Table S15.** Primers used in the inverse PCR to remove the BsaI recognition site.

## Data Availability

All data generated or analysed during this study are included in this published article and its additional information files.

## References

[1] Arevalo-Gallegos A, Ahmad Z, Asgher M, Parra-Saldivar R, Iqbal HMN (2017). Lignocellulose: a sustainable material to produce value-added products with a zero waste approach—a review. Int J Biol Macromol.

[2] Van Dyk JS, Pletschke BI (2012). A review of lignocellulose bioconversion using enzymatic hydrolysis and synergistic cooperation between enzymes—factors affecting enzymes, conversion and synergy. Biotechnol Adv.

[3] Bayer EA, Morag E, Lamed R (1994). The cellulosome—a treasure-trove for biotechnology. Trends Biotechnol.

[4] Artzi L, Bayer EA, Morais S (2017). Cellulosomes: bacterial nanomachines for dismantling plant polysaccharides. Nat Rev Microbiol.

[5] Fierobe HP, Mechaly A, Tardif C, Belaich A, Lamed R, Shoham Y, Belaich JP, Bayer EA (2001). Design and production of active cellulosome chimeras—selective incorporation of dockerin-containing enzymes into defined functional complexes. J Biol Chem.

[6] Morais S, Barak Y, Caspi J, Hadar Y, Lamed R, Shoham Y, Wilson DB, Bayer EA (2010). Cellulase-xylanase synergy in designer cellulosomes for enhanced degradation of a complex cellulosic substrate. MBio.

[7] Stern J, Kahn A, Vazana Y, Shamshoum M, Morais S, Lamed R, Bayer EA (2015). Significance of relative position of cellulases in designer cellulosomes for optimized cellulolysis. PLoS ONE.

[8] Kahn A, Morais S, Galanopoulou AP, Chung D, Sarai NS, Hengge N, Hatzinikolaou DG, Himmel ME, Bomble YJ, Bayer EA (2019). Creation of a functional hyperthermostable designer cellulosome. Biotechnol Biofuels.

[9] Vanderstraeten J, Briers Y (2020). Synthetic protein scaffolds for the colocalisation of co-acting enzymes. Biotechnol Adv.

[10] Morais S, Stern J, Kahn A, Galanopoulou AP, Yoav S, Shamshoum M, Smith MA, Hatzinikolaou DG, Arnold FH, Bayer EA (2016). Enhancement of cellulosome-mediated deconstruction of cellulose by improving enzyme thermostability. Biotechnol Biofuels.

[11] Kahn A, Morais S, Chung D, Sarai NS, Hengge NN, Himmel ME, Bayer EA, Bomble YJ (2020). Glycosylation of hyperthermostable designer cellulosome components yields enhanced stability and cellulose hydrolysis. Febs J.

[12] Vazana Y, Barak Y, Unger T, Peleg Y, Shamshoum M, Ben-Yehezkel T, Mazor Y, Shapiro E, Lamed R, Bayer EA (2013). A synthetic biology approach for evaluating the functional contribution of designer cellulosome components to deconstruction of cellulosic substrates. Biotechnol Biofuels.

[13] Kahn A, Bayer EA, Moraïs S. Advanced cloning tools for construction of designer cellulosomes. In: Cellulases*.* Springer New York, New York; 2018; pp 135–151. 10.1007/978-1-4939-7877-9_11 29856052

[14] Gerstmans H, Grimon D, Gutiérrez D, Lood C, Rodríguez A, van Noort V, Lammertyn J, Lavigne R, Briers Y (2020). A VersaTile driven platform for rapid hit-to-lead development of engineered lysins. Sci Adv.

[15] Yamabhai M, Sak-Ubol S, Srila W, Haltrich D (2016). Mannan biotechnology: from biofuels to health. Crit Rev Biotechnol.

[16] Moreira LRS, Filho EXF (2008). An overview of mannan structure and mannan-degrading enzyme systems. Appl Microbiol Biot.

[17] Srivastava PK, Kapoor M (2017). Production, properties, and applications of endo-beta-mannanases. Biotechnol Adv.

[18] Caspi J, Irwin D, Lamed R, Li YC, Fierobe HP, Wilson DB, Bayer EA (2008). Conversion of *Thermobifida fusca* free exoglucanases into cellulosomal components: comparative impact on cellulose-degrading activity. J Biotechnol.

[19] Morais S, Barak Y, Hadar Y, Wilson DB, Shoham Y, Lamed R, Bayer EA (2011). Assembly of xylanases into designer cellulosomes promotes efficient hydrolysis of the xylan component of a natural recalcitrant cellulosic substrate. MBio.

[20] Hilge M, Gloor SM, Rypniewski W, Sauer O, Heightman TD, Zimmermann W, Winterhalter K, Piontek K (1998). High-resolution native and complex structures of thermostable beta-mannanase from *Thermomonospora fusca*—substrate specificity in glycosyl hydrolase family 5. Structure.

[21] Toth A, Barna T, Szabo E, Elek R, Hubert A, Nagy I, Kriszt B, Tancsics A, Kukolya J (2016). Cloning, expression and biochemical characterization of endomannanases from *Thermobifida* species isolated from different niches. PLoS ONE.

[22] Beki E, Nagy S, Vanderleyden J, Jager S, Kiss L, Fulop L, Hornok L, Kukolya J (2003). Cloning and heterologous expression of a beta-D-mannosidase (EC 3.2.1.25)-encoding gene from *Thermobifida fusca* TM51. Appl Environ Microbiol.

[23] Tunnicliffe RB, Bolam DN, Pell G, Gilbert HJ, Williamson MP (2005). Structure of a mannan-specific family 35 carbohydrate-binding module: evidence for significant conformational changes upon ligand binding. J Mol Biol.

[24] Bolam DN, Xie HF, Pell G, Hogg D, Galbraith G, Henrissat B, Gilbert HJ (2004). X4 modules represent a new family of carbohydrate-binding modules that display novel properties. J Biol Chem.

[25] Finn RD, Clements J, Eddy SR (2011). HMMER web server: interactive sequence similarity searching. Nucleic Acids Res.

[26] Kelley LA, Mezulis S, Yates CM, Wass MN, Sternberg MJE (2015). The Phyre2 web portal for protein modeling, prediction and analysis. Nat Protoc.

[27] Fierobe HP, Bayer EA, Tardif C, Czjzek M, Mechaly A, Belaich A, Lamed R, Shoham Y, Belaich JP (2002). Degradation of cellulose substrates by cellulosome chimeras—substrate targeting versus proximity of enzyme components. J Biol Chem.

[28] Mingardon F, Chanal A, Tardif C, Bayer EA, Fierobe HP (2007). Exploration of new geometries in cellulosome-like chimeras. Appl Environ Microbiol.

[29] Raghothama S, Eberhardt RY, Simpson P, Wigelsworth D, White P, Hazlewood GP, Nagy T, Gilbert HJ, Williamson MP (2001). Characterization of a cellulosome dockerin domain from the anaerobic fungus *Piromyces equi*. Nat Struct Biol.

[30] Grépinet O, Chebrou MC, Béguin P (1988). Nucleotide sequence and deletion analysis of the xylanase gene (*xynZ*) of *Clostridium thermocellum*. J Bacteriol.

[31] Caspi J, Barak Y, Haimovitz R, Irwin D, Lamed R, Wilson DB, Bayer EA (2009). Effect of linker length and dockerin position on conversion of a *Thermobifida fusca* endoglucanase to the cellulosomal mode. Appl Environ Microbiol.

[32] Bayer EA, Shoham Y, Lamed R, Rosenberg E (2013). Lignocellulose-decomposing bacteria and their enzyme systems. The prokaryotes.

[33] Xu Q, Ding SY, Brunecky R, Bomble YJ, Himmel ME, Baker JO (2013). Improving activity of minicellulosomes by integration of intra- and intermolecular synergies. Biotechnol Biofuels.

[34] Davidi L, Morais S, Artzi L, Knop D, Hadar Y, Arfi Y, Bayer EA (2016). Toward combined delignification and saccharification of wheat straw by a laccase-containing designer cellulosome. Proc Natl Acad Sci U S A.

[35] Stern J, Artzi L, Morais S, Fontes CMGA, Bayer EA (2017). Carbohydrate depolymerization by intricate cellulosomal systems. Methods Mol Biol.

[36] Jumper J, Evans R, Pritzel A, Green T, Figurnov M, Ronneberger O, Tunyasuvunakool K, Bates R, Žídek A, Potapenko A (2021). Highly accurate protein structure prediction with AlphaFold. Nature.

[37] Malgas S, van Dyk JS, Pletschke BI (2015). A review of the enzymatic hydrolysis of mannans and synergistic interactions between beta-mannanase, beta-mannosidase and alpha-galactosidase. World J Microbiol Biotechnol.

[38] Margolles Clark E, Tenkanen M, Luonteri E, Penttila M (1996). Three alpha-galactosidase genes of *Trichoderma reesei* cloned by expression in yeast. Eur J Biochem.

[39] Cervero JM, Skovgaard PA, Felby C, Sorensen HR, Jorgensen H (2010). Enzymatic hydrolysis and fermentation of palm kernel press cake for production of bioethanol. Enzyme Microb Technol.

[40] Bayer EA, Smith SP, Noach I, Alber O, Adams JJ, Lamed R, Shimon LJW, Frolow F, Sakka K, Karita S, Kimura T, Sakka M, Matsui H, Miyake H, Tanaka A (2009). Can we crystallize a cellulosome?. Biotechnology of lignocellulose degradation and biomass utilization.

[41] Smith SP, Bayer EA (2013). Insights into cellulosome assembly and dynamics: from dissection to reconstruction of the supramolecular enzyme complex. Curr Opin Struct Biol.

[42] Smith SP, Bayer EA, Czjzek M (2017). Continually emerging mechanistic complexity of the multi-enzyme cellulosome complex. Curr Opin Struct Biol.

[43] Hammel M, Fierober HP, Czjzek M, Kurkal V, Smith JC, Bayer EA, Finet S, Receveur-Brechot V (2005). Structural basis of cellulosome efficiency explored by small angle X-ray scattering. J Biol Chem.

[44] Dueber JE, Wu GC, Malmirchegini GR, Moon TS, Petzold CJ, Ullal AV, Prather KLJ, Keasling JD (2009). Synthetic protein scaffolds provide modular control over metabolic flux. Nat Biotechnol.

[45] Moon TS, Dueber JE, Shiue E, Prather KLJ (2010). Use of modular, synthetic scaffolds for improved production of glucaric acid in engineered *E. coli*. Metab Eng.

[46] Baek JM, Mazumdar S, Lee SW, Jung MY, Lim JH, Seo SW, Jung GY, Oh MK (2013). Butyrate production in engineered *Escherichia coli* with synthetic scaffolds. Biotechnol Bioeng.

[47] Fierobe HP, Mingardon F, Mechaly A, Belaich A, Rincon MT, Pages S, Lamed R, Tardif C, Belaich JP, Bayer EA (2005). Action of designer cellulosomes on homogeneous *versus* complex substrates—controlled incorporation of three distinct enzymes into a defined trifunctional scaffoldin. J Biol Chem.

[48] Borne R, Bayer EA, Pages S, Perret S, Fierobe HP (2013). Unraveling enzyme discrimination during cellulosome assembly independent of cohesin-dockerin affinity. Febs J.

[49] Stern J, Morais S, Lamed R, Bayer EA (2016). Adaptor scaffoldins: an original strategy for extended designer cellulosomes, inspired from nature. MBio.

[50] Tsai SL, DaSilva NA, Chen W (2013). Functional display of complex cellulosomes on the yeast surface via adaptive assembly. ACS Synth Biol.

[51] Tian S, Du JL, Bai ZS, He J, Yang XS (2019). Design and construction of synthetic cellulosome with three adaptor scaffoldins for cellulosic ethanol production from steam-exploded corn stover. Cellulose.

