# Supplementary material for: Combinatorial assembly and optimisation of designer cellulosomes: a galactomannan case study
Source: Biotechnol Biofuels Bioprod. 2022 May 30;15:60. doi: 10.1186/s13068-022-02158-2 (PMC9153192; doi:10.1186/s13068-022-02158-2)
Supplement: Supplementary file 1 — Additional file 1: Figure S1: VersaTile follows a two-step approach. Figure S2. VersaTile shuffling – Three-way system to construct docking enzymes. Table S1. Position tags used for the construction of docking enzymes. Figure S3. VersaTile shuffling – Five-way system to construct scaffoldins. Table S2. Position tags used for the construction of scaffoldins. Figure S4. Adaptation of the three-way system into a two-way docking enzyme assembly system.Figure S5. Adaptation of the five-way system into a four-, three- or two-way scaffoldin assembly system. Table S3. Cohesin-dockerin pairs present in the tile repository. Table S4. CBM tiles present in the tile repository. Table S5. Linker tiles present in the tile repository. Figure S6. Overview of constructed destination vectors. Table S6. GM-degrading enzymes. Table S7. (Docking) enzyme variants constructed in this study. Table S8. Scaffoldin variants constructed in this study. Figure S7. Influence of dockerin position on the expression and purification yield of mannanase docking enzymes. Figure S8. Influence of dockerin position on the expression and purification yield of mannosidase docking enzymes. Figure S9. Influence of dockerin position on the expression and purification of galactosidase docking enzymes. Figure S10. SDS-PAGE analysis of fractions obtained after GST pull-down (1). Figure S11. SDS-PAGE analysis of fractions obtained after GST pull-down (2). Figure S12. SDS-PAGE analysis of fractions obtained after GST pull-down (3). Figure S13. SDS-PAGE analysis of fractions obtained after GST pull-down (4). Figure S14. SDS-PAGE of fractions obtained after GST pull-down (5). Table S9. Overview of primers used to amplify dockerin tiles. Table S10. Overview of primers used to amplify cohesin tiles. Table S11. Overview of primers used to amplify CBM tiles. Table S12. Overview of primers used to amplify linker tiles. Table S13. Overview of primers used to amplify tag tiles. Table S14. Overview of primers used to amplify [file 13068_2022_2158_MOESM1_ESM.docx]

**Supplementary material**

Figures and tables are ordered according to their appearance in the main manuscript.


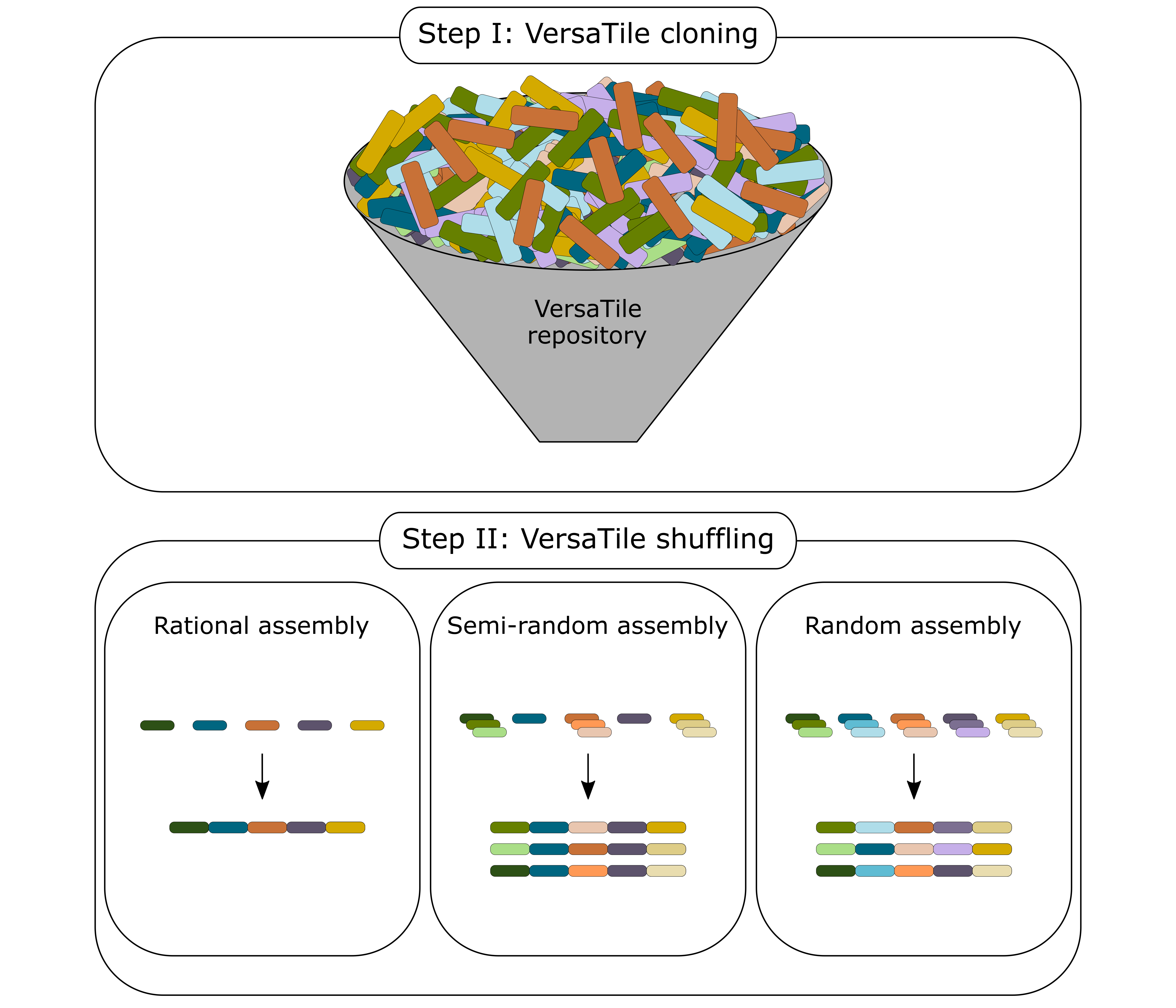


**Figure S1: VersaTile follows a two-step approach.** First, a repository of tiles is constructed in a protocol termed VersaTile cloning (top panel). Second, any selection of tiles can be assembled in a protocol termed VersaTile shuffling (bottom panel). When using rational assembly, every fragment has its fixed position. In the semi-random assembly, there are several fragments in the mixture for one or more positions, whereas other positions are still fixed. In random assembly, different fragments for each position are mixed, resulting in the highest variability.


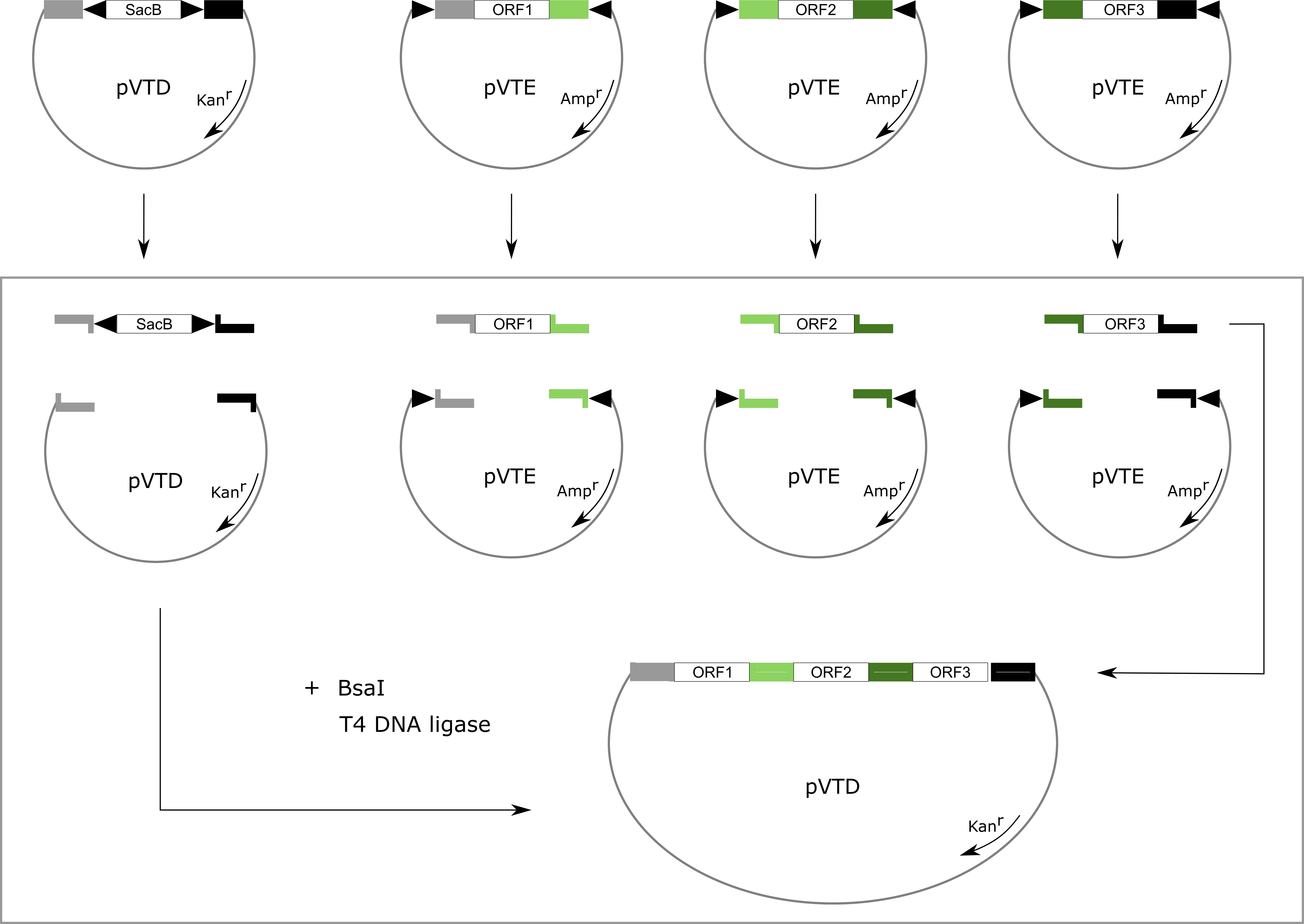


**Figure S2: VersaTile shuffling – Three-way system to construct docking enzymes.** pVTD contains the SacB negative selection marker, flanked by the BsaI recognition site and restriction site (or position tag). Each of the three selected entry vectors, pVTE, contains a specific ORF (dockerins, linkers, enzymes, tags), flanked by position tags (DE_s_: grey, DE_2_: light green, DE_3_: dark green, DE_e_: black) and the BsaI recognition site (black arrowhead). The black arrowhead indicating the BsaI restriction site points towards the restriction site. Note that the orientation differs between pVTD and pVTE. The restriction and ligation occur in one step by mixing the selected pVTEs and pVTD with BsaI and T4 DNA ligase. All vectors are digested by BsaI, resulting in complementary sticky ends, which are ligated by T4 DNA ligase. The resulting vector encodes a docking enzyme composed of the three selected tiles.

**Table S1: Position tags used for the construction of docking enzymes.** A three-way system for the construction of docking enzymes was developed. The first column shows the position of the tiles in the final construct. The second column gives the name of the tile, where ‘X’ is the name of the enzyme, dockerin, linker or his-tag flanked by the position tag codes. The position tag codes indicate the assembly system (docking enzyme = DE) and the specific position of the tile in the final construct (start = s, position 2 = 2, position 3 = 3, end = e). The third and fourth columns show the nucleotide sequence at the 5’ and 3’ termini of the tiles. The translated amino acid sequence is displayed parenthetically.

| **Position** | **Tile name** | **5’ terminus of tile** | **3’ terminus of tile** |
| --- | --- | --- | --- |
| 1 | DE_s__X_DE_2_ | ACCATG (-M) | GGTTCA (GS) |
| 2 | DE_2__X_DE_3_ | GGTTCA (GS) | TCTGGT (SG) |
| 3 | DE_3__X_DE_e_ | TCTGGT (SG) | AAGTAT (K-) |


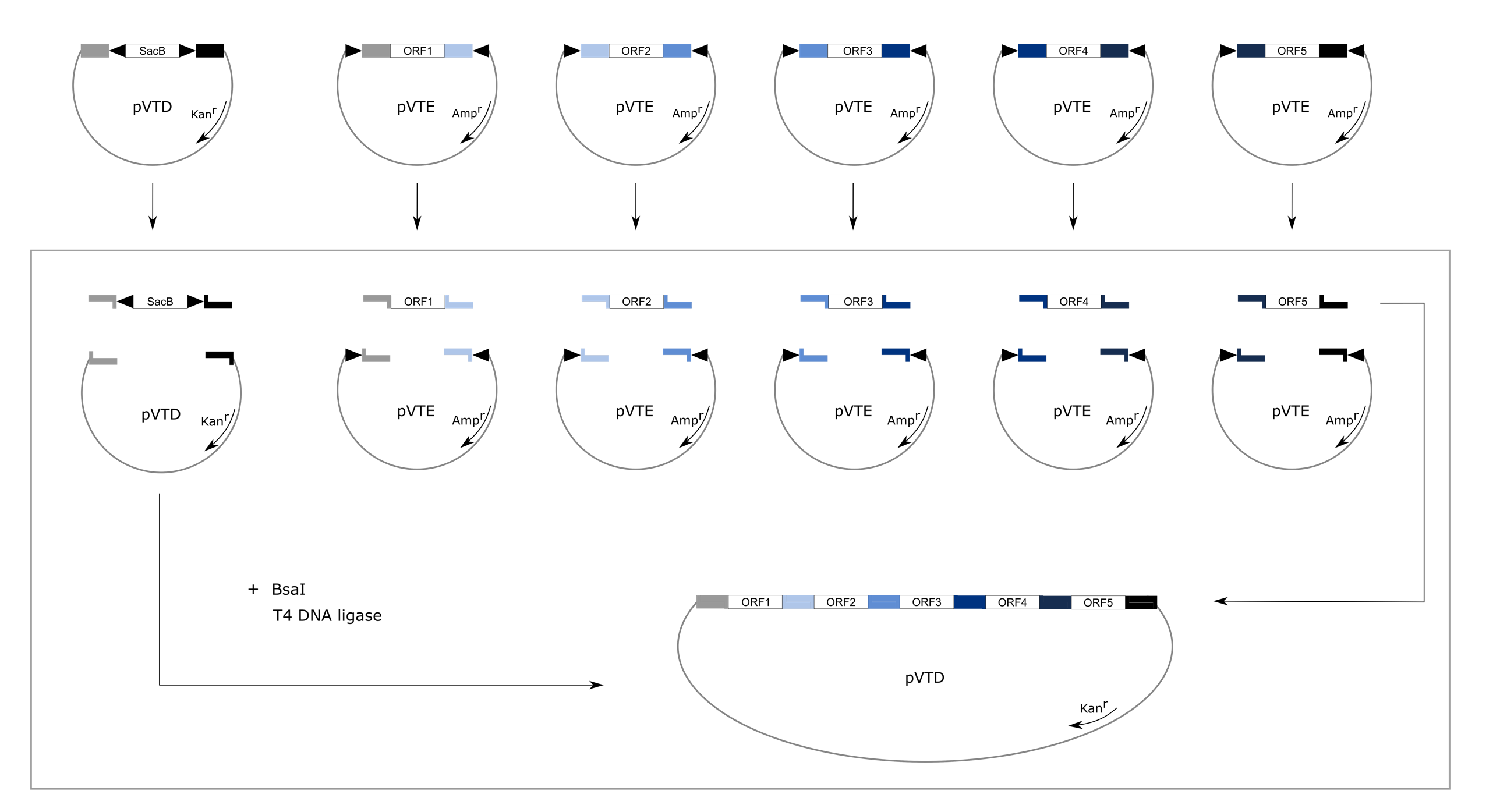


**Figure S3: VersaTile shuffling – Five-way system to construct scaffoldins.** pVTD contains the SacB negative selection marker, flanked by the BsaI recognition site and restriction site (or position tag). Each of the five selected entry vectors, pVTE, contains a specific ORF (cohesins or CBMs), flanked by position tags (Sc_s_: grey, Sc_2_, Sc_3_, Sc_4_ and Sc_5_: light to dark blue, Sc_e_: black) and the BsaI recognition site (black arrowhead). The black arrowhead indicating the BsaI restriction site points towards the restriction site. Note that the orientation differs between pVTD and pVTE. The restriction and ligation occur in one step by mixing the selected pVTEs and pVTD with BsaI and T4 DNA ligase. All vectors are digested by BsaI, resulting in complementary sticky ends, which are ligated by T4 DNA ligase. The resulting vector encodes a scaffoldin composed of the five selected tiles.

**Table S2: Position tags used for the construction of scaffoldins.** A five-way system for the construction of scaffoldins was developed. The first column shows the position of the tiles in the final construct. The second column gives the name of the tile where ‘X’ is the name of the cohesin, CBM or his-tag flanked by the position tag codes. The position tag codes indicate the assembly system (Scaffoldin = Sc) and the specific position of the tile in the final construct (start = s, position 2 = 2, position 3 = 3, position 4 = 4, position 5 = 5, end = e). The third and fourth columns show the nucleotide sequence at the 5’ and 3’ termini of the tiles. The translated amino acid sequence is displayed parenthetically.

| **Position** | **Tile name** | **5’ terminus of tile** | **3’ terminus of tile** |
| --- | --- | --- | --- |
| 1 | Sc_s__X_Sc_2_ | ACCATG (-M) | AGCACA (ST) |
| 2 | Sc_2__X_Sc_3_ | AGCACA (ST) | CCAACG (PT) |
| 3 | Sc_3__X_Sc_4_ | CCAACG (PT) | ACGAGC (TS) |
| 4 | Sc_4__X_Sc_5_ | ACGAGC (TS) | CCGTCT (PS) |
| 5 | Sc_5__X_Sc_e_ | CCGTCT (PS) | AAGTAA (K-) |


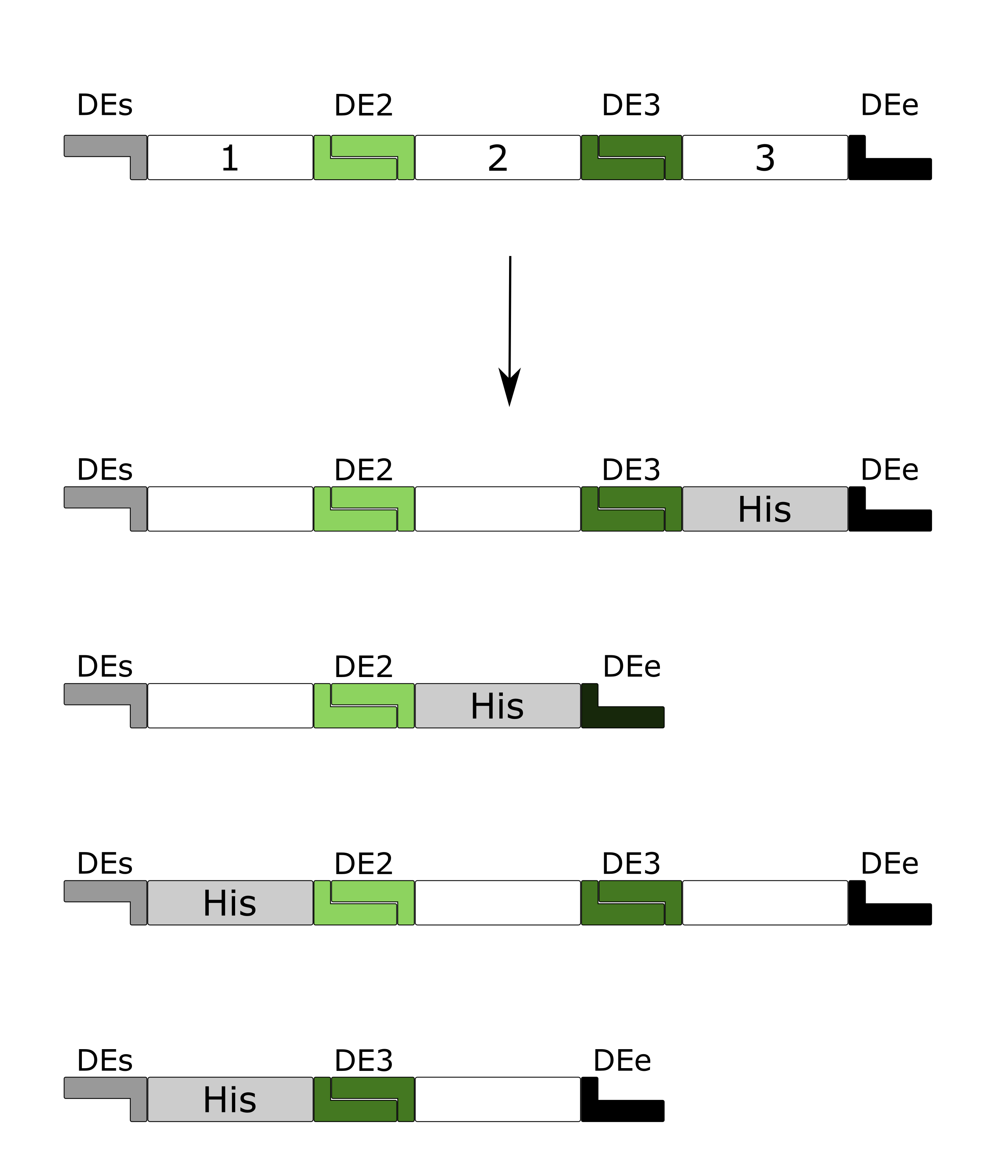


**Figure S4: Adaptation of the three-way system into a two-way docking enzyme assembly system.** His-tag coding sequences flanked by several combinations of position tags can be used to convert the three-way docking enzyme construction system into a two-way system. Depending on the position tags, His-tags can be placed C- or N- terminally.


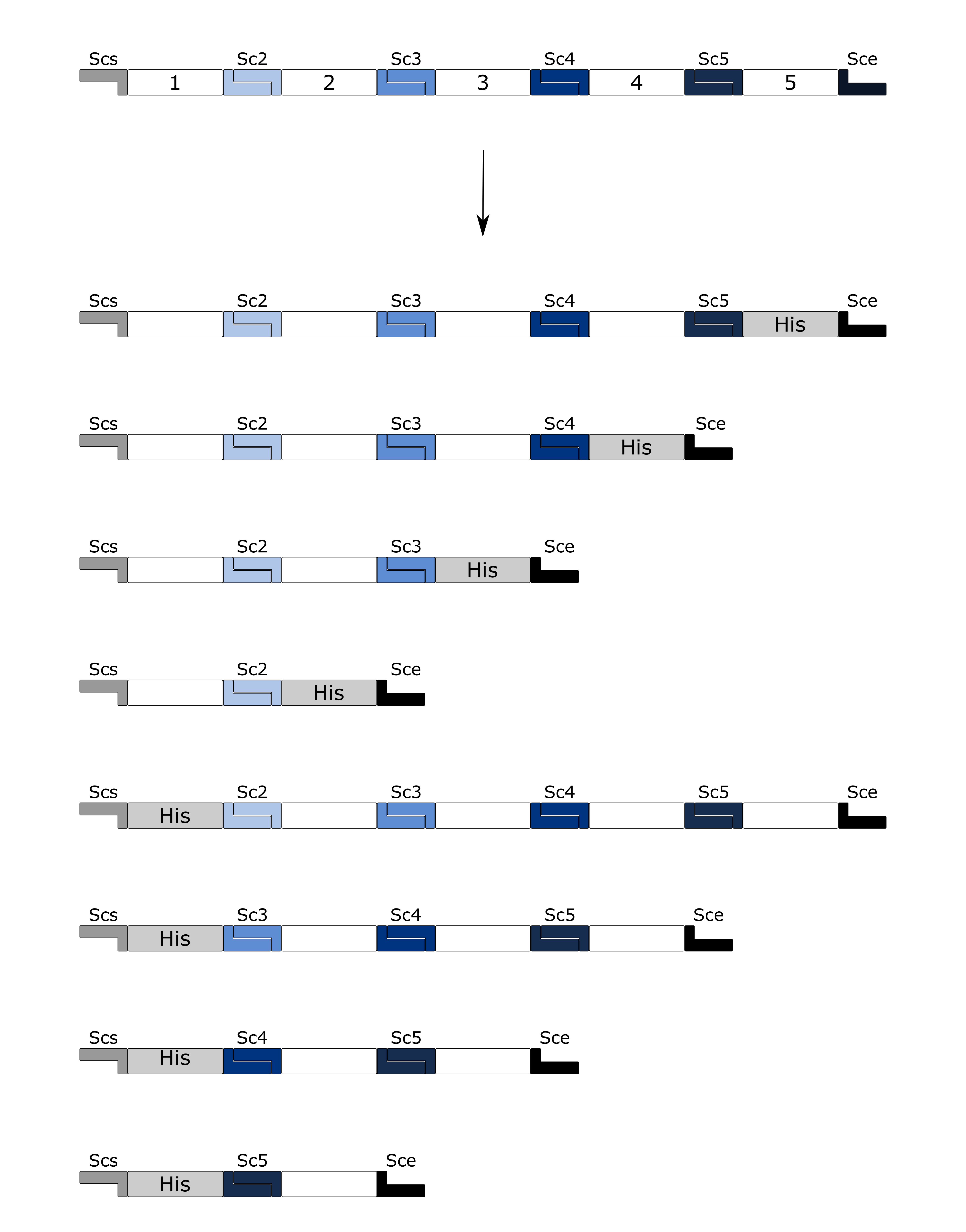


**Figure S5: Adaptation of the five-way system into a four-, three- or two-way scaffoldin assembly system.** His-tags flanked by several combinations of position tags can be used to convert the five-way scaffoldin construction system into a four-, three- or two-way system. His-tags can be placed C- or N- terminally.

**Table S3: Cohesin-dockerin pairs present in the tile repository.** Five variants of each cohesin tile and three variants of each dockerin tile were constructed to allow assembly at each position in the scaffoldin and docking enzyme assembly systems. The first column shows the organism of origin of the selected cohesin-dockerin pairs. Columns two and three describe the protein of origin of the dockerin and its GenBank accession number. The fourth and fifth columns show the code and icon that is used for each dockerin throughout this study. The sixth column illustrates at which position in the docking enzyme assembly system the tiles can be arranged. Columns seven and eight describe the protein of origin of the cohesin and its GenBank code. The ninth and tenth columns show the code and icon that is used for each cohesin throughout this study. The final column illustrates at which position in the scaffoldin assembly system the tiles can be arranged.

| **Organism of origin** | **Dockerin** | | | | | | | **Cohesin** | | | | | | | | | |
| --- | --- | --- | --- | --- | --- | --- | --- | --- | --- | --- | --- | --- | --- | --- | --- | --- | --- |
|  | **Description** | **GenBank** | **Code** | **Icon** | **Present at position** | | | **Description** | **GenBank** | **Code** | **Icon** | **Present at position** | | | | |  |
|  |  |  |  |  | **1** | **2** | **3** |  |  |  |  | **1** | **2** | **3** | **4** | **5** |  |
| *Clostridium thermocellum* | Cel48S dockerin | [ABN53296.1](https://www.ncbi.nlm.nih.gov/protein/ABN53296.1) | Doc-*Ct*I | 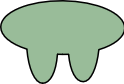 | ✓ | ✓ | ✓ | CipA  cohesin nº2 | [CCV01465.1](https://www.ncbi.nlm.nih.gov/protein/CCV01465.1) | Coh-*Ct*I | 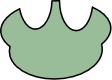 | ✓ | ✓ | ✓ | ✓ | ✓ |  |
|  | XynZ dockerin | [ABN53181.1](https://www.ncbi.nlm.nih.gov/protein/ABN53181.1) | Doc-*Ct*NI | 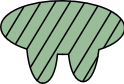 | ✓ | ✓ |  |  |  |  |  | ✓ | ✓ | ✓ | ✓ | ✓ |  |
|  | CipA dockerin | [CCV01465.1](https://www.ncbi.nlm.nih.gov/protein/CCV01465.1) | Doc-*Ct*II | 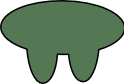 | ✓ | ✓ | ✓ | OlpB  cohesin nº4 | [WP_059169945.1](https://www.ncbi.nlm.nih.gov/protein/WP_059169945.1) | Coh-*Ct*II | 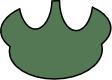 | ✓ | ✓ | ✓ | ✓ | ✓ |  |
| *Ruminococcus flavefaciens* | EndB dockerin | [CAC83072.1](https://www.ncbi.nlm.nih.gov/protein/CAC83072.1) | Doc-*Rf* | 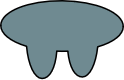 | ✓ | ✓ | ✓ | ScaA  cohesin nº2 | [CAC34384.3](https://www.ncbi.nlm.nih.gov/protein/CAC34384.3) | Coh-*Rf* | 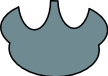 | ✓ | ✓ | ✓ | ✓ | ✓ |  |
| *Clostridium cellulolyticum* | Cel5A dockerin | [AAA23221.1](https://www.ncbi.nlm.nih.gov/protein/AAA23221.1) | Doc-*Cc* | 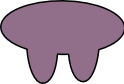 | ✓ | ✓ | ✓ | CipC  cohesin nº1 | [AAC28899.2](https://www.ncbi.nlm.nih.gov/protein/AAC28899.2) | Coh-*Cc* | 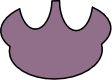 | ✓ | ✓ | ✓ | ✓ | ✓ |  |
| *Acetivibrio cellulolyticus* | ScaB dockerin | [AAP48995.1](https://www.ncbi.nlm.nih.gov/protein/AAP48995.1) | Doc-*Ac* | 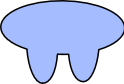 | ✓ | ✓ | ✓ | ScaC  cohesin nº3 | [AAP48996.1](https://www.ncbi.nlm.nih.gov/protein/AAP48996.1) | Coh-*Ac* | 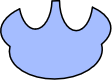 | ✓ | ✓ | ✓ | ✓ | ✓ |  |
| *Bacteroides cellulosolvens* | ScaA dockerin | [AAG01230.2](https://www.ncbi.nlm.nih.gov/protein/AAG01230.2) | Doc-*Bc* | 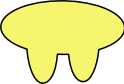 | ✓ | ✓ | ✓ | ScaB  cohesin nº3 | [AAT79550.1](https://www.ncbi.nlm.nih.gov/protein/AAT79550.1) | Coh-*Bc* | 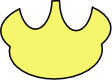 | ✓ | ✓ | ✓ | ✓ | ✓ |  |
| *Archaeoglobus fulgidus* | Non- cellulosomal dockerin | [WP_010879862.1](https://www.ncbi.nlm.nih.gov/protein/WP_010879862.1) | Doc-*Af* | 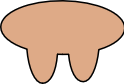 | ✓ | ✓ | ✓ | Non- cellulosomal cohesin | [WP_010879862.1](https://www.ncbi.nlm.nih.gov/protein/WP_010879862.1) | Coh-*Af* | 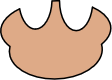 | ✓ | ✓ | ✓ | ✓ | ✓ |  |

**Table S4: CBM tiles present in the tile repository.** The first column shows the organism of origin. Columns two and three describe the protein of origin and its GenBank code. The fourth column shows the CBM family and the fifth and sixth columns show the code and icon that is used for each CBM throughout this study. The final column illustrates at which position in the scaffoldin assembly system the tiles can be arranged.

| **Organism of origin** | **Description** | **GenBank** | **CBM family** | **Code** | **Icon** | **Present at position** | | | | |
| --- | --- | --- | --- | --- | --- | --- | --- | --- | --- | --- |
|  |  |  |  |  |  | **1** | **2** | **3** | **4** | **5** |
| *Clostridium thermocellum* | CipA | [CCV01465.1](https://www.ncbi.nlm.nih.gov/protein/CCV01465.1) | CBM3a | *Ct*-CBM3 | 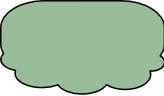 | ✓ | ✓ | ✓ | ✓ | ✓ |
| *Cellvibrio japonicus* | Man5C | [ACE82655.1](https://www.ncbi.nlm.nih.gov/protein/ACE82655.1) | CBM35 | *Cj-*CBM35 | 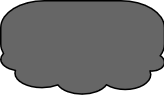 | ✓ | ✓ | ✓ | ✓ | ✓ |

**Table S****5: Linker tiles present in the tile repository.** The first, second, third and fourth columns show the linker code, Protein Data Bank (PDB) code (for non-synthetic linkers), sequence and length in amino acids. The fifth column gives the number of prolines present in the sequence. The sixth column indicates if the linker exhibits a rigid or flexible structure. The seventh column shows the predicted pI of the linker. The icon that is used for each linker throughout this study is displayed in the final column. All linker tiles are designed to be fitted at the second position in the three-way docking enzyme assembly system.

| **Code** | **Sequence** | **Length** | **Prolines** | **Structure** | **pI** | **Icon** |
| --- | --- | --- | --- | --- | --- | --- |
| Li-A | LSRFFHAEL | 9 | 0 | Rigid (helical) | 6.75 | 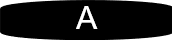 |
| Li-B | VFNQRKEHKGYMLA | 14 | 0 | Rigid (helical) | 9.7 | 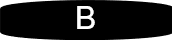 |
| Li-C | IPQGRSHPVQPYPGAF | 16 | 4 | Rigid (coil) | 8.75 | 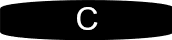 |
| Li-D | PAVPPP | 6 | 4 | Rigid (coil) | 5.96 | 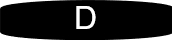 |
| Li-E | GGGGSGGGGSGGGGS | 15 | 0 | Flexible (coil) | 5.52 | 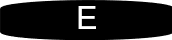 |
| Li-F | GGGGGGGG | 8 | 0 | Flexible (coil) | 5.52 | 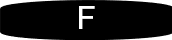 |
| Li-G | EAAAKEAAAKEAAAK | 15 | 0 | Rigid (helical) | 6.33 | 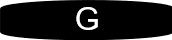 |
| Li-H | APAPAPAPAP | 10 | 5 | Rigid (coil) | 5.57 | 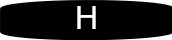 |
| Li-I | EAAAKEAAKEAAK | 13 | 0 | Rigid (helical) | 6.33 | 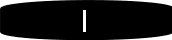 |

**
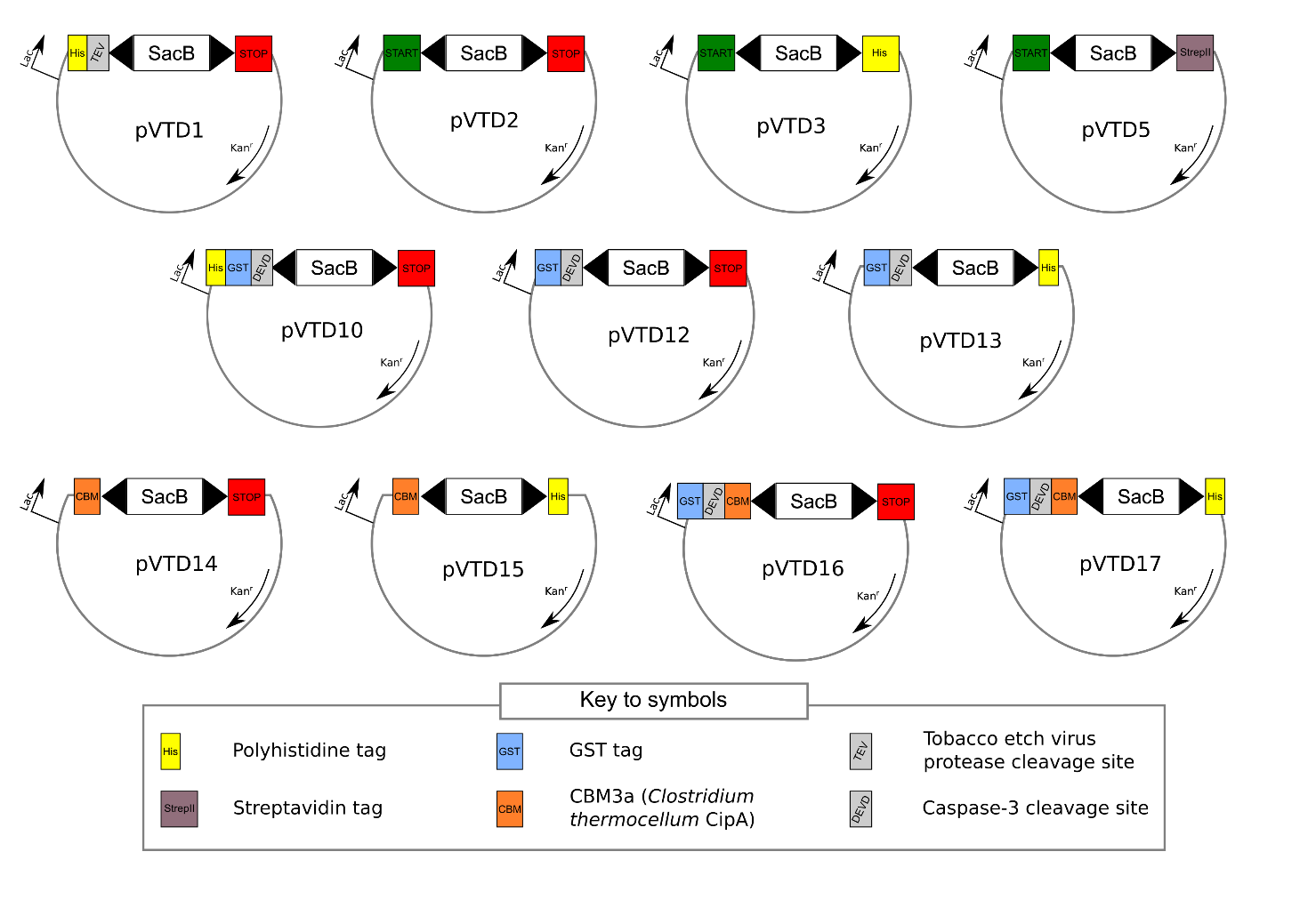
**

**Figure S6: Overview of constructed destination vectors.** Each destination vector allows the addition of specific (cleavable) N-or C-terminal tags to the protein consisting of selected tiles. pVTD1: N-terminal His tag, pVTD2: no added tags, pVTD3: C-terminal His tag, pVTD5: C-terminal StrepII-tag, pVTD10: N-terminal His- and GST-tag, pVTD12: N-terminal GST-tag, pVTD13: N-terminal GST-tag and C-terminal His-tag, pVTD14: N-terminal CBM, pVTD15: N-terminal CBM and C-terminal His-tag, pVTD16: N-terminal GST-tag and CBM, pVTD17: N-terminal GST-tag and CBM and C-terminal His-tag.

**Table** **S6: GM-degrading enzymes.** The presented enzymes were converted to the cellulosomal mode. The first, second and third columns give the name, the organism of origin and the GenBank code of each enzyme. The fourth column indicates the GH (and in some cases CBM) family. The fifth and sixth columns mention the pH and temperature optimum (or range) of the enzyme. Codes for the specific tiles that were constructed are listed in the seventh column. The eighth column shows the icon that is used for each enzyme tile and the ninth column refers to the study in which the enzyme was characterised.

| **Enzyme** | **Organism of origin** | **GenBank** | **GH family** | **pH** | **Temperature (°C)** | **Code** | **Icon** | **Reference** |
| --- | --- | --- | --- | --- | --- | --- | --- | --- |
| β-1,4-mannanase | *Thermobifida fusca* | [AAZ54938.1](https://www.ncbi.nlm.nih.gov/protein/AAZ54938.1?report=genpept) | GH5,CBM2 | 8  (range 4.5-10) | 75  (range 50-90) | *Tf*-Manna-S | 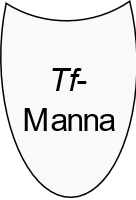 | [1, 2] |
|  |  |  |  |  |  | *Tf*-Manna | 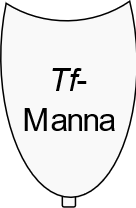 |  |
|  |  |  |  |  |  | *Tf*-Manna-Li | 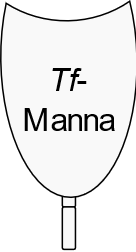 |  |
|  |  |  |  |  |  | *Tf*-Manna-CBM | 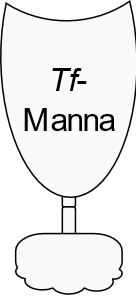 |  |
| β-1,4-mannosidase | *Thermobifida fusca* | [AAZ54953.1](https://www.ncbi.nlm.nih.gov/protein/AAZ54953.1?report=genpept) | GH2 | 7  (range 5-10) | 53  (range 25-65) | *Tf*-Manno | 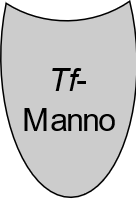 | [3] |
| α-1,6-galactosidase | *Bifidobacterium adolescentis* | [AAD30994.2](https://www.ncbi.nlm.nih.gov/protein/AAD30994.2) | GH36 | 6 | 55  (range 30-60) | *Ba*-Aga | 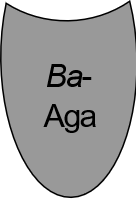 | [4, 5] |
|  | *Clostridium cellulolyticum* | [ACL75593.1](https://www.ncbi.nlm.nih.gov/protein/ACL75593.1) | GH27, CBM6 | 6.8-7.2 | 35-45 | *Cc*-Aga | 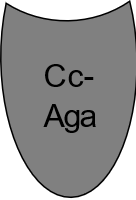 |  |
|  |  |  |  |  |  | *Cc*-Aga-Li | 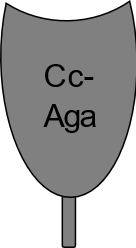 |  |
|  |  |  |  |  |  | *Cc*-Aga-CBM | 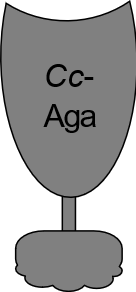 |  |
|  | *Cellvibrio japonicus* | [ACE85287.1](https://www.ncbi.nlm.nih.gov/protein/ACE85287.1) | GH27 | 8.2 | 50 | *Cj*-Aga | 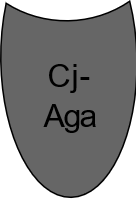 | [6] |

**Table S7: (Docking) enzyme variants constructed in this study.** The first column shows the number of the construct. The second, third and fourth colums show the tiles that were used in the assembly reaction. The docking enzyme assembly system allows the assembly of three tiles (three-way system). However, His-tag tiles overlapping multiple positions allow the assembly of two tiles. The fifth column indicates the destination vector in which the tiles were assembled (see **Figure S6:**). Note that pVTD2 was used when the His-tag sequence was added as a tile. When all positions were occupied by other tiles, assembling in pVTD1 or pVTD3 allowed the fusion of an N- or C-terminal His-tag, encoded on the vector backbone. The sixth and seventh columns show the icon (see **Table S3**, **Table S5** and **Table S6**) and molecular weight (MW) of the resulting protein. The final column indicates the expression efficiency of each docking enzyme. The expression level was assessed according to a semi-quantitative scoring system where - indicates the lack of expression, and +, ++, +++ represent low to high expression and purification yields. ND = Not determined. Thick borders are used to indicate the docking enzyme variants that were selected to be incorporated in the final designer cellulosome complex.

| **Nr.** | **Tile 1** | **Tile 2** | **Tile 3** | **Vector** | **Icon** | **MW (Da)** | **Expression & purification** |  |  |  |  |  |
| --- | --- | --- | --- | --- | --- | --- | --- | --- | --- | --- | --- | --- |
|  | **Monocatalytic: Mannanase** | | | | | | |  |  |  |  |  |
| 1 | *Tf*-Manna-S | His | | pVTD2 | 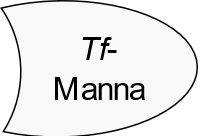 | 31993 | ++ |  |  |  |  |  |
| 2 | *Tf*-Manna | His | | pVTD2 | 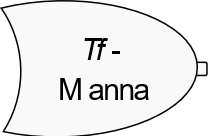 | 34615 | ++ |  |  |  |  |  |
| 3 | *Tf*-Manna-Li | His | | pVTD2 | 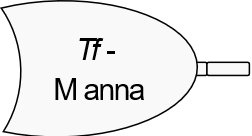 | 37242 | +++ |  |  |  |  |  |
| 4 | *Tf*-Manna-Li | Doc-*Ct*I | His | pVTD2 | 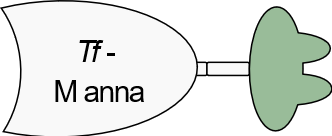 | 45480 | ++ |  |  |  |  |  |
| 5 | His | Doc-*Ct*I | *Tf*-Manna-Li | pVTD2 | 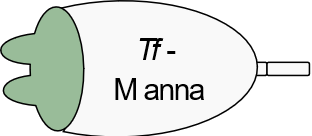 | 45480 | - |  |  |  |  |  |
| 6 | *Tf*-Manna-Li | Doc-*Ct*II | His | pVTD2 | 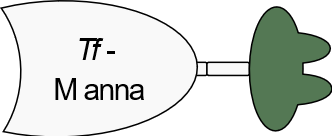 | 53772 | ++ |  |  |  |  |  |
| 7 | *Tf*-Manna-Li | Doc-*Cc* | His | pVTD2 | 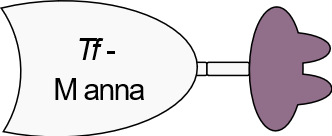 | 44773 | - |  |  |  |  |  |
| 8 | *Tf*-Manna-S | Doc-*Ct*II | His | pVTD2 | 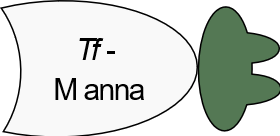 | 48524 | ++ |  |  |  |  |  |
| 9 | *Tf*-Manna-S | Li-E | Doc-*Ct*II | pVTD3 | 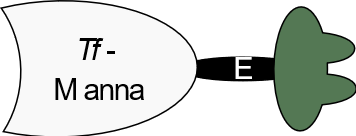 | 49488 | ++ |  |  |  |  |  |
| 10 | *Tf*-Manna-S | Li-F | Doc-*Ct*II | pVTD3 | 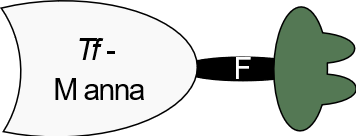 | 48998 | - |  |  |  |  |  |
| 11 | *Tf*-Manna-S | Li-G | Doc-*Ct*II | pVTD3 | 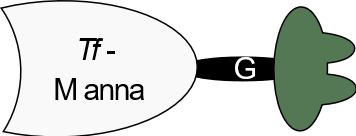 | 49953 | ++ |  |  |  |  |  |
| 12 | *Tf*-Manna-S | Li-H | Doc-*Ct*II | pVTD3 | 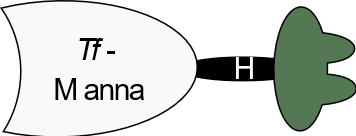 | 49383 | + |  |  |  |  |  |
| 13 | *Tf*-Manna-Li | Doc-*Ac* | His | pVTD2 | 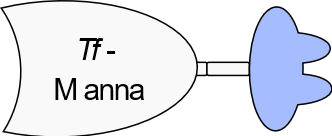 | 46065 | + |  |  |  |  |  |
| 14 | *Tf*-Manna-Li | Doc-*Af* | His | pVTD2 | 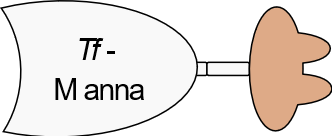 | 45556 | ND |  |  |  |  |  |
| 15 | *Tf*-Manna-Li | Li-E | Doc-*Ct*II | pVTD3 | 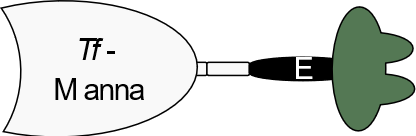 | 54736 | + |  |  |  |  |  |
| 16 | *Tf*-Manna-Li | Li-F | Doc-*Ct*II | pVTD3 | 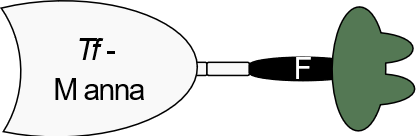 | 54247 | + |  |  |  |  |  |
| 17 | *Tf*-Manna-Li | Li-G | Doc-*Ct*II | pVTD3 | 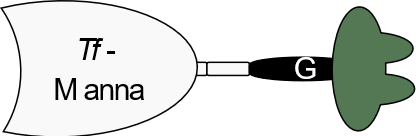 | 55202 | + |  |  |  |  |  |
| 18 | *Tf*-Manna-Li | Li-H | Doc-*Ct*II | pVTD3 | 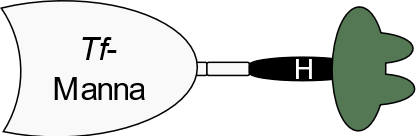 | 54631 | + |  |  |  |  |  |
| 19 | *Tf*-Manna | Doc-*Ac* | His | pVTD2 | 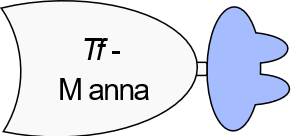 | 43438 | ++ |  |  |  |  |  |
| 20 | *Tf*-Manna | Li-E | Doc-*Ac* | pVTD3 | 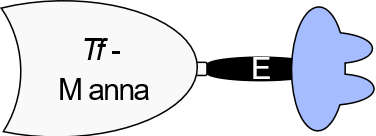 | 44402 | ++ |  |  |  |  |  |
| 21 | *Tf*-Manna | Li-F | Doc-*Ac* | pVTD3 | 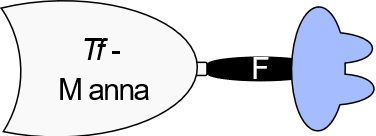 | 43913 | ++ |  |  |  |  |  |
| 22 | *Tf*-Manna | Li-G | Doc-*Ac* | pVTD3 | 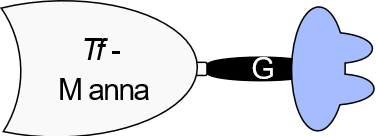 | 44868 | ++ |  |  |  |  |  |
| 23 | *Tf*-Manna | Li-H | Doc-*Ac* | pVTD3 | 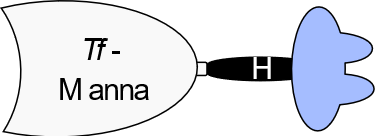 | 44297 | ++ |  |  |  |  |  |
| 24 | *Tf*-Manna-Li | Li-E | Doc-*Ac* | pVTD3 | 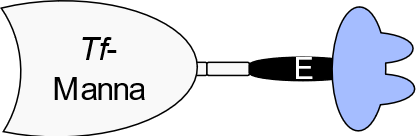 | 47029 | ++ |  |  |  |  |  |
| 25 | *Tf*-Manna-Li | Li-F | Doc-*Ac* | pVTD3 | 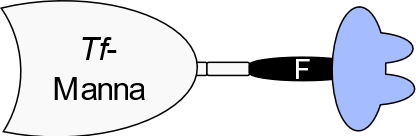 | 46539 | ++ |  |  |  |  |  |
| 26 | *Tf*-Manna-Li | Li-G | Doc-*Ac* | pVTD3 | 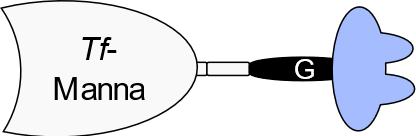 | 47494 | ++ |  |  |  |  |  |
| 27 | *Tf*-Manna-Li | Li-H | Doc-*Ac* | pVTD3 | 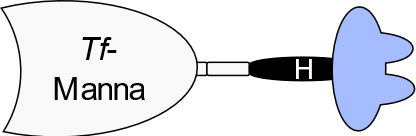 | 46923 | ++ |  |  |  |  |  |
| 28 | Doc-*Rf* | *Tf*-Manna-Li | His | pVTD2 | 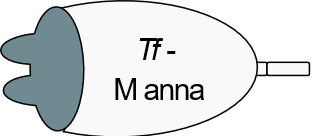 | 46871 | ++ |  |  |  |  |  |
| 29 | Doc-*Cc* | *Tf*-Manna-Li | His | pVTD2 | 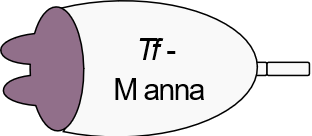 | 45596 | ++ |  |  |  |  |  |
| 30 | Doc-*Cc* | *Tf*-Manna-S | His | pVTD2 | 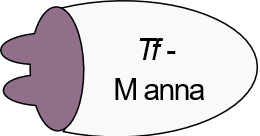 | 40348 | +++ |  |  |  |  |  |
| 31 | Doc-*Cc* | *Tf*-Manna-CBM | His | pVTD2 | 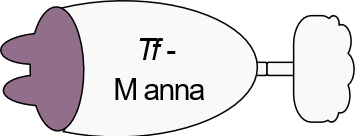 | 55200 | ++ |  |  |  |  |  |
|  | **Monocatalytic: Mannosidase** | | | | | | |  |  |  |  | ++ |
| 32 | *Tf*-Manno | His | | pVTD2 | 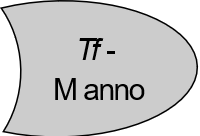 | 95585 | ++ |  |  |  |  |  |
| 33 | *Tf*-Manno | Doc-*Ct*I | His | pVTD2 | 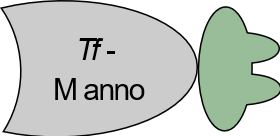 | 103823 | ++ |  |  |  |  |  |
| 34 | His | Doc-*Ct*I | *Tf*-Manno | pVTD2 | 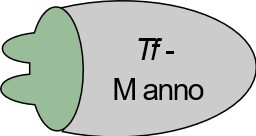 | 103823 | +++ |  |  |  |  |  |
| 35 | *Tf*-Manno | Doc-*Cc* | His | pVTD2 | 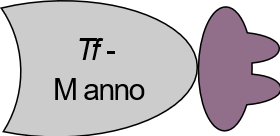 | 103117 | ++ |  |  |  |  |  |
| 36 | *Tf*-Manno | Doc-*Ct*II | His | pVTD2 | 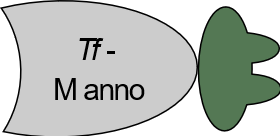 | 112116 | - |  |  |  |  |  |
| 37 | *Tf*-Manno | Doc-*Ac* | His | pVTD2 | 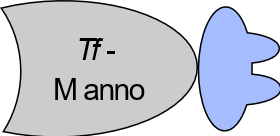 | 104408 | ++ |  |  |  |  |  |
| 38 | *Tf*-Manno | Doc-*Af* | His | pVTD2 | 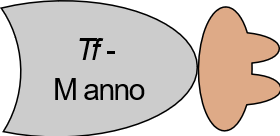 | 103900 | - |  |  |  |  |  |
| 39 | *Tf*-Manno | Doc-*Bc* | His | pVTD2 | 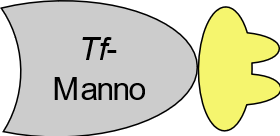 | 104353 | ++ |  |  |  |  |  |
|  | **Monocatalytic: Galactosidase** | | | | | | |  |  |  |  |  |
| 40 | *Cc*-Aga | His | | pVTD2 | 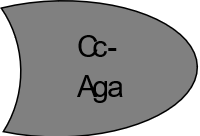 | 38834 | - |  |  |  |  |  |
| 41 | *Cc*-Aga-CBM | His | | pVTD2 | 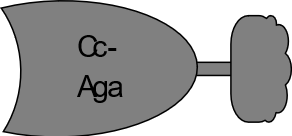 | 52555 | ++ |  |  |  |  |  |
| 42 | *Cc*-Aga-Li | Doc-*Ct*I | His | pVTD2 | 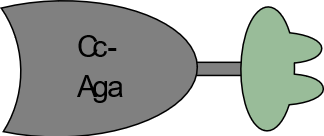 | 50942 | ++ |  |  |  |  |  |
| 43 | His | Doc-*Ct*I | *Cc*-Aga-Li | pVTD2 | 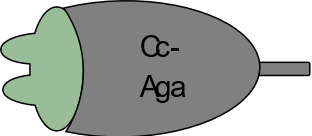 | 50942 | - |  |  |  |  |  |
| 44 | *Cc*-Aga-CBM | Doc-*Ct*I | His | pVTD2 | 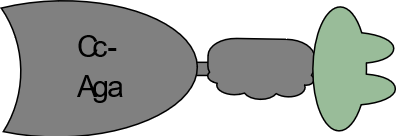 | 60420 | ++ |  |  |  |  |  |
| 45 | *Cc*-Aga-Li | Doc-*Ct*II | His | pVTD2 | 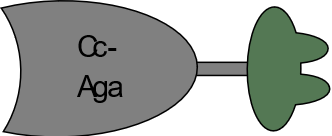 | 59235 | + |  |  |  |  |  |
| 46 | Doc-*Ct*I | *Cc*-Aga-Li | His | pVTD2 | 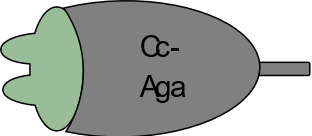 | 51765 | + |  |  |  |  |  |
| 47 | Doc-*Ct*I | *Cc*-Aga-CBM | His | pVTD2 | 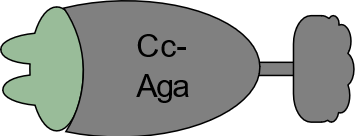 | 61617 | - |  |  |  |  |  |
| 48 | Doc-*Ct*I | *Cc*-Aga | His | pVTD2 | 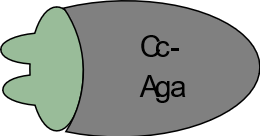 | 47895 | - |  |  |  |  |  |
| 49 | *Ba*-Aga | His | | pVTD2 | 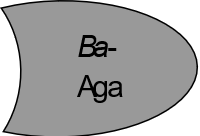 | 85415 | - |  |  |  |  |  |
| 50 | *Ba*-Aga | Doc-*Ct*II | His | pVTD2 | 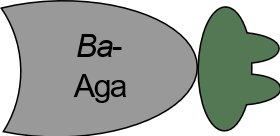 | 101946 | - |  |  |  |  |  |
| 51 | *Ba*-Aga | Doc-*Ct*I | His | pVTD2 | 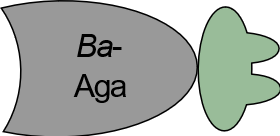 | 93653 | - |  |  |  |  |  |
| 52 | *Ba*-Aga | Dock -*Rf* | His | pVTD2 | 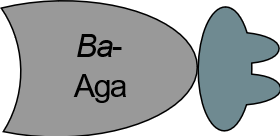 | 94221 | - |  |  |  |  |  |
| 53 | *Ba*-Aga | Doc-*Cc* | His | pVTD2 | 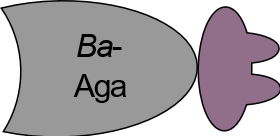 | 92947 | - |  |  |  |  |  |
| 54 | Doc-*Ct*I | *Ba*-Aga | His | pVTD2 | 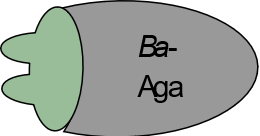 | 94345 | ++ |  |  |  |  |  |
| 55 | *Cj*-Aga | His | | pVTD2 | 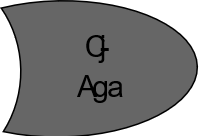 | 44940 | +++ |  |  |  |  |  |
| 56 | *Cj*-Aga | Doc-*Ct*I | His | pVTD2 | 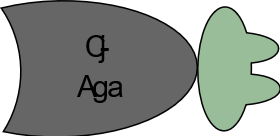 | 53178 | + |  |  |  |  |  |
| 57 | *Cj*-Aga | Doc-*Ct*II | His | pVTD2 | 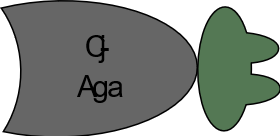 | 61470 | - |  |  |  |  |  |
| 58 | *Cj*-Aga | Doc-*Cc* | His | pVTD2 | 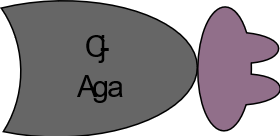 | 52471 | + |  |  |  |  |  |
| 59 | *Cj*-Aga | Doc-*Rf* | His | pVTD2 |  | 53745 | - |  |  |  |  |  |
| 60 | *Cj*-Aga | Li-A | Doc-*Ct*I | pVTD3 |  | 54297 | ++ |  |  |  |  |  |
| 61 | *Cj*-Aga | Li-B | Doc-*Ct*I | pVTD3 |  | 54899 | ++ |  |  |  |  |  |
| 62 | *Cj*-Aga | Li-C | Doc-*Ct*I | pVTD3 |  | 54929 | +++ |  |  |  |  |  |
| 63 | *Cj*-Aga | Li-D | Doc-*Ct*I | pVTD3 |  | 53754 | +++ |  |  |  |  |  |
|  | **Bicatalytic: Mannanase + Mannosidase** | | | | | | |  |  |  |  |  |
| 64 | *Tf*-Manno | Doc-*Ct*I | *Tf*-Manna-Li | pVTD3 |  | 139676 | ++ |  |  |  |  |  |
| 65 | *Tf*-Manna-Li | Doc-*Ct*I | *Tf*-Manno | pVTD3 |  | 139676 | + |  |  |  |  |  |
| 66 | *Tf*-Manno | *Tf*-Manna-Li | Doc-*Ct*I | pVTD3 |  | 139676 | +++ |  |  |  |  |  |
| 67 | *Tf*-Manna-Li | *Tf*-Manno | Doc-*Ct*I | pVTD3 |  | 139676 | - |  |  |  |  |  |
| 68 | Doc-*Ct*I | *Tf*-Manna-Li | *Tf*-Manno | pVTD1 |  | 139676 | ++ |  |  |  |  |  |
| 69 | Doc-*Ct*I | *Tf*-Manno | *Tf*-Manna-Li | pVTD1 |  | 139676 | ++ |  |  |  |  |  |
| 70 | *Tf*-Manno | *Tf*-Manna-Li | Doc-*Rf* | pVTD3 |  | 140225 | ++ |  |  |  |  |  |
| 71 | *Tf*-Manno | Doc-*Rf* | *Tf*-Manna-Li | pVTD3 |  | 140225 | ++ |  |  |  |  |  |
| 72 | *Tf*-Manno | Doc-*Cc* | *Tf*-Manna-Li | pVTD3 |  | 138951 | ++ |  |  |  |  |  |
|  | **Bicatalytic: Mannanase + Galactosidase** | | | | | | |  |  |  |  |  |
| 73 | Doc-*Cc* | *Tf*-Manna-Li | *Cc*-Aga-Li | pVTD3 |  | 86070 | ++ |  |  |  |  |  |
| 74 | Doc-*Ct*I | *Tf*-Manna-Li | *Ba*-Aga | pVTD3 |  | 129487 | ++ |  |  |  |  |  |
| 75 | *Tf*-Manna-Li | *Cc*-Aga-Li | Doc-*Cc* | pVTD3 |  | 86070 | - |  |  |  |  |  |
| 76 | *Cc-Aga-*Li | *Tf*-Manna-Li | Doc-*Cc* | pVTD3 |  | 86070 | - |  |  |  |  |  |
| 77 | *Tf*-Manna-Li | Doc-*Cc* | *Cc*-Aga-Li | pVTD3 |  | 86070 | ND |  |  |  |  |  |
| 78 | *Cc*-Aga-Li | Doc-*Cc* | *Tf*-Manna-Li | pVTD3 |  | 86070 | ++ |  |  |  |  |  |
| 79 | Doc-*Cc* | *Cc*-Aga-Li | *Tf*-Manna-Li | pVTD3 |  | 86070 | ND |  |  |  |  |  |

**Table S8: Scaffoldin variants constructed in this study.** The first column shows the number of the construct. The second, third, fourth, fifth and sixth columns show the tiles that were used in the assembly reaction. The scaffoldin assembly system allows the assembly of five tiles. However, His-tag tiles overlapping multiple positions allow the assembly of four, three or two tiles. The seventh column indicates the destination vector in which the tiles were assembled. Note that pVTD2 was used when the His-tag sequence was added as a tile. When all positions were occupied by other tiles, other destination vectors were employed. pVTD10 allowed the fusion of an N-terminal His- and GST-tag. Assembling in pVTD13 resulted in the fusion of an N-terminal GST-tag and a C-terminal His-tag and use of pVTD17 ensured the fusion of an N-terminal GST-tag and CBM and a C-terminal His-tag (see **Figure S6:**). The eighth and ninth columns show the icon (see **Table S3** and **Table S4**) and molecular weight (MW) of the resulting protein. The final column indicates the expression efficiency of each scaffoldin. The semi-quantitative score is identical to **Table S7:**. ND = not determined.

| **Nr.** | **Tile 1** | **Tile 2** | **Tile 3** | **Tile 4** | | **Tile 5** | **Vector** | **Icon** | **MW (Da)** | **Expression** |
| --- | --- | --- | --- | --- | --- | --- | --- | --- | --- | --- |
| 80 | *Ct*-CBM3 | Coh-*Ct*I | His | | | | pVTD2 |  | 37970 | +++ |
| 81 | *Ct*-CBM3 | Coh-*Ct*II | His | | | | pVTD2 |  | 38758 | +++ |
| 82 | *Ct*-CBM3 | Coh-*Rf* | His | | | | pVTD2 |  | 37182 | +++ |
| 83 | *Ct*-CBM3 | Coh-*Cc* | His | | | | pVTD2 |  | 34130 | +++ |
| 84 | *Ct*-CBM3 | Coh-*Ac* | His | | | | pVTD2 |  | 34878 | ++ |
| 85 | *Ct*-CBM3 | Coh-*Af* | His | | | | pVTD2 |  | 36255 | ++ |
| 86 | *Ct*-CBM3 | Coh-*Bc* | His | | | | pVTD2 |  | 35352 | +++ |
| 87 | *Ct*-CBM3 | Coh-*Ct*I | Coh-*Cc* | Coh-*Rf* | Coh-*Ct*II | | pVTD3 |  | 94243 | +++ |
| 88 | *Ct*-CBM3 | Coh-*Ct*I | Coh-*Cc* | His | | | pVTD2 |  | 56838 | +++ |
| 89 | *Ct*-CBM3 | Coh-*Cc* | Coh-*Ct*I | His | | | pVTD2 |  | 56838 | +++ |
| 90 | *Ct*-CBM3 | Coh-*Ct*I | Coh-*Cc* | Coh-*Ct*II | His | | pVTD2 |  | 76796 | ND |
| 91 | *Ct*-CBM3 | Coh-*Ct*I | Coh-*Ct*II | Coh-*Cc* | His | | pVTD2 |  | 76796 | ND |
| 92 | *Ct*-CBM3 | Coh-*Cc* | Coh-*Ct*I | Coh-*Ct*II | His | | pVTD2 |  | 76796 | ND |
| 93 | *Ct*-CBM3 | Coh-*Cc* | Coh-*Ct*II | Coh-*Ct*I | His | | pVTD2 |  | 76796 | ND |
| 94 | *Ct*-CBM3 | Coh-*Ct*II | Coh-*Cc* | Coh-*Ct*I | His | | pVTD2 |  | 76796 | ND |
| 95 | *Ct*-CBM3 | Coh-*Ct*II | Coh-*Ct*I | Coh-*Cc* | His | | pVTD2 |  | 76796 | ND |
| 96 | *Ct*-CBM3 | Coh-*Ct*I | Coh-*Ct*II | His | | | pVTD2 |  | 61467 | - |
| 97 | *Ct*-CBM3 | Coh-*Ct*II | Coh-*Ct*I | His | | | pVTD2 |  | 61467 | - |
| 98 | His | *Ct*-CBM3 | Coh-*Ct*I | Coh-*Cc* | Coh-*Ct*II | | pVTD2 |  | 76129 | - |
| 99 | His | *Ct*-CBM3 | Coh-*Ct*I | Coh-*Ct*II | Coh-*Cc* | | pVTD2 |  | 75628 | +++ |
| 100 | His | *Ct*-CBM3 | Coh-*Cc* | Coh-*Ct*II | Coh-*Ct*I | | pVTD2 |  | 72763 | +++ |
| 101 | His | *Ct*-CBM3 | Coh-*Cc* | Coh-*Ct*I | Coh-*Ct*II | | pVTD2 |  | 76129 | - |
| 102 | His | *Ct*-CBM3 | Coh-*Ct*II | Coh-*Ct*I | Coh-*Cc* | | pVTD2 |  | 75729 | +++ |
| 103 | His | *Ct*-CBM3 | Coh-*Ct*II | Coh-*Cc* | Coh-*Ct*I | | pVTD2 |  | 72762 | +++ |
| 104 | His | *Cj*-CBM35 | Coh-*Ct*I | Coh-*Cc* | Coh-*Ct*II | | pVTD2 |  | 68816 | - |
| 105 | His | *Cj*-CBM35 | Coh-*Ct*I | Coh-*Ct*II | Coh-*Cc* | | pVTD2 |  | 69203 | ND |
| 106 | His | *Cj*-CBM35 | Coh-*Cc* | Coh-*Ct*II | Coh-*Ct*I | | pVTD2 |  | 66338 | ND |
| 107 | His | *Cj*-CBM35 | Coh-*Cc* | Coh-*Ct*I | Coh-*Ct*II | | pVTD2 |  | 69603 | ND |
| 108 | His | *Cj*-CBM35 | Coh-*Ct*II | Coh-*Ct*I | Coh-*Cc* | | pVTD2 |  | 69203 | ND |
| 109 | His | *Cj*-CBM35 | Coh-*Ct*II | Coh-*Cc* | Coh-*Ct*I | | pVTD2 |  | 66338 | ND |
| 110 | His | Coh-*Ct*I | Coh-*Cc* | Coh-*Ct*II | *Cj*-CBM35 | | pVTD2 |  | 69583 | - |
| 111 | His | Coh-*Ct*I | Coh-*Ct*II | Coh-*Cc* | *Cj*-CBM35 | | pVTD2 |  | 69583 | ND |
| 112 | His | Coh-*Cc* | Coh-*Ct*II | Coh-*Ct*I | *Cj*-CBM35 | | pVTD2 |  | 69583 | ND |
| 113 | His | Coh-*Cc* | Coh-*Ct*I | Coh-*Ct*II | *Cj*-CBM35 | | pVTD2 |  | 69583 | ND |
| 114 | His | Coh-*Ct*II | Coh-*Ct*I | Coh-*Cc* | *Cj*-CBM35 | | pVTD2 |  | 69583 | ND |
| 115 | His | Coh-*Ct*II | Coh-*Cc* | Coh-*Ct*I | *Cj*-CBM35 | | pVTD2 |  | 69583 | ND |
| 116 | *Ct*-CBM3 | Coh-*Ct*II | Coh-*Bc* | Coh-*Ct*I | His | | pVTD2 |  | 78018 | +++ |
| 117 | His | *Ct*-CBM3 | Coh-*Ct*II | Coh-*Bc* | Coh-*Ct*I | | pVTD2 |  | 74148 | ++ |
| 118 | *Ct*-CBM3 | Coh-*Ct*II | Coh-*Bc* | Coh-*Ct*I | His | | pVTD5 |  | 80280 | +++ |
| 119 | His_GST | *Ct*-CBM3 | Coh-*Ct*II | Coh-*Bc* | Coh-*Ct*I | | pVTD2 |  | 100667 | ++ |
| 120 | GST | *Ct*-CBM3 | Coh-*Ct*II | Coh-*Bc* | Coh-*Ct*I | | pVTD3 |  | 100501 | ++ |
| 121 | StrepTagII | *Ct*-CBM3 | Coh-*Ct*II | Coh-*Bc* | Coh-*Ct*I | | pVTD3 |  | 75075 | ND |
| 122 | StrepTagII | *Ct*-CBM3 | Coh-*Ct*II | Coh-*Bc* | Coh-*Ct*I | | pVTD5 |  | 75424 | ND |
| 123 | His_GST | *Ct*-CBM3 | Coh-*Ct*I | Coh-*Bc* | Coh-*Ac* | | pVTD2 |  | 100209 | ++ |
| 124 | GST | *Ct*-CBM3 | Coh-*Ct*I | Coh-*Bc* | Coh-*Ac* | | pVTD3 |  | 100043 | ++ |
| 125 | StrepTagII | *Ct*-CBM3 | Coh-*Ct*I | Coh-*Bc* | Coh-*Ac* | | pVTD3 |  | 74748 | ND |
| 126 | StrepTagII | *Ct*-CBM3 | Coh-*Ct*I | Coh-*Bc* | Coh-*Ac* | | pVTD5 |  | 74965 | ND |
| 127 | His_GST | *Ct*-CBM3 | Coh-*Ct*II | Coh-*Ct*I | Coh-*Bc* | | pVTD2 |  | 104186 | ++ |
| 128 | His_GST | *Ct*-CBM3 | Coh-*Bc* | Coh-*Ct*II | Coh-*Ct*I | | pVTD2 |  | 100667 | ++ |
| 129 | His_GST | *Ct*-CBM3 | Coh-*Ct*I | Coh-*Ct*II | Coh-*Bc* | | pVTD2 |  | 104186 | ++ |
| 130 | His_GST | *Ct*-CBM3 | Coh-*Bc* | Coh-*Ct*I | Coh-*Ct*II | | pVTD2 |  | 103932 | ++ |
| 131 | His_GST | *Ct*-CBM3 | Coh-*Ct*I | Coh-*Bc* | Coh-*Ct*II | | pVTD2 |  | 103932 | ++ |
| 132 | His_GST | *Cj*-CBM35 | Coh-*Ct*II | Coh-*Bc* | Coh-*Ct*I | | pVTD2 |  | 94141 | ++ |
| 133 | His_GST | *Ct*-CBM3 | Coh-*Ac* | Coh-*Bc* | Coh-*Ct*I | | pVTD2 |  | 96787 | ++ |
| 134 | His_GST | *Cj*-CBM35 | Coh-*Ac* | Coh-*Bc* | Coh-*Ct*I | | pVTD2 |  | 90261 | ++ |
| 135 | GST | *Ct*-CBM3 | Coh-*Ac* | Coh-*Bc* | Coh-*Ct*I | | pVTD3 |  | 96787 | ++ |
| 136 | GST | *Ct*-CBM3 | Coh-*Ac* | Coh-*Ct*I | Coh-*Bc* | | pVTD3 |  | 96787 | ++ |
| 137 | GST | *Ct*-CBM3 | Coh-*Bc* | Coh-*Ac* | Coh-*Ct*I | | pVTD3 |  | 96787 | ++ |
| 138 | GST | *Ct*-CBM3 | Coh-*Ct*I | Coh-*Ac* | Coh-*Bc* | | pVTD3 |  | 96787 | ++ |
| 139 | GST | *Ct*-CBM3 | Coh-*Bc* | Coh-*Ct*I | Coh-*Ac* | | pVTD3 |  | 96787 | ++ |
| 140 | GST | *Ct*-CBM3 | Coh-*Ct*I | Coh-*Bc* | Coh-*Ac* | | pVTD3 |  | 96787 | ++ |
| 141 | GST | *Cj*-CBM35 | Coh-*Ac* | Coh-*Bc* | Coh-*Ct*I | | pVTD3 |  | 90261 | - |
| 142 | GST | *Cj*-CBM35 | Coh-*Ac* | Coh-*Ct*I | Coh-*Bc* | | pVTD3 |  | 90261 | ++ |
| 143 | GST | *Cj*-CBM35 | Coh-*Bc* | Coh-*Ac* | Coh-*Ct*I | | pVTD3 |  | 90261 | ++ |
| 144 | GST | *Cj*-CBM35 | Coh-*Ct*I | Coh-*Ac* | Coh-*Bc* | | pVTD3 |  | 90261 | ++ |
| 145 | GST | *Cj*-CBM35 | Coh-*Bc* | Coh-*Ct*I | Coh-*Ac* | | pVTD3 |  | 90261 | - |
| 146 | GST | *Cj*-CBM35 | Coh-*Ct*I | Coh-*Bc* | Coh-*Ac* | | pVTD3 |  | 90261 | ++ |
| 147 | *Ct*-CBM3 | Coh-*Ac* | Coh-*Bc* | Coh-*Ct*I | Coh-*Ct*I | | pVTD13 |  | 111971 | ++ |
| 148 | *Cj*-CBM35 | Coh-*Ac* | Coh-*Bc* | Coh-*Ct*I | Coh-*Ct*I | | pVTD13 |  | 105445 | ++ |
| 149 | Coh-*Ac* | Coh-*Bc* | Coh-*Ct*I | Coh-*Ct*I | Coh-*Ct*I | | pVTD17 |  | 132251 | ++ |
| 150 | *Ct*-CBM3 | Coh-*Ac* | Coh-*Ac* | Coh-*Bc* | Coh-*Ct*I | | pVTD13 |  | 112913 | ++ |
| 151 | Coh-*Ac* | Coh-*Ac* | Coh-*Ac* | Coh-*Bc* | Coh-*Ct*I | | pVTD17 |  | 128995 | ++ |

**Figure S7: Influence of dockerin position on the expression and purification yield of mannanase docking enzymes.** Left: purification of *Tf*-Manna-Li_Doc-*Ct*I (construct nr. 4, 45 kDa). Right: purification of Doc-*Ct*I_*Tf*-Manna-Li (construct nr. 5, 45 kDa). Samples are loaded in the same order for both gels. Lane 1: Marker, Lane 2: Flow-through, Lane 3: Wash, Lanes 4, 5, 6, 7, 8 and 9: Elution fractions 1-6.

**Figure S8: Influence of dockerin position on the expression and purification yield of mannosidase docking enzymes.** Left: purification of *Tf*-Manno_Doc-*Ct*I (construct nr. 33, 104 kDa). Right: purification of Doc-*Ct*I_*Tf*-Manno (construct nr. 34, 104 kDa). Samples are loaded in the same order for both gels. Lane 1: Flow-through, Lane 2: Wash, Lanes 3, 4, 5, 6, 7 and 8: Elution fractions 1-6, Lane 9: Marker.

**Figure S9: Influence of dockerin position on the expression and purification of galactosidase docking enzymes.** Left: purification of *Cc*-Aga-Li_Doc-*Ct*I (construct nr. 42, 51 kDa). Middle: purification of Doc-*Ct*I_*Cc*-Aga-Li (construct nr. 43, 51 kDa). Right: purification of *Cc*-Aga-CBM_Doc-*Ct*I (construct nr. 44, 60 kDa). Samples are loaded in the same order for all gels. Lane 1: Marker, Lane 2: Flow-through, Lane 3: Wash, Lanes 4, 5, 6, 7, 8 and 9: Elution fractions 1-6.

**Figure S10: SDS-PAGE analysis of fractions obtained after GST pull-down (1).** Left gel: Lane 1: Marker, Lanes 2, 3, 4, 5, 6, 7 and 8: GST-mediated isolation of complex composed of *Ct*-CBM3_Coh-*Ac*_Coh-*Bc*_Coh-*Ct*I (construct nr. 135, 97 kDa, purple arrow) and *Tf*-Manna_Li-G_Doc-*Ac* (construct nr. 26, 47 kDa, white arrow). Lanes 9, 10, 11, 12, 13, 14 and 15: GST pull-down of complex composed of *Ct*-CBM3_Coh-*Ac*_Coh-*Bc*_Coh-*Ct*I (construct nr. 135, 97 kDa, purple arrow) and *Tf*_Manno_Doc-*Bc* (construct nr. 39, 104 kDa, light grey arrow). Right gel: Lane 1: Marker, Lanes 2, 3, 4, 5, 6, 7, and 8: GST pull-down of complex composed *Ct*-CBM3_Coh-*Ac*_Coh-*Bc*_Coh-*Ct*I (construct nr. 135, 97 kDa, purple arrow) and *Cj*-Aga_Li-D_Doc-*Ct*I (construct nr. 63, 54 kDa, dark grey arrow). Lanes 9, 10, 11, 12, 13, 14 and 15: GST pull-down of complex composed of *Ct*-CBM3_Coh-*Ac*_Coh-*Bc*_Coh-*Ct*I (construct nr. 135, 97 kDa, purple arrow), *Tf*-Manna_Li-G_Doc-*Ac* (construct nr. 26, 47 kDa, white arrow) and *Tf*-Manno_Doc-*Bc* (construct nr. 39, 104 kDa, light grey arrow). The flow-through (FT), three wash fractions (W1, W2 and W3) and three elution fractions (E1, E2 and E3) are loaded in respective lanes. We note that whenever *Cj*-Aga_Li-D_Doc-*Ct*I is included in the designer cellulosome, an additional band can be detected. We hypothesise that this band corresponds to a degradation product of the galactosidase docking enzyme that is able to interact with the scaffoldin.

**Figure S11: SDS-PAGE analysis of fractions obtained after GST pull-down (2).** Left gel: Lane 1: Marker, Lanes 2, 3, 4, 5, 6, 7 and 8: GST-mediated isolation of complex composed of *Ct*-CBM3_Coh-*Ac*_Coh-*Bc*_Coh-*Ct*I (construct nr. 135, 97 kDa, purple arrow), *Tf*-Manna_Li-G_Doc-*Ac* (construct nr. 26, 47 kDa, white arrow) and *Cj*-Aga_Li-D_Doc-*Ct*I (construct nr. 63, 54 kDa, dark grey arrow). Lanes 9, 10, 11, 12, 13, 14 and 15: GST pull-down of complex composed of *Ct*-CBM3_Coh-*Ac*_Coh-*Bc*_Coh-*Ct*I (construct nr. 135, 97 kDa, purple arrow), *Tf*-Manno_Doc-*Bc* (construct nr. 39, 104 kDa, light grey arrow) and *Cj*-Aga_Li-D_Doc-*Ct*I (construct nr. 63, 54 kDa, dark grey arrow). Right gel: Lane 1: Marker, Lanes 2, 3, 4, 5, 6, 7, and 8: GST pull-down of complex composed *Ct*-CBM3_Coh-*Ac*_Coh-*Bc*_Coh-*Ct*I (construct nr. 135, 97 kDa, purple arrow), *Tf*-Manna_Li-G_Doc-*Ac* (construct nr. 26, 47 kDa, white arrow), *Tf*-Manno_Doc-*Bc* (construct nr. 39, 104 kDa, light grey arrow) and *Cj*-Aga_Li-D_Doc-*Ct*I (construct nr. 63, 54 kDa, dark grey arrow). The flow-through (FT), three wash fractions (W1, W2 and W3) and three elution fractions (E1, E2 and E3) are loaded in respective lanes. We note that whenever *Cj*-Aga_Li-D_Doc-*Ct*I is included in the designer cellulosome, an additional band can be detected. We hypothesise that this band corresponds to a degradation product of the galactosidase docking enzyme that is able to interact with the scaffoldin.

**Figure S12: SDS-PAGE analysis of fractions obtained after GST pull-down (3).** Left gel: Lane 1: Marker, Lanes 2, 3, 4, 5, 6, 7 and 8: GST-mediated isolation of complex composed of *Ct*-CBM3_Coh-*Ac*_Coh-*Bc*_Coh-*Ct*I (construct nr. 135, 97 kDa, purple arrow), *Tf*-Manna_Li-G_Doc-*Ac* (construct nr. 26, 47 kDa, white arrow), *Tf*-Manno_Doc-*Bc* (construct nr. 39, 104 kDa, light grey arrow) and *Cj*-Aga_Li-D_Doc-*Ct*I (construct nr. 63, 54 kDa, dark grey arrow). Lanes 9, 10, 11, 12, 13, 14 and 15: GST pull-down of complex composed of *Ct*-CBM3_Coh-*Ac*_Coh-*Ct*I_Coh-*Bc* (construct nr. 136, 97 kDa, purple arrow), *Tf*-Manna_Li-G_Doc-*Ac* (construct nr. 26, 47 kDa, white arrow), *Tf*-Manno_Doc-*Bc* (construct nr. 39, 104 kDa, light grey arrow) and *Cj*-Aga_Li-D_Doc-*Ct*I (construct nr. 63, 54 kDa, dark grey arrow). Right gel: Lane 1: Marker, Lanes 2, 3, 4, 5, 6, 7, and 8: GST pull-down of complex composed *Ct*-CBM3_Coh- *Bc*_Coh-*Ac*_Coh-*Ct*I (construct nr. 137, 97 kDa, purple arrow), *Tf*-Manna_Li-G_Doc-*Ac* (construct nr. 26, 47 kDa, white arrow), *Tf*-Manno_Doc-*Bc* (construct nr. 39, 104 kDa, light grey arrow) and *Cj*-Aga_Li-D_Doc-*Ct*I (construct nr. 63, 54 kDa, dark grey arrow). Lanes 9, 10, 11, 12, 13, 14 and 15: GST-mediated isolation of complex composed of *Ct*-CBM3_Coh-*Ct*I_Coh-*Ac*_Coh-*Bc* (construct nr. 138, 97 kDa, purple arrow), *Tf*-Manna_Li-G_Doc-*Ac* (construct nr. 26, 47 kDa, white arrow), *Tf*-Manno_Doc-*Bc* (construct nr. 39, 104 kDa, light grey arrow) and *Cj*-Aga_Li-D_Doc-*Ct*I (construct nr. 63, 54 kDa, dark grey arrow). The flow-through (FT), three wash fractions (W1, W2 and W3) and three elution fractions (E1, E2 and E3) are loaded in respective lanes.

**Figure S13: SDS-PAGE analysis of fractions obtained after GST pull-down (4).** Lane 1: Marker, Lanes 2, 3, 4, 5, 6, 7 and 8: GST-mediated isolation of complex composed of *Ct*-CBM3_Coh-*Bc*_Coh-*Ct*I_Coh-*Ac* (construct nr. 139, 97 kDa, purple arrow), *Tf*-Manna_Li-G_Doc-*Ac* (construct nr. 26, 47 kDa, white arrow), *Tf*-Manno_Doc-*Bc* (construct nr. 39, 104 kDa, light grey arrow) and *Cj*-Aga_Li-D_Doc-*Ct*I (construct nr. 63, 54 kDa, dark grey arrow). Lanes 9, 10, 11, 12, 13, 14 and 15: GST pull-down of complex composed of *Ct*-CBM3_Coh-*Ct*I_Coh-*Bc*_Coh-*Ac* (construct nr. 140, 97 kDa, purple arrow), *Tf*-Manna_Li-G_Doc-*Ac* (construct nr. 26, 47 kDa, white arrow), *Tf*-Manno_Doc-*Bc* (construct nr. 39, 104 kDa, light grey arrow) and *Cj*-Aga_Li-D_Doc-*Ct*I (construct nr. 63, 54 kDa, dark grey arrow).

**Figure S14: SDS-PAGE of fractions obtained after GST pull-down (5).** Left gel: Lane 1: Marker, Lanes 2, 3, 4, 5 and 6: GST-mediated isolation of complex composed of *Ct*-CBM3_Coh-*Ac*_Coh-*Bc*_Coh-*Ct*I (construct nr. 135, 97 kDa, purple arrow), *Tf*-Manna_Li-G_Doc-*Ac* (construct nr. 26, 47 kDa, white arrow), *Tf*-Manno_Doc-*Bc* (construct nr. 39, 104 kDa, light grey arrow) and *Cj*-Aga_Li-D_Doc-*Ct*I (construct nr. 63, 54 kDa, dark grey arrow). Right gel: Lane 1: Marker, Lanes 2, 3, 4, 5 and 6: GST-mediated isolation of complex composed of *Ct*-CBM3_Coh-*Ac*_Coh-*Ac*_Coh-*Bc*_Coh-*Ct*I (construct nr. 150, 113 kDa, orange arrow), *Tf*-Manna_Li-G_Doc-*Ac* (construct nr. 26, 47 kDa, white arrow), *Tf*-Manno_Doc-*Bc* (construct nr. 39, 104 kDa, light grey arrow) and *Cj*-Aga_Li-D_Doc-*Ct*I (construct nr. 63, 54 kDa, dark grey arrow). Lanes 7, 8, 9, 10 and 11: GST-mediated isolation of complex composed of *Ct*-CBM3_Coh-*Ac*_Coh-*Ac*_Coh-*Ac*_Coh-*Bc*_Coh-*Ct*I (construct nr. 151, 129 kDa, red arrow), *Tf*-Manna_Li-G_Doc-*Ac* (construct nr. 26, 47 kDa, white arrow), *Tf*-Manno_Doc-*Bc* (construct nr. 39, 104 kDa, light grey arrow) and *Cj*-Aga_Li-D_Doc-*Ct*I (construct nr. 63, 54 kDa, dark grey arrow).

**Table S9: Overview of primers used to amplify dockerin tiles.** The first column shows the position of the tile in the final construct. The second column gives the name of the tile, and the third column indicates the orientation of the primer. In the following columns, each section of the primer is shown starting from the 5’ end. All primers consist of three extra nucleotides for clamping of the restriction enzyme, a restriction/recognition site of a specific restriction enzyme, the BsaI recognition site, followed by the position tag and a sequence that is complementary with the fragment of interest.

| **Position** | **Tile** | **Primer** | **3 extra nt** | **Recognition site (for HindIII, BamHI, XbaI, SalI) or recognition (SapI) and restriction site** | **BsaI recognition site** | **Position tag (DE_s_, DE_2_, DE_3_, DE_e_)** | **Complementary sequence that hybridises with template** |
| --- | --- | --- | --- | --- | --- | --- | --- |
| 1 | DE_s__Doc-*Ct*I_DE_2_ | Forward | ATA | TCTAGA | GGTCTC | ACCATG | GTACCTGGTACTCCTTCTAC |
|  |  | Reverse | ATA | AAGCTT | GGTCTC | TGAACC | GTTCTTGTACGGCAATGT |
|  | DE_s__Doc-*Ct*NI_DE_2_ | Forward | ATA | GCTCTTCAAGA | GGTCTC | ACCATG | GAAAGCAGTTCCACAGGTCTGG |
|  |  | Reverse | ATA | GCTCTTCACTT | GGTCTC | TGAACC | ATTTCCCGAAATCGTGGGGATAGG |
|  | DE_s__Doc-*Ct*II_DE_2_ | Forward | ATA | TCTAGA | GGTCTC | ACCATG | GGATACATTTTGCCAGACTTC |
|  |  | Reverse | ATA | AAGCTT | GGTCTC | TGAACC | CTGTGCGTCGTAATCACTTG |
|  | DE_s__Doc-*Rf*_DE_2_ | Forward | ATA | GGATCC | GGTCTC | ACCATG | GATAAGGATGTAGTCTAC |
|  |  | Reverse | ATA | GTCGAC | GGTCTC | TGAACC | TTCGGGAAGCTTGTCTATGAGATG |
|  | DE_s__Doc-*Cc*_DE_2_ | Forward | ATA | GGATCC | GGTCTC | ACCATG | CCAGTAATTGTATATGG |
|  |  | Reverse | ATA | GTCGAC | GGTCTC | TGAACC | GTTGCTTGGAAGCTTA |
|  | DE_s__Doc-*Ac*_DE_2_ | Gene fragment was ordered (Twist Bioscience) | | | | | |
|  |  |  |  |  |  |  |  |
|  | DE_s__Doc-*Bc*_DE_2_ | Gene fragment was ordered (Twist Bioscience) | | | | | |
|  |  |  |  |  |  |  |  |
|  | DE_s__Doc-*Af*_DE_2_ | Gene fragment was ordered (Twist Bioscience) | | | | | |
|  |  |  |  |  |  |  |  |
| 2 | DE_2__Doc-*Ct*I_DE_3_ | Forward | ATA | TCTAGA | GGTCTC | GGTTCA | GTACCTGGTACTCCTTCTAC |
|  |  | Reverse | ATA | AAGCTT | GGTCTC | ACCAGA | GTTCTTGTACGGCAATGT |
|  | DE_2__Doc-*Ct*NI_DE_3_ | Forward | ATA | GCTCTTCAAGA | GGTCTC | GGTTCA | GAAAGCAGTTCCACAGGTCTGG |
|  |  | Reverse | ATA | GCTCTTCACTT | GGTCTC | ACCAGA | ATTTCCCGAAATCGTGGGGATAGG |
|  | DE_2__Doc-*Ct*II_DE_3_ | Forward | ATA | TCTAGA | GGTCTC | GGTTCA | GGATACATTTTGCCAGACTTC |
|  |  | Reverse | ATA | AAGCTT | GGTCTC | ACCAGA | CTGTGCGTCGTAATCACTTG |
|  | DE_2__Doc-*Rf*_DE_3_ | Forward | ATA | GGATCC | GGTCTC | GGTTCA | GATAAGGATGTAGTCTAC |
|  |  | Reverse | ATA | GTCGAC | GGTCTC | ACCAGA | TTCGGGAAGCTTGTCTATGAGATG |
|  | DE_2__Doc-*Cc*_DE_3_ | Forward | ATA | GGATCC | GGTCTC | GGTTCA | CCAGTAATTGTATATGG |
|  |  | Reverse | ATA | GTCGAC | GGTCTC | ACCAGA | GTTGCTTGGAAGCTTA |
|  | DE_2__Doc-*Ac*_DE_3_ | Forward | ATA | GCTCTTCAAGA | GGTCTC | GGTTCA | CCGACACCGAAATTCATTTATGG |
|  |  | Reverse | ATA | GCTCTTCACTT | GGTCTC | ACCAGA | TTCTTCTTTTTCTTCCACCG |
|  | DE_2__Doc-*Bc*_DE_3_ | Forward | ATA | GCTCTTCAAGA | GGTCTC | GGTTCA | ATTTATCCGAAAGGCACCG |
|  |  | Reverse | ATA | GCTCTTCACTT | GGTCTC | ACCAGA | TTTCTGTTCTGCCGGAAATTCGC |
|  | DE_2__Doc-*Af*_DE_3_ | Forward | ATA | GCTCTTCAAGA | GGTCTC | GGTTCA | CAAGAAGAGGCAAATAAAGGC |
|  |  | Reverse | ATA | GCTCTTCACTT | GGTCTC | ACCAGA | TTTACCAAGCAGACC |
| 3 | DE_3__Doc-*Ct*I_DE_e_ | Forward | ATA | TCTAGA | GGTCTC | TCTGGT | GTACCTGGTACTCCTTCTAC |
|  |  | Reverse | ATA | AAGCTT | GGTCTC | ATACTT | GTTCTTGTACGGCAATGT |
|  | DE_3__Doc-*Ct*II_DE_e_ | Forward | ATA | TCTAGA | GGTCTC | TCTGGT | GGATACATTTTGCCAGACTTC |
|  |  | Reverse | ATA | AAGCTT | GGTCTC | ATACTT | CTGTGCGTCGTAATCACTTG |
|  | DE_3__Doc-*Rf*_DE_e_ | Forward | ATA | GGATCC | GGTCTC | TCTGGT | GATAAGGATGTAGTCTAC |
|  |  | Reverse | ATA | GTCGAC | GGTCTC | ATACTT | TTCGGGAAGCTTGTCTATGAGATG |
|  | DE_3__Doc-*Cc*_DE_e_ | Forward | ATA | GGATCC | GGTCTC | TCTGGT | CCAGTAATTGTATATGG |
|  |  | Reverse | ATA | GTCGAC | GGTCTC | ATACTT | GTTGCTTGGAAGCTTA |
|  | DE_3__Doc-*Ac*_DE_e_ | Forward | ATA | GCTCTTCAAGA | GGTCTC | TCTGGT | CCGACACCGAAATTCATTTATGG |
|  |  | Reverse | ATA | GCTCTTCACTT | GGTCTC | ATACTT | TTCTTCTTTTTCTTCCACCG |
|  | DE_3__Doc-*Bc*_DE_e_ | Forward | ATA | GCTCTTCAAGA | GGTCTC | TCTGGT | ATTTATCCGAAAGGCACCG |
|  |  | Reverse | ATA | GCTCTTCACTT | GGTCTC | ATACTT | TTTCTGTTCTGCCGGAAATTCGC |
|  | DE_3__Doc-*Af*_DE_e_ | Forward | ATA | GCTCTTCAAGA | GGTCTC | TCTGGT | CAAGAAGAGGCAAATAAAGGC |
|  |  | Reverse | ATA | GCTCTTCACTT | GGTCTC | ATACTT | TTTACCAAGCAGACC |

**Table S10: Overview of primers used to amplify cohesin tiles.** The first column shows the position of the tile in the final construct. The second column gives the name of the tile and the third column indicates the orientation of the primer. In the following columns, each section of the primer is shown starting from the 5’ end. All primers consist of three extra nucleotides, a type IIs recognition site and the accompanying restriction site, the BsaI recognition site, followed by the position tag and a sequence that is complementary with the fragment of interest.

| **Position** | **Tile** | **Primer** | **3 extra nt** | **Recognition (BsmbI or SapI) and restriction site** | **BsaI recognition site** | **Position tag (Sc_s_, Sc_2_, Sc_3_, Sc_4_, Sc_5_, Sc_e_)** | **Complementary sequence that hybridises with template** |
| --- | --- | --- | --- | --- | --- | --- | --- |
| 1 | Sc_s__Coh-*Ct*I_Sc_2_ | Forward | TGT | CGTCTCAGAGT | GGTCTC | ACCATG | GACGGTGTGGTAGTA |
|  |  | Reverse | TGT | CGTCTCAGAGT | GGTCTC | TGTGCT | TGTATTTGCCGGTGTGTTT |
|  | Sc_s__Coh-*Ct*II_Sc_2_ | Forward | TGT | CGTCTCAGAGT | GGTCTC | ACCATG | AGAGCTTCCAGATTCCTATGTG |
|  |  | Reverse | TGT | CGTCTCAGAGT | GGTCTC | TGTGCT | TCCGTTGGTATTGGTTCGTC |
|  | Sc_s__Coh-*Rf*_Sc_2_ | Forward | TGT | GCTCTTCAAGA | GGTCTC | ACCATG | GGCGGCCAGACATCAAAT |
|  |  | Reverse | TGT | GCTCTTCACTT | GGTCTC | TGTGCT | AGTTGTAGTTGTTGTGG |
|  | Scs_Coh-*Cc*_Sc_2_ | Forward | TGT | CGTCTCAGAGT | GGTCTC | ACCATG | CTTAAAGTTACAGTAGGAACA |
|  |  | Reverse | TGT | CGTCTCAGAGT | GGTCTC | TGTGCT | TTCCTTTGTAGGTTGAGTACC |
|  | Sc_s__Coh-*Ac*_Sc_2_ | Forward | ATA | GCTCTTCAAGA | GGTCTC | ACCATG | CTGCAGGTTGATATTG |
|  |  | Reverse | ATA | GCTCTTCACTT | GGTCTC | TGTGCT | CTGTGTTGGTGTTGGACTGGCAATAACTTCAATT |
|  | Sc_s__Coh-*Bc*_Sc_2_ | Forward | ATA | GCTCTTCAAGA | GGTCTC | ACCATG | AGCAGTCCGGGTAAC |
|  |  | Reverse | ATA | GCTCTTCACTT | GGTCTC | TGTGCT | CGTGTTCGTCGGCGTATTGGTAACGGTAATGCTG |
|  | Sc_s__Coh-*Af*_Sc_2_ | Forward | ATA | GCTCTTCAAGA | GGTCTC | ACCATG | CCGAAAACCACCATTATTGCAG |
|  |  | Reverse | ATA | GCTCTTCACTT | GGTCTC | TGTGCT | GGTGCTCGGAACCACGGCTTCCTCTTGGCTCAC |
| 2 | Sc_2__Coh-*Ct*I_Sc_3_ | Forward | TGT | CGTCTCAGAGT | GGTCTC | AGCACA | GACGGTGTGGTAGTA |
|  |  | Reverse | TGT | CGTCTCAGAGT | GGTCTC | CGTTGG | TGTATTTGCCGGTGTGTTT |
|  | Sc_2__Coh-*Ct*II_Sc_3_ | Forward | TGT | CGTCTCAGAGT | GGTCTC | AGCACA | AGAGCTTCCAGATTCCTATGTG |
|  |  | Reverse | TGT | CGTCTCAGAGT | GGTCTC | CGTTGG | TCCGTTGGTATTGGTTCGTC |
|  | Sc_2__Coh-*Rf*_Sc_3_ | Forward | TGT | GCTCTTCAAGA | GGTCTC | AGCACA | GGCGGCCAGACATCAAAT |
|  |  | Reverse | TGT | GCTCTTCACTT | GGTCTC | CGTTGG | AGTTGTAGTTGTTGTGG |
|  | Sc_2__Coh-*Cc*_Sc_3_ | Forward | TGT | CGTCTCAGAGT | GGTCTC | AGCACA | CTTAAAGTTACAGTAGGAACA |
|  |  | Reverse | TGT | CGTCTCAGAGT | GGTCTC | CGTTGG | TTCCTTTGTAGGTTGAGTACC |
|  | Sc_2__Coh-*Ac*_Sc_3_ | Forward | ATA | GCTCTTCAAGA | GGTCTC | AGCACA | CTGCAGGTTGATATTG |
|  |  | Reverse | ATA | GCTCTTCACTT | GGTCTC | CGTTGG | CTGTGTTGGTGTTGGACTGGCAATAACTTCAATT |
|  | Sc_2__Coh-*Bc*_Sc_3_ | Forward | ATA | GCTCTTCAAGA | GGTCTC | AGCACA | AGCAGTCCGGGTAAC |
|  |  | Reverse | ATA | GCTCTTCACTT | GGTCTC | CGTTGG | CGTGTTCGTCGGCGTATTGGTAACGGTAATGCTG |
|  | Sc_2__Coh-*Af*_Sc_3_ | Forward | ATA | GCTCTTCAAGA | GGTCTC | AGCACA | CCGAAAACCACCATTATTGCAG |
|  |  | Reverse | ATA | GCTCTTCACTT | GGTCTC | CGTTGG | GGTGCTCGGAACCACGGCTTCCTCTTGGCTCAC |
| 3 | Sc_3__Coh-*Ct*I_Sc_4_ | Forward | TGT | CGTCTCAGAGT | GGTCTC | CCAACG | GACGGTGTGGTAGTA |
|  |  | Reverse | TGT | CGTCTCAGAGT | GGTCTC | GCTCGT | TGTATTTGCCGGTGTGTTT |
|  | Sc_3__Coh-*Ct*II_Sc_4_ | Forward | TGT | CGTCTCAGAGT | GGTCTC | CCAACG | AGAGCTTCCAGATTCCTATGTG |
|  |  | Reverse | TGT | CGTCTCAGAGT | GGTCTC | GCTCGT | TCCGTTGGTATTGGTTCGTC |
|  | Sc_3__Coh-*Rf*_Sc_4_ | Forward | TGT | GCTCTTCAAGA | GGTCTC | CCAACG | GGCGGCCAGACATCAAAT |
|  |  | Reverse | TGT | GCTCTTCACTT | GGTCTC | GCTCGT | AGTTGTAGTTGTTGTGG |
|  | Sc_3__Coh-*Cc*_Sc_4_ | Forward | TGT | CGTCTCAGAGT | GGTCTC | CCAACG | CTTAAAGTTACAGTAGGAACA |
|  |  | Reverse | TGT | CGTCTCAGAGT | GGTCTC | GCTCGT | TTCCTTTGTAGGTTGAGTACC |
|  | Sc_3__Coh-*Ac*_Sc_4_ | Forward | ATA | GCTCTTCAAGA | GGTCTC | CCAACG | CTGCAGGTTGATATTG |
|  |  | Reverse | ATA | GCTCTTCACTT | GGTCTC | GCTCGT | CTGTGTTGGTGTTGGACTGGCAATAACTTCAATT |
|  | Sc_3__Coh-*Bc*_Sc_4_ | Forward | ATA | GCTCTTCAAGA | GGTCTC | CCAACG | AGCAGTCCGGGTAAC |
|  |  | Reverse | ATA | GCTCTTCACTT | GGTCTC | GCTCGT | CGTGTTCGTCGGCGTATTGGTAACGGTAATGCTG |
|  | Sc_3__Coh-*Af*_Sc_4_ | Forward | ATA | GCTCTTCAAGA | GGTCTC | CCAACG | CCGAAAACCACCATTATTGCAG |
|  |  | Reverse | ATA | GCTCTTCACTT | GGTCTC | GCTCGT | GGTGCTCGGAACCACGGCTTCCTCTTGGCTCAC |
| 4 | Sc_4__Coh-*Ct*I_Sc_5_ | Forward | TGT | CGTCTCAGAGT | GGTCTC | ACGAGC | GACGGTGTGGTAGTA |
|  |  | Reverse | TGT | CGTCTCAGAGT | GGTCTC | AGACGG | TGTATTTGCCGGTGTGTTT |
|  | Sc_4__Coh-*Ct*II_Sc_5_ | Forward | TGT | CGTCTCAGAGT | GGTCTC | ACGAGC | AGAGCTTCCAGATTCCTATGTG |
|  |  | Reverse | TGT | CGTCTCAGAGT | GGTCTC | AGACGG | TCCGTTGGTATTGGTTCGTC |
|  | Sc_4__Coh-*Rf*_Sc_5_ | Forward | TGT | GCTCTTCAAGA | GGTCTC | ACGAGC | GGCGGCCAGACATCAAAT |
|  |  | Reverse | TGT | GCTCTTCACTT | GGTCTC | AGACGG | AGTTGTAGTTGTTGTGG |
|  | Sc_4__Coh-*Cc*_Sc_5_ | Forward | TGT | CGTCTCAGAGT | GGTCTC | ACGAGC | CTTAAAGTTACAGTAGGAACA |
|  |  | Reverse | TGT | CGTCTCAGAGT | GGTCTC | AGACGG | TTCCTTTGTAGGTTGAGTACC |
|  | Sc_4__Coh-*Ac*_Sc_5_ | Forward | ATA | GCTCTTCAAGA | GGTCTC | ACGAGC | CTGCAGGTTGATATTG |
|  |  | Reverse | ATA | GCTCTTCACTT | GGTCTC | AGACGG | CTGTGTTGGTGTTGGACTGGCAATAACTTCAATT |
|  | Sc_4__Coh-*Bc*_Sc_5_ | Forward | ATA | GCTCTTCAAGA | GGTCTC | ACGAGC | AGCAGTCCGGGTAAC |
|  |  | Reverse | ATA | GCTCTTCACTT | GGTCTC | AGACGG | CGTGTTCGTCGGCGTATTGGTAACGGTAATGCTG |
|  | Sc_4__Coh-*Af*_Sc_5_ | Forward | ATA | GCTCTTCAAGA | GGTCTC | ACGAGC | CCGAAAACCACCATTATTGCAG |
|  |  | Reverse | ATA | GCTCTTCACTT | GGTCTC | AGACGG | GGTGCTCGGAACCACGGCTTCCTCTTGGCTCAC |
| 5 | Sc_5__Coh-*Ct*I_Sc_e_ | Forward | TGT | CGTCTCAGAGT | GGTCTC | CCGTCT | GACGGTGTGGTAGTA |
|  |  | Reverse | TGT | CGTCTCAGAGT | GGTCTC | TTACTT | GCCAACGTTAACACCACC |
|  | Sc_5__Coh-*Ct*II_Sc_e_ | Forward | TGT | CGTCTCAGAGT | GGTCTC | CCGTCT | AGAGCTTCCAGATTCCTATGTG |
|  |  | Reverse | TGT | CGTCTCAGAGT | GGTCTC | TTACTT | ACTAGCCGCCTTTATAGGCGCCGG |
|  | Sc_5__Coh-*Rf*_Sc_e_ | Forward | TGT | GCTCTTCAAGA | GGTCTC | CCGTCT | GGCGGCCAGACATCAAAT |
|  |  | Reverse | TGT | GCTCTTCACTT | GGTCTC | TTACTT | TGTAACAGGCTGCTTACCA |
|  | Sc_5__Coh-*Cc*_Sc_e_ | Forward | TGT | CGTCTCAGAGT | GGTCTC | CCGTCT | CTTAAAGTTACAGTAGGAACA |
|  |  | Reverse | TGT | CGTCTCAGAGT | GGTCTC | TTACTT | TACACTACCGTTTGTCTTAGT |
|  | Sc_5__Coh-*Ac*_Sc_e_ | Forward | ATA | GCTCTTCAAGA | GGTCTC | CCGTCT | CTGCAGGTTGATATTG |
|  |  | Reverse | ATA | GCTCTTCACTT | GGTCTC | TTACTT | GGCAATAACTTCAATTTTGCC |
|  | Sc_5__Coh-*Bc*_Sc_e_ | Forward | ATA | GCTCTTCAAGA | GGTCTC | CCGTCT | AGCAGTCCGGGTAAC |
|  |  | Reverse | ATA | GCTCTTCACTT | GGTCTC | TTACTT | GGCTTCCTCTTGGCTCAC |
|  | Sc_5__Coh-*Af*_Sc_e_ | Forward | ATA | GCTCTTCAAGA | GGTCTC | CCGTCT | CCGAAAACCACCATTATTGCAG |
|  |  | Reverse | ATA | GCTCTTCACTT | GGTCTC | TTACTT | ATTGGTAACGGTAATGC |

**Table 11: Overview of primers used to amplify CBM tiles.** The first column shows the position of the tile in the final construct. The second column gives the name of the tile and the third column indicates the orientation of the primer. In the following columns, each section of the primer is shown starting from the 5’ end. All primers consist of three extra nucleotides, a type IIs recognition site and the accompanying restriction site, the BsaI recognition site, followed by the position tag and a sequence that is complementary with the fragment of interest. Point mutations in the complementary sequence are shown in red.

| **Position** | **Tile** | **Primer** | **3 extra nt** | **Recognition (BsmbI or SapI) and restriction site** | **BsaI recognition site** | **Position tag (Sc_s_, Sc_2_, Sc_3_, Sc_4_, Sc_5_, Sc_e_)** | **Complementary sequence that hybridises with template** |
| --- | --- | --- | --- | --- | --- | --- | --- |
| 1 | Sc_s__*Ct*-CBM3_Sc_2_ | Forward | TGT | CGTCTCAGAGT | GGTCTC | ACCATG | GTATCAGGCAATTTGAAGGTT |
|  |  | Reverse | TGT | CGTCTCAGAGT | GGTCTC | TGTGCT | CGGATCATCTGACGGCGGTA |
|  | Sc_s__*Cj*-CBM35_Sc_2_ | Forward | ATA | GCTCTTCAAGA | GGTCTC | ACCATG | GCAGTACCGGAAGGCAATAGC |
|  |  | Reverse | ATA | GCTCTTCACTT | GGTCTC | TGTGCT | GCTACCCGAAGGTAATTGCG |
| 2 | Sc_2__*Ct*-CBM3_Sc_3_ | Forward | TGT | CGTCTCAGAGT | GGTCTC | AGCACA | GTATCAGGCAATTTGAAGGTT |
|  |  | Reverse | TGT | CGTCTCAGAGT | GGTCTC | CGTTGG | CGGATCATCTGACGGCGGTA |
|  | Sc_2__*Cj*-CBM35_Sc_3_ | Forward | ATA | GCTCTTCAAGA | GGTCTC | AGCACA | GCAGTACCGGAAGGCAATAGC |
|  |  | Reverse | ATA | GCTCTTCACTT | GGTCTC | CGTTGG | GCTACCCGAAGGTAATTGCG |
| 3 | Sc_3__*Ct*-CBM3_Sc_4_ | Forward | TGT | CGTCTCAGAGT | GGTCTC | CCAACG | GTATCAGGCAATTTGAAGGTT |
|  |  | Reverse | TGT | CGTCTCAGAGT | GGTCTC | GCTCGT | CGGATCATCTGACGGCGGTA |
|  | Sc_3__*Cj*-CBM35_Sc_4_ | Forward | ATA | GCTCTTCAAGA | GGTCTC | CCAACG | GCAGTACCGGAAGGCAATAGC |
|  |  | Reverse | ATA | GCTCTTCACTT | GGTCTC | GCTCGT | GCTACCCGAAGGTAATTGCG |
| 4 | Sc_4__*Ct*-CBM3_Sc_5_ | Forward | TGT | CGTCTCAGAGT | GGTCTC | ACGAGC | GTATCAGGCAATTTGAAGGTT |
|  |  | Reverse | TGT | CGTCTCAGAGT | GGTCTC | AGACGG | CGGATCATCTGACGGCGGTA |
|  | Sc_4__*Cj*-CBM35_Sc_5_ | Forward | ATA | GCTCTTCAAGA | GGTCTC | ACGAGC | GCAGTACCGGAAGGCAATAGC |
|  |  | Reverse | ATA | GCTCTTCACTT | GGTCTC | AGACGG | GCTACCAGAAGGTAATTGCG |
| 5 | Sc_5__*Ct*-CBM3_Sc_e_ | Forward | TGT | CGTCTCAGAGT | GGTCTC | CCGTCT | GTATCAGGCAATTTGAAGGTT |
|  |  | Reverse | TGT | CGTCTCAGAGT | GGTCTC | TTACTT | ACCGGGTTCTTTACCCCATAC |
|  | Sc_5__*Cj*-CBM35_Sc_e_ | Forward | ATA | GCTCTTCAAGA | GGTCTC | CCGTCT | GCAGTACCGGAAGGCAATAGC |
|  |  | Reverse | ATA | GCTCTTCACTT | GGTCTC | TTACTT | GTTGGCTGGCGAGCG |

**Table S12: Overview of primers used to amplify linker tiles.** The first column shows the position of the tile in the final construct. The second column gives the name of the tile and the third column indicates the orientation of the primer. In the following columns, each section of the primer is shown starting from the 5’ end. All primers consist of three extra nucleotides, a restriction/recognition site of a type IIs restriction enzyme, the BsaI recognition site, followed by the position tag and a sequence that is complementary with the fragment of interest.

| **Position** | **Tile** | **Primer** | **3 extra nt** | **Recognition (SapI) and restriction site** | **BsaI recognition site** | **Position tag (DE_s_, DE_2_, DE_3_, DE_e_)** | **Complementary sequence that hybridises with template** |
| --- | --- | --- | --- | --- | --- | --- | --- |
| 2 | Li-A | Forward | ATA | GCTCTTCAAGA | GGTCTC | GGTTCA | CTGTCTCGTTTCTTCCACG |
|  |  | Reverse | ATA | GCTCTTCACTT | GGTCTC | ACCAGA | CAGTTCCGCGTGGAAGAAACGAG |
|  | Li-B | Forward | ATA | GCTCTTCAAGA | GGTCTC | GGTTCA | GTTTTCAACCAGCGTAAAGAACACAAAGG |
|  |  | Reverse | ATA | GCTCTTCACTT | GGTCTC | ACCAGA | CGCCAGCATGTAACCTTTGTGTTCTTTACGC |
|  | Li-C | Forward | ATA | GCTCTTCAAGA | GGTCTC | GGTTCA | ATCCCGCAGGGTCGTTCTCACCCGGTTCAGC |
|  |  | Reverse | ATA | GCTCTTCACTT | GGTCTC | ACCAGA | GAACGCACCCGGGTACGGCTGAACCGGGTGAG |
|  | Li-D | Forward | ATA | GCTCTTCAAGA | GGTCTC | GGTTCA | CCGGCGGTTCCGCCGCCG |
|  |  | Reverse | ATA | GCTCTTCACTT | GGTCTC | ACCAGA | CGGCGGCGGAACCGCCGG |
|  | Li-E | Forward | ATA | GCTCTTCAAGA | GGTCTC | GGTTCA | GGCGGTGGCGGTAGCGGTGGCGGTGGCTCTGG |
|  |  | Reverse | ATA | GCTCTTCACTT | GGTCTC | ACCAGA | GCTACCGCCACCACCAGAGCCACCGCCACC |
|  | Li-F | Forward | ATA | GCTCTTCAAGA | GGTCTC | GGTTCA | GGCGGCGGCGGTGGCGGCGGCGGT |
|  |  | Reverse | ATA | GCTCTTCACTT | GGTCTC | ACCAGA | ACCGCCGCCGCCACCGCCGCCGCC |
|  | Li-G | Forward | ATA | GCTCTTCAAGA | GGTCTC | GGTTCA | GAAGCGGCGGCCAAAGAAGCGGCAGCGAAGG |
|  |  | Reverse | ATA | GCTCTTCACTT | GGTCTC | ACCAGA | TTTCGCCGCGGCTTCCTTCGCTGCCGCTTC |
|  | Li-H | Forward | ATA | GCTCTTCAAGA | GGTCTC | GGTTCA | GCGCCGGCCCCGGCACCGGCCCCGGCGCCG |
|  |  | Reverse | ATA | GCTCTTCACTT | GGTCTC | ACCAGA | CGGCGCCGGGGCCGGTGCCGGGGCCGGCGC |
|  | Li-I | Forward | ATA | GCTCTTCAAGA | GGTCTC | GGTTCA | GAAGCGGCGGCGAAAGAAGCGGCGAAAGAAGCGGCGAAA |
|  |  | Reverse | ATA | GCTCTTCACTT | GGTCTC | ACCAGA | TTTCGCCGCTTCTTTCGCCGCTTCTTTCGCCGCCGCTTC |

**Table S13: Overview of primers used to amplify tag tiles.** The first column shows the name of the tile and the second column indicates the orientation of the primer. In the following columns, each section of the primer is shown starting from the 5’ end. All primers consist of three extra nucleotides, a type IIs recognition site and the accompanying restriction site, the BsaI recognition site, followed by the position tag and a sequence that is complementary with the fragment of interest.

| **Tile** | **Primer** | **3 extra nt** | **Recognition (SapI) and restriction site** | **BsaI recognition site** | **Position tag (DE_s_, DE_2_, DE_3_, DE_e_, Sc_s_, Sc_2_, Sc_3_, Sc_4_, Sc_5_, Sc_e_)** | **Complementary sequence** |
| --- | --- | --- | --- | --- | --- | --- |
| DE_s__His_DE_2_ | Forward | ATA | GCTCTTCAAGA | GGTCTC | ACCATG | CATCATCACCATCACCAT |
|  | Reverse | ATA | GCTCTTCACTT | GGTCTC | TGAACC | ATGGTGATGGTGATGATG |
| DE_3__His_DE_e_ | Forward | ATA | GCTCTTCAAGA | GGTCTC | TCTGGT | CATCATCACCATCACCAT |
|  | Reverse | ATA | GCTCTTCACTT | GGTCTC | ATACTT | ATGGTGATGGTGATGATG |
| DE_s__His_DE_3_ | Forward | ATA | GCTCTTCAAGA | GGTCTC | ACCATG | CATCATCACCATCACCAT |
|  | Reverse | ATA | GCTCTTCACTT | GGTCTC | ACCAGA | ATGGTGATGGTGATGATG |
| DE_2__His_DE_e_ | Forward | ATA | GCTCTTCAAGA | GGTCTC | GGTTCA | CATCATCACCATCACCAT |
|  | Reverse | ATA | GCTCTTCACTT | GGTCTC | ATACTT | ATGGTGATGGTGATGATG |
| Sc_2__His_Sc_e_ | Forward | ATA | GCTCTTCAAGA | GGTCTC | AGCACA | CATCATCACCATCACCAT |
|  | Reverse | ATA | GCTCTTCACTT | GGTCTC | TTACTT | ATGGTGATGGTGATGATG |
| Sc_3__His_Sc_e_ | Forward | ATA | GCTCTTCAAGA | GGTCTC | CCAACG | CATCATCACCATCACCAT |
|  | Reverse | ATA | GCTCTTCACTT | GGTCTC | TTACTT | ATGGTGATGGTGATGATG |
| Sc_4__His_Sc_e_ | Forward | ATA | GCTCTTCAAGA | GGTCTC | ACGAGC | CATCATCACCATCACCAT |
|  | Reverse | ATA | GCTCTTCACTT | GGTCTC | TTACTT | ATGGTGATGGTGATGATG |
| Sc_5__His_Sc_e_ | Forward | ATA | GCTCTTCAAGA | GGTCTC | CCGTCT | CATCATCACCATCACCAT |
|  | Reverse | ATA | GCTCTTCACTT | GGTCTC | TTACTT | ATGGTGATGGTGATGATG |
| Sc_s__His_Sc_2_ | Forward | ATA | GCTCTTCAAGA | GGTCTC | ACCATG | CATCATCACCATCACCAT |
|  | Reverse | ATA | GCTCTTCACTT | GGTCTC | TGTGCT | ATGGTGATGGTGATGATG |
| Sc_s__His_Sc_3_ | Forward | ATA | GCTCTTCAAGA | GGTCTC | ACCATG | CATCATCACCATCACCAT |
|  | Reverse | ATA | GCTCTTCACTT | GGTCTC | CGTTGG | ATGGTGATGGTGATGATG |
| Sc_s__His_Sc_4_ | Forward | ATA | GCTCTTCAAGA | GGTCTC | ACCATG | CATCATCACCATCACCAT |
|  | Reverse | ATA | GCTCTTCACTT | GGTCTC | GCTCGT | ATGGTGATGGTGATGATG |
| Sc_s__His_Sc_5_ | Forward | ATA | GCTCTTCAAGA | GGTCTC | ACCATG | CATCATCACCATCACCAT |
|  | Reverse | ATA | GCTCTTCACTT | GGTCTC | AGACGG | ATGGTGATGGTGATGATG |
| Sc_s__His_GST_DEVD_Sc_2_ | Forward | ATA | GCTCTTCAAGA | GGTCTC | ACCATG | CATCATCACCATCACCAT |
|  | Reverse | ATA | GCTCTTCACTT | GGTCTC | TGTGCT | ATCCACTTCGTCCGA |
| Sc_s__GST_DEVD_Sc_2_ | Forward | ATA | GCTCTTCAAGA | GGTCTC | ACCATG | CCTATACTAGGTTATTGG |
|  | Reverse | ATA | GCTCTTCACTT | GGTCTC | TGTGCT | ATCCACTTCGTCCGA |
| Sc_s__StrepTagII_Sc_2_ | Forward | ATA | GCTCTTCAAGA | GGTCTC | ACCATG | TGGAGCCACCCGCAGTTCG |
|  | Reverse | ATA | GCTCTTCACTT | GGTCTC | TGTGCT | TTTTTCGAACTGCGGGTGGCT |

**Table S14: Overview of primers used to amplify tiles encoding GM-degrading enzymes.** The first column shows the position of the tile in the final construct. The second column gives the name of the tile and the third column indicates the orientation of the primer. In the following columns, each section of the primer is shown starting from the 5’ end. All primers consist of three extra nucleotides, a restriction/recognition site of a specific restriction enzyme, the BsaI recognition site, followed by the position tag and a sequence that is complementary with the fragment of interest.

| **Position** | **Tile** | **Primer** | **3 extra nt** | **Recognition (BsmbI, SapI or BpiI) and restriction site** | **BsaI recognition site** | **Position tag (DE_s_, DE_2_, DE_3_, De_e_)** | **Complementary sequence that hybridises with template** |
| --- | --- | --- | --- | --- | --- | --- | --- |
| 1 | DE_s__*Tf*-Manna-S_DE_2_ | Forward | ATA | CGTCTCAGAGT | GGTCTC | ACCATG | GCCACCGGGCTCCACGTCAAGAACG |
|  |  | Reverse | ATA | CGTCTCAGAGT | GGTCTC | TGAACC | GCTCAGGTTGTCGCCGTCGAAG |
|  | DE_s__*Tf*-Manna_DE_2_ | Forward | ATA | CGTCTCAGAGT | GGTCTC | ACCATG | GCCACCGGGCTCCACGTCAAGAACG |
|  |  | Reverse | ATA | CGTCTCAGAGT | GGTCTC | TGAACC | GCCGCCGAAGATCGTGGCCTCCTTGG |
|  | DE_s__*Tf*-Manna-Li-DE_2_ | Forward | ATA | CGTCTCAGAGT | GGTCTC | ACCATG | GCCACCGGGCTCCACGTCAAGAACG |
|  |  | Reverse | ATA | CGTCTCAGAGT | GGTCTC | TGAACC | CTCCGCGGGAGGCGGAGTCGGAGT |
|  | DE_s__*Tf*-Manna-CBM_DE_2_ | Forward | ATA | CGTCTCAGAGT | GGTCTC | ACCATG | GCCACCGGGCTCCACGTCAAGAACG |
|  |  | Reverse | ATA | CGTCTCAGAGT | GGTCTC | TGAACC | GCGAGCGGTGCAGCTCAGCGTCAG |
|  | DE_s__*Tf*-Manno_DE_2_ | Forward | ATA | GCTCTTCAAGA | GGTCTC | ACCATG | ATGCAACACCGCTTCACCTGTGG |
|  |  | Reverse | ATA | GCTCTTCACTT | GGTCTC | TGAACC | GACCGGGATGCTGTCCTG |
|  | DE_s__*Ba*-Aga_DE_2_ | Forward | ATA | GAAGACGTTAGA | GGTCTC | ACCATG | ATGACGCTCATTCAGACTTTCCAC |
|  |  | Reverse | ATA | GAAGACTAGCTT | GGTCTC | TGAACC | GATGCGGACTACTTTGAAAAGGAC |
|  | DE_s__*Cc*-Aga_DE_2_ | Forward | ATA | GCTCTTCAAGA | GGTCTC | ACCATG | GATAACGGTCTTGCAAAAACACC |
|  |  | Reverse | ATA | GCTCTTCACTT | GGTCTC | TGAACC | TTTAGCCCACAAATCCCT |
|  | DE_s__*Cc*-Aga-Li-DE_2_ | Forward | ATA | GCTCTTCAAGA | GGTCTC | ACCATG | GATAACGGTCTTGCAAAAACACC |
|  |  | Reverse | ATA | GCTCTTCACTT | GGTCTC | TGAACC | TATTTGCTTTGTTGCATCAACAGG |
|  | DE_s__*Cc*-Aga-CBM_DE_2_ | Forward | ATA | GCTCTTCAAGA | GGTCTC | ACCATG | GATAACGGTCTTGCAAAAACACC |
|  |  | Reverse | ATA | GCTCTTCACTT | GGTCTC | TGAACC | TCCTGTTGCCCCGCTGAC |
|  | DE_s__*Cj*-Aga_DE_2_ | Forward | ATA | GCTCTTCAAGA | GGTCTC | ACCATG | CAAAAATTTGAGCAACTCGC |
|  |  | Reverse | ATA | GCTCTTCACTT | GGTCTC | TGAACC | ACGCGGGCTTAAACG |
| 2 | DE_2__*Tf*-Manna-S_DE_3_ | Forward | ATA | CGTCTCAGAGT | GGTCTC | GGTTCA | GCCACCGGGCTCCACGTCAAGAACG |
|  |  | Reverse | ATA | CGTCTCAGAGT | GGTCTC | ACCAGA | GCTCAGGTTGTCGCCGTCGAAG |
|  | DE_2__*Tf*-Manna-Li-DE_3_ | Forward | ATA | CGTCTCAGAGT | GGTCTC | GGTTCA | GCCACCGGGCTCCACGTCAAGAACG |
|  |  | Reverse | ATA | CGTCTCAGAGT | GGTCTC | ACCAGA | CTCCGCGGGAGGCGGAGTCGGAGT |
|  | DE_2__*Tf*-Manna-CBM_DE_3_ | Forward | ATA | CGTCTCAGAGT | GGTCTC | GGTTCA | GCCACCGGGCTCCACGTCAAGAACG |
|  |  | Reverse | ATA | CGTCTCAGAGT | GGTCTC | ACCAGA | GCGAGCGGTGCAGCTCAGCGTCAG |
|  | DE_2__*Tf*-Manno_DE_3_ | Forward | ATA | GCTCTTCAAGA | GGTCTC | GGTTCA | ATGCAACACCGCTTCACCTGTGG |
|  |  | Reverse | ATA | GCTCTTCACTT | GGTCTC | ACCAGA | GACCGGGATGCTGTCCTG |
|  | DE_2__*Ba*-Aga_DE_3_ | Forward | ATA | GAAGACGTTAGA | GGTCTC | GGTTCA | ATGACGCTCATTCAGACTTTCCAC |
|  |  | Reverse | ATA | GAAGACTAGCTT | GGTCTC | ACCAGA | GATGCGGACTACTTTGAAAAGGAC |
|  | DE_2__*Cc*-Aga_DE_3_ | Forward | ATA | GCTCTTCAAGA | GGTCTC | GGTTCA | GATAACGGTCTTGCAAAAACACC |
|  |  | Reverse | ATA | GCTCTTCACTT | GGTCTC | ACCAGA | TTTAGCCCACAAATCCCT |
|  | DE_2__*Cc*-Aga-Li-DE_3_ | Forward | ATA | GCTCTTCAAGA | GGTCTC | GGTTCA | GATAACGGTCTTGCAAAAACACC |
|  |  | Reverse | ATA | GCTCTTCACTT | GGTCTC | ACCAGA | TATTTGCTTTGTTGCATCAACAGG |
|  | DE_2__*Cc*-Aga-CBM_DE_3_ | Forward | ATA | GCTCTTCAAGA | GGTCTC | GGTTCA | GATAACGGTCTTGCAAAAACACC |
|  |  | Reverse | ATA | GCTCTTCACTT | GGTCTC | ACCAGA | TCCTGTTGCCCCGCTGAC |
|  | DE_2__*Cj*-Aga_DE_3_ | Forward | ATA | GCTCTTCAAGA | GGTCTC | GGTTCA | CAAAAATTTGAGCAACTCGC |
|  |  | Reverse | ATA | GCTCTTCACTT | GGTCTC | ACCAGA | ACGCGGGCTTAAACG |
| 3 | DE_3__*Tf*-Manna_DE_e_ | Forward | ATA | CGTCTCAGAGT | GGTCTC | TCTGGT | GCCACCGGGCTCCACGTCAAGAACG |
|  |  | Reverse | ATA | CGTCTCAGAGT | GGTCTC | ATACTT | GCTCAGGTTGTCGCCGTCGAAG |
|  | DE_3__*Tf*-Manna-Li-DE_e_ | Forward | ATA | CGTCTCAGAGT | GGTCTC | TCTGGT | GCCACCGGGCTCCACGTCAAGAACG |
|  |  | Reverse | ATA | CGTCTCAGAGT | GGTCTC | ATACTT | CTCCGCGGGAGGCGGAGTCGGAGT |
|  | DE_3__*Tf*-Manna-CBM_DE_e_ | Forward | ATA | CGTCTCAGAGT | GGTCTC | TCTGGT | GCCACCGGGCTCCACGTCAAGAACG |
|  |  | Reverse | ATA | CGTCTCAGAGT | GGTCTC | ATACTT | GCGAGCGGTGCAGCTCAGCGTCAG |
|  | DE_3__*Tf*-Manno_DE_e_ | Forward | ATA | GCTCTTCAAGA | GGTCTC | TCTGGT | ATGCAACACCGCTTCACCTGTGG |
|  |  | Reverse | ATA | GCTCTTCACTT | GGTCTC | ATACTT | GACCGGGATGCTGTCCTG |
|  | DE_3__*Ba*-Aga_DE_e_ | Forward | ATA | GAAGACGTTAGA | GGTCTC | TCTGGT | ATGACGCTCATTCAGACTTTCCAC |
|  |  | Reverse | ATA | GAAGACTAGCTT | GGTCTC | ATACTT | GATGCGGACTACTTTGAAAAGGAC |
|  | DE_3__*Cc*-Aga_DE_e_ | Forward | ATA | GCTCTTCAAGA | GGTCTC | TCTGGT | GATAACGGTCTTGCAAAAACACC |
|  |  | Reverse | ATA | GCTCTTCACTT | GGTCTC | ATACTT | TTTAGCCCACAAATCCCT |
|  | DE_3__*Cc*-Aga-Li-DE_e_ | Forward | ATA | GCTCTTCAAGA | GGTCTC | TCTGGT | GATAACGGTCTTGCAAAAACACC |
|  |  | Reverse | ATA | GCTCTTCACTT | GGTCTC | ATACTT | TATTTGCTTTGTTGCATCAACAGG |
|  | DE_3__*Cc*-Aga-CBM_DE_e_ | Forward | ATA | GCTCTTCAAGA | GGTCTC | TCTGGT | GATAACGGTCTTGCAAAAACACC |
|  |  | Reverse | ATA | GCTCTTCACTT | GGTCTC | ATACTT | TCCTGTTGCCCCGCTGAC |
|  | DE_3__*Cj*-Aga_DE_e_ | Forward | ATA | GCTCTTCAAGA | GGTCTC | TCTGGT | CAAAAATTTGAGCAACTCGC |
|  |  | Reverse | ATA | GCTCTTCACTT | GGTCTC | ATACTT | ACGCGGGCTTAAACG |

**Table S15: Primers used in the inverse PCR to remove the BsaI recognition site.** The first column indicates the tile that contains the BsaI recognition site. The second column indicates the orientation of the primer. In the following columns, each section of the primer is shown starting from the 5’ end. All primers consist of three extra nucleotides, an SapI recognition site, followed by an extra nucleotide and the template complementary sequence including the point mutation.

| **Tile** | **Primer** | **3 extra nt** | **SapI recognition site** | **Complementary sequence including the point mutation** |
| --- | --- | --- | --- | --- |
| *Tf*-Manno | Forward | TTT | GCTCTTCA | GAAACCGAGCTCGGCTGGATC |
|  | Reverse | TTT | GCTCTTCA | TTCGTTGCGACCTTGGTAAGG |
| *Cc*-Gal | Forward | TTT | GCTCTTCA | GGCCTCAAACTAGGGATATAC |
|  | Reverse | CAC | GCTCTTCA | GCCTTTTGCATGTACATAATC |

**References**

1. Hilge M, Gloor SM, Rypniewski W, Sauer O, Heightman TD, Zimmermann W, Winterhalter K, Piontek K. High-resolution native and complex structures of thermostable beta-mannanase from *Thermomonospora fusca* - Substrate specificity in glycosyl hydrolase family 5. Structure. 1998; 6(11): 1433-1444.

2. Toth A, Barna T, Szabo E, Elek R, Hubert A, Nagy I, Kriszt B, Tancsics A, Kukolya J. Cloning, expression and biochemical characterization of endomannanases from *Thermobifida* species isolated from different niches. PLoS One. 2016; e0155769.

3. Beki E, Nagy S, Vanderleyden J, Jager S, Kiss L, Fulop L, Hornok L, Kukolya J. Cloning and heterologous expression of a beta-D-mannosidase (EC 3.2.1.25)-encoding gene from *Thermobifida fusca* TM51. Appl Environ Microbiol. 2003; 69(4): 1944-1952.

4. Leder S, Hartmeier W, Marx SP. Alpha-galactosidase of *Bifidobacterium adolescentis* DSM 20083. Curr Microbiol. 1999; 38(2): 101-106.

5. Van Laere KMJ, Hartemink R, Beldman G, Pitson S, Dijkema C, Schols HA, Voragen AGJ. Transglycosidase activity of *Bifidobacterium adolescentis* DSM 20083 alpha-galactosidase. Appl Microbiol Biot. 1999; 52(5): 681-688.

6. Halstead JR, Fransen MP, Eberhart RY, Park AJ, Gilbert HJ, Hazlewood GP. Alpha-galactosidase A from *Pseudomonas fluorescens* subsp *cellulosa*: cloning, high level expression and its role in galactomannan hydrolysis. FEMS Microbiol Lett. 2000; 192(2): 197-203.
